# Anatomy of *Parahesperornis:* Evolutionary Mosaicism in the Cretaceous Hesperornithiformes (Aves)

**DOI:** 10.3390/life10050062

**Published:** 2020-05-14

**Authors:** Alyssa Bell, Luis M. Chiappe

**Affiliations:** The Dinosaur Institute, Natural History Museum of Los Angeles County, 900 Exposition Blvd, Los Angeles, CA 90007, USA; lchiappe@nhm.org

**Keywords:** Aves, Hesperornithiformes, *Hesperornis*, *Parahesperornis*, Cretaceous, Niobrara Formation, Smoky Hills Member

## Abstract

The Hesperornithiformes constitute the first known avian lineage to secondarily lose flight in exchange for the evolution of a highly derived foot-propelled diving lifestyle, thus representing the first lineage of truly aquatic birds. First unearthed in the 19th century, and today known from numerous Late Cretaceous (Cenomanian-Maastrichtian) sites distributed across the northern hemisphere, these toothed birds have become icons of early avian evolution. Initially erected as a taxon in 1984 by L. D. Martin, *Parahesperornis alexi* is known from the two most complete hesperornithiform specimens discovered to date and has yet to be fully described. *P. alexi* thus contributes significantly to our understanding of hesperornithiform birds, despite often being neglected in favor of the iconic *Hesperornis.* Here, we present a full anatomical description of *P. alexi* based upon the two nearly complete specimens in the collections of the University of Kansas Natural History Museum, as well as an extensive comparison to other hesperornithiform taxa. This study reveals *P. alexi* to possess a mosaic of basal and derived traits found among other hesperornithiform taxa, indicating a transitional form in the evolution of these foot-propelled diving birds. This study describes broad evolutionary patterns within the Hesperornithiformes, highlighting the significance of these birds as not only an incredible example of the evolution of ecological specializations, but also for understanding modern bird evolution, as they are the last known divergence of pre-modern bird diversification.

## 1. Introduction

**Figure 1:**
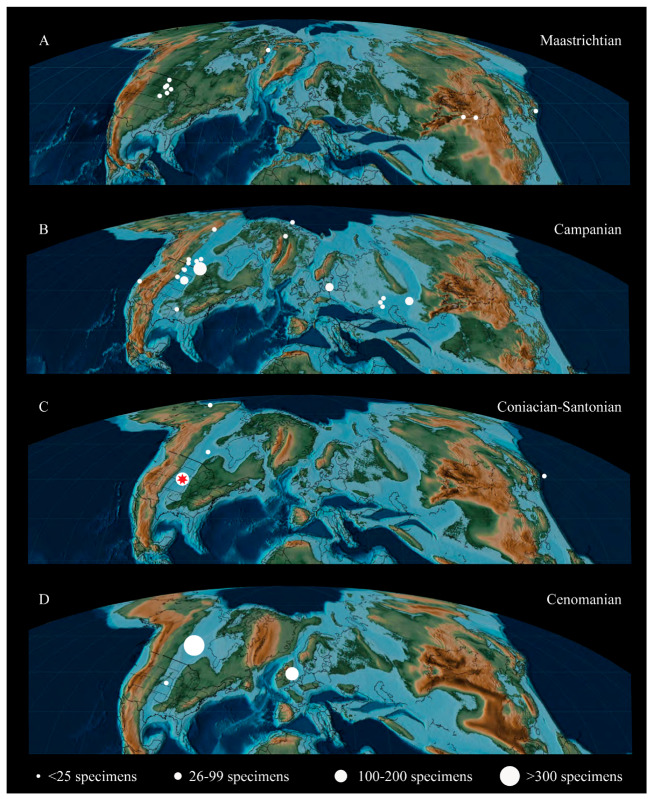
Global distribution of hesperornithiform localities, scaled to approximate number of specimens documented in the literature [3,5,6,7,8,9,10,12,21,22,23,24,25,26,27,28,29,30,31,32,33,34,35,36,37,38,39,40,41,42,43,44,45,46,47,48,49,50,51] and museum collections, mapped on paleogeographic reconstructions of the Cretaceous [52].

The Hesperornithiformes, a group of foot-propelled diving birds from the Cretaceous, constitute a highly specialized lineage of birds outside Neornithes and the earliest to become fully adapted for living in the marine realm. Despite being one of the better-known clades of early birds, the Hesperornithiformes (sometimes referred to as Hesperornithes [1,2]) suffer from a historic dearth of detailed anatomical research. First identified in the late 1800s in a series of short papers [3,4,5,6] and one monograph [7], subsequent research was primarily focused on brief descriptions of new fossil discoveries (for example [8,9,10,11,12]) or the relationships of the group to modern birds (for example [13,14,15]). Only recently have more comprehensive studies emerged assessing the systematics [12,16] or ecology [17,18] of the Hesperornithiformes, and only certain species have been subject to thorough study (for example, *Hesperornis regalis* [7]; *Baptornis advenus* [19]; *Fumicollis hoffmani* [20]). Today, hesperornithiforms have a fossil record that extends throughout the Late Cretaceous across Laurasia (Figure 1).

The history of the study of *Parahesperornis alexi*, a medium-sized hesperornithiform from the Smoky Hill Member of the Niobrara Chalk Formation (late Coniacian to early Campanian, USA) of western Kansas, is illustrative of the lack of detailed studies of the morphology of these early birds. The holotype of *Parahesperornis alexi*, University of Kansas Museum of Natural History (KUVP) specimen KUVP 2287, was discovered in 1894 but not described until 1984, when L. D. Martin published a brief report presenting a sample of the unique morphological features of this bird and identifying it as an evolutionary intermediate between *Hesperornis* and *Baptornis* [40].

The time lag between the discovery of KUVP 2287 and its identification as a new taxon may be due to the misidentification of the specimen by Williston [53,54], who assigned it to *Hesperornis gracilis*. *H. gracilis* was described by Marsh [5] from an isolated tarsometatarsus (Yale Peabody Museum [YPM] specimen YPM 1473), which is only slightly smaller than *H. regalis* and largely similar in overall morphology. It is unclear how Williston identified KUVP 2287 as “possibly [being] identical to *H. gracilis*” [53], as KUVP 2287 is notably smaller than *H. gracilis* and differs markedly in the morphology of the distal tarsometatarsus. Following this mistaken identification, Lucas [13] erroneously used features of KUVP 2287 to erect a new genus, *Hargeria*, to which he reassigned *Hesperornis gracilis*, stating that the differences between *H. gracilis* (based on KUVP 2287) and *H. regalis* were great enough to warrant generic distinction. Comparison of YPM 1473 to KUVP 2287 reveals that the two specimens are clearly dissimilar, and KUVP 2287 is in no way assignable to *H. gracilis*, thus invalidating Lucas’s creation of *Hargeria* [26,40], as per the ICZN’s code (Article 49) [55].

While Martin’s [40] original note erecting *Parahesperornis alexi* indicated additional work was forthcoming, the full description was never completed. Several papers referred to the skull of KUVP 2287 in analyses of the hesperornithiform skull [56,57], often with the erroneous identification of *H. gracilis* [13,58,59], and so a full description of the skull was never undertaken. A single paper [26] reporting the discovery of an isolated tarsometatarsus (Sternberg Museum of Natural History (FHSM) specimen FHSM VP-17312) assigned to *Parahesperornis* is the only other work that was published on this genus since Martin’s [40] work. Therefore, this study presents the first detailed anatomical analysis of *Parahesperornis alexi* based upon the holotype specimen, KUVP 2287, as well as a second exceptionally preserved specimen, KUVP 24090, that has not been previously published.

**Figure 2:**
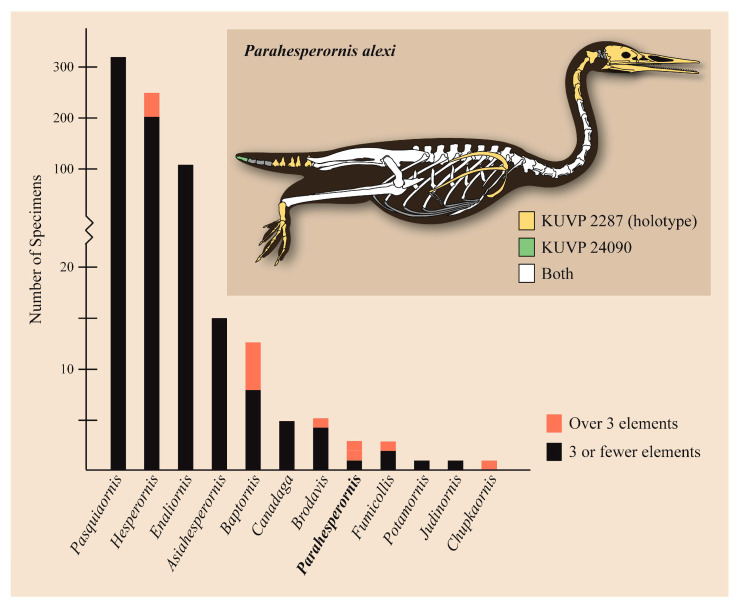
Completeness of hesperornithiform specimens. Inset shows reconstruction of *Parahesperornis alexi*, with elements known from KUVP 2287 (**yellow**), KUVP 24090 (**green**), and both specimens (**white**) shaded.

These two specimens are particularly important considering typical hesperornithiform fossil specimens, which are often highly fragmentary, isolated elements. Of the over twenty hesperornithiform species that have been described, only four have assigned specimens consisting of more than three elements. Of these four species, only two have any specimens that are over 50% complete. One of these is the type species of the family Hesperornithidae, *Hesperornis regalis*, and the other is *Parahesperornis alexi*. While *Hesperornis* has been extensively studied and is very well known in the scientific literature, *Parahesperornis* has been neglected. As a result, two of the best preserved hesperornithiform specimens, KUVP 2287 and KUVP 24090, have never been fully described or illustrated in the literature. Between these two specimens, almost the entire skeleton of *Parahesperornis alexi* is known, as well as impressions of the skin of the foot of this bird, thus rendering a great deal of anatomical information about these highly specialized, extinct birds (Figure 2).

This monograph is dedicated to the late L. D. Martin, whose studies on hesperornithiforms contributed greatly to reigniting scientific interest in these fascinating birds, and who generously spent time in discussion of these birds with one of the authors (A. Bell) during her many research trips to the University of Kansas.

### Phylogenetic Relationships

In Martin’s [40] original work on *Parahesperornis alexi* he identified the species as an evolutionary intermediate between *Baptornis* and *Hesperornis*. Martin [40] based his conclusion on advanced toe rotation, as indicated by the similar morphology of the metatarsal trochleae in *Hesperornis* and *Parahesperornis*, and an interpreted lack of fusion between the frontals and parietals of *Parahesperornis*, a condition assumed to be more primitive than the fused condition seen in *Hesperornis*. However, Martin’s analysis was not a quantitative evaluation of a character matrix, as we understand phylogenetics today, but rather an anecdotal hypothesis based on a few observed features.

It was only more recently that *Parahesperornis* was included in modern phylogenetic analyses. First, an analysis examining relationships among the major clades of Mesozoic birds was presented by O’Connor et al. [60] This study found *Parahesperornis* to be the sister taxon to *Hesperornis*, forming the most derived clade of hesperornithiforms, a position supported by later analyses [12,16].

Within Ornithuromorpha, the Hesperornithiformes is consistently recovered as a monophyletic clade close to crown birds [2,12,16,60,61]. However, some studies recover the Hesperornithiformes as more closely related to Neornithes than is *Ichthyornis* [16,60,61,62,63], but others recover the Hesperornithiformes as the sister clade to Neornithes+*Ichthyornis* [2,12,64]. Agreement between analyses based on newly recovered material of *Ichthyornis* [61], and those designed to assess the placement of the Hesperornithiformes in particular amongst other Ornithuromorphs [16,60] provides strong support for interpreting the Hesperornithiformes as the closest outgroup of modern birds. As such, they are essential to understanding the origin of Neornithes. While much work has been done focusing on the highly derived diving adaptations of these birds, as a group they are also broadly relevant to our understanding of the evolution of modern birds.

**Figure 3:**
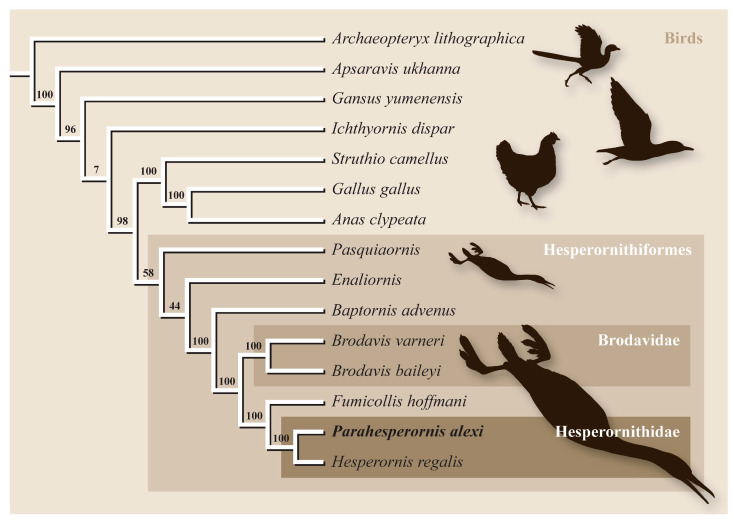
Phylogenetic tree from the analysis of Bell and Chiappe [16]. Numbers at nodes indicate bootstrap supports.

Bell and Chiappe [16] presented the first comprehensive phylogenetic analysis of the Hesperornithiformes, confirming a monophyletic Hesperornithidae containing *Parahesperornis* and *Hesperornis* united by 28 unambiguous synapomorphies [16] (Figure 3). This has since been supported by further analysis [12]. Within the Hesperornithiformes, Bell and Chiappe [16] recovered two monophyletic clades: the monogeneric Brodavidae, originally erected by Martin et al. [30] and containing four species (*B. americanus, B. baileyi, B. mongoliensis,* and *B. varneri)*, and the Hesperornithidae, which at the time of this study contains four genera: *Hesperornis* (containing eleven species) and the monospecific *Parahesperornis*, *Asiahesperornis*, and *Canadaga.* While the family Baptornithidae has been proposed [19], containing *Baptornis* and *Pasquiaornis* [29,65], phylogenetic analyses consistently reject this family as a monophyletic clade [12,16].

Bell and Chiappe [16] also identified *Fumicollis hoffmani* as the sister-taxon to the Hesperornithidae, a placement confirmed by subsequent analysis [12], making it the most-derived hesperornithiform outside of the Hesperornithidae. Disagreement exists over the placement of basal hesperornithiforms, including *Baptornis, Enaliornis,* and *Pasquiaornis.* For example, in studies in which both taxa are included, the basal-most hesperornithiform is recovered as either *Pasquiaornis* [16] or *Enaliornis* [12]. Given the highly fragmentary and problematic taxonomy of these two genera (see Methods below), this disagreement among studies is unsurprising. It should be noted that placement of these taxa is based on the coding of a very few characters. For example, the thoracic vertebrae of *Enaliornis* have been variously described as biconcave [3], amphicoelous [19], not fully heterocoelous [12], and fully heterocoelous [21,65,66]. This discrepancy is likely due to the high degree of weathering of these reworked specimens. A similar situation is involved with the vertebrae of *Pasquiaornis* [12,65]. Given the limited number of characters involved and the nature of the specimens, further work on both the taxonomy and phylogenetic positions of these taxa is warranted but may be limited to the discovery of more complete fossil material.

## 2. Materials and Methods

This study is based on a broad collection of Mesozoic bird fossils, primarily hesperornithiform, either studied directly in museum collections or from published descriptions in the scientific literature. As described above, the majority of hesperornithiform fossils consist of isolated elements. This can lead to difficulties in assigning different isolated elements to a single genus or species. Two such problematic hesperornithiform taxa are *Enaliornis*, with three species, and *Pasquiaornis*, with two species. Species designations for these genera are made primarily on the basis of size [3,21,29]. Both of these genera are described from large numbers of entirely isolated elements in bone bed deposits [3,29], making the assignment of different elements to the same genus problematic. For this reason, comparisons in the present study are primarily made between genera and not to the species level. *Brodavis* is one exception, as *B. varneri* consists of a multi-element holotype which varies substantially from the other species in the genus in a number of ways [30].

Anatomical nomenclature primarily follows Baumel and Witmer [67], with exceptions as noted in the text. Most specimens were measured using digital calipers, however where specimens could not be accessed measurements were collected from published photos using ImageJ (see Table S1 for details). Specimen numbers are used throughout to denote the source of specific observations. All specimens used in this study are held at federally recognized repositories and available for public access (see Table S2 for details). Institutional abbreviations are as follows: AMNH, American Museum of Natural History, New York, NY, USA; BMNH, The Natural History Museum, London, UK; FHSM, Sternberg Museum of Natural History, Fort Hays, KS, USA; FMNH, Field Museum of Natural History, Chicago, IL, USA; IZASK, Institute of Zoology, Ministry of Science, Almaty, Kazakhstan; KUVP, University of Kansas Museum of Natural History, Lawrence, KS, USA; LACM, Natural History Museum of Los Angeles County, Los Angeles, CA, USA; MCM, Mikasa City Museum, Mikasa, Japan; RSM, Royal Saskatchewan Museum, Regina, SK, Canada; NMC, Canada Museum of Nature, Ottawa, ON, Canada; NUVF—Nunavut Vertebrate Fossil collection (housed at the NMC); SDSM, Museum of Geology, South Dakota School of Mines and Technology, Rapid City, SD, USA; SMC, Sedgwick Museum of Earth Sciences, The University of Cambridge, Cambridge, UK; UNSM, University of Nebraska State Museum, Lincoln, NE, USA; USNM, Smithsonian National Museum of Natural History, Washington, DC, USA; YPM, Yale Peabody Museum, New Haven, CT, USA; YPM PU, Princeton University (collections now housed in the Yale Peabody Museum); ZIN PO, Paleornithological Collection of the Zoological Institute, Russian Academy of Sciences, St. Petersburg, Russia.

## 3. Results

### 3.1. Systematic Paleontology

Aves Linnaeus 1758
  Ornithuromorpha Chiappe 1995
  Ornithurae Haeckel 1866
  Hesperornithiformes Furbringer 1888
  Hesperornithidae *emend*.

The term “hesperornithidae” is generally attributed to Marsh [4], however in the referenced paper he used the term “hesperornidae”, not “hesperornithidae”. Marsh [5] later used the term “hesperornithidae” when describing *Lestornis crassipes* (later reassigned as *Hesperornis crassipes* [7]). A phylogenetic definition for the clade was later formalized by Clarke [2], who defined Hesperornithidae as a stem-based name containing all taxa more closely related to *Hesperornis regalis* than to *Baptornis advenus*. Phylogentic and taxonomic work by Bell and Chiappe [16,20] has since identified additional taxa intermediate to *B. advenus* and *H. regalis* not suitable for inclusion in the Hesperornithidae. Therefore, we are revising the name Hesperornithidae to a node-based definition containing all descendants of the common ancestor of *Hesperornis regalis* and *Parahesperornis alexi*. At the time of writing, that would include *Asiahesperornis bazhanovi* and *Canadaga arctica*, as well as *Parahesperornis alexi* and all species of *Hesperornis*, but would exclude the less derived *Fumicollis hoffmani.*

*Parahesperornis* Martin 1984

Type Species: *Parahesperornis alexi*

Included Species: *Parahesperornis alexi*

#### 3.1.1. Original Diagnosis

From Martin [40], “Skull mesokinetic; lacrimal more elongated dorsoventrally than in *Hesperornis*; nasal process of lacrimal more extended anteriorly than in *Hesperornis*; orbital process of quadrate very elongate; coracoid more elongate than in *Hesperornis*; femur more elongate and the proximal end less extended laterally than in *Hesperornis* and the tibiotarsus less compressed; tarsometatarsus with the outer trochlea only about one-quarter larger than the middle trochlea and both at about the same level distally (outer trochlea nearly twice the size of the inner trochlea and more distally situated in *Hesperornis*); crescent and peg articulations developed on the phalanges of the fourth toe of the foot as in other hesperornithids (absent in the baptornithids)” (p. 143).

#### 3.1.2. Amended Diagnosis

*Parahesperornis* is recognized by a unique suite of morphological features, including a small occipital condyle; narrow and deeply depressed recess in the *lamina parasphenoidalis*; sutures present on the basioccipitals; deep notch separating the zygomatic process and paraoccipital process; prominent paraoccipital processes; arched frontoparietal suture; interfrontal suture forms deep groove; reduced pneumaticity of the braincase; frontals flattened and very wide (width roughly 75% the length of the frontals); lacrimal elongate with long jugal process that terminates in a flattened face; caudomedial depression present on the quadrate; lateral crest of the quadrate restricted to the lateral margin; pterygoid triangular with flattened faces; premaxillae (width across the premaxillae at the nares is approximately 35% the pre-nares length); articulation of the angular to the dentary along a broad surface; surangulars convex laterally; expanded retroarticular process of the articular; anterior cervical vertebrae elongate while posterior cervical vertebrae become more compact; triangular coracoid with elongate neck; flat sternum with five costal processes; humerus reduced with only poorly defined condyles; elongate pelvis with reduced, circular acetabulum (acetabulum diameter approximately 10% ilium length); femur slightly waisted, with expanded trochanter and fibular condyle; head of femur extends proximally past trochanter; lateral condyle of femur extends only slightly distally past medial; elongate, triangular patella (distal mediolateral width approximately 50% of the proximodistal length); medial face of fibula with triangular depression; tibiotarsus with triangular cnemial expansion; tarsometatarsus with shingled metatarsals, proximal articular surface rhombic in proximal view, rounded intercotylar eminence, and trochlea IV extending slightly further distally than trochlea III; phalanges of pedal digit IV robust, with unevenly sized cotylae.

*Parahesperornis alexi* Martin 1984.

#### 3.1.3. Diagnosis

As for the genus.

#### 3.1.4. Holotype

KUVP 2287, an almost complete skeleton preserving a nearly complete, albeit crushed and disarticulated skull; 16 cervical, 6 thoracic, and 5 free caudal vertebrae; sternum; clavicle; coracoid; humeri; pelvis; femora; patellae; fibula; tibiotarsi; tarsometatarsi; 25 pedal phalanges; and impressions of skin. While histological analysis has not been conducted on this specimen, compound bone development is consistent with that of a fully-grown individual.

Type Locality & Geologic Setting. KUVP 2287 was discovered in 1894 by H. T. Martin in Graham County, Kansas, USA (KUVP locality number Gra-2). The specimen was collected from the Smoky Hill Member of the Niobrara Chalk Formation, which spans approximately five million years, from 82–87 Ma (late Coniacian to early Campanian) [68].

#### 3.1.5. Assigned Specimens

KUVP 24090, a partial skeleton preserving four isolated cervical vertebrae and at least 13 articulated posterior cervical and thoracic vertebrae, four articulated free caudal vertebrae, pygostyle, pelvis, sternum, coracoid, femora, patella, and tibiotarsi. KUVP 24090 was collected by Orville Bonner in 1981 from the Smoky Hill Member of the Niobrara Chalk Formation in Gove County, Kansas, USA (KUVP locality Gove-59). Compound bone development of KUVP 24090 is consistent with that of a fully-grown individual.

FHSM VP-17312, an isolated tarsometatarsus from the upper Smoky Hill Chalk in Logan County, Kansas, USA in the early 1990s by Jerome Bussen [26]. Compound bone development of FHSM VP-17312 is consistent with that of a fully-grown individual.

### 3.2. Description

#### 3.2.1. Cranial and Mandibular Elements

While cranial and mandibular material is rare among hesperornithiform fossils, *Parahesperornis alexi* KUVP 2287 preserves a nearly complete (albeit crushed) skull. Comparative material is largely limited to two fairly complete skulls (KUVP 71012, YPM 1206) of *Hesperornis,* which have been extensively described in the literature [7,8,56,57,58,69,70,71,72,73], and a number of specimens preserving fragmentary cranial elements. It should be noted that due to previous, but incorrect, assignment of KUVP 2287 to *Hesperornis gracilis*, some previous works describe the skull of KUVP 2287 under that name [58,59]. In addition to some fragmentary material with other *Hesperornis* specimens (FMNH 219, NMNH 4978, NMNH 6622, NMNH 13580, UNSM 10148, YPM 903, YPM 1207, YPM PU 18589), the only other skull material known from the Hesperornithiformes is the complete quadrate of *Potamornis skutchi* [49] (UCMP 73103), fragmentary portions of several unassociated skull elements assigned to *Pasquiaornis* [65], the caudal portion of the mandibular ramus of *Baptornis* (FMNH 395), and three fragmentary braincases assigned to *Enaliornis* [72]. The braincase of *Enaliornis* (SMC B54404) is exceptional in the lack of crushing of the specimen (see previous work [72] for a complete description of the braincase of *Enaliornis*). Very small fragments of the quadrate and frontal of *Baptornis* (AMNH 5101) were previously reported [19], however no such elements are included with the specimen today. Elzanowski [49] noted that the accession record of AMNH 5101 never listed cranial elements, and so concluded the specimen was incorrectly attributed to AMNH 5101. Likewise, a fragment of the bill was reported with *Baptornis* specimen KUVP 16112 [19], however the specimen did not include material identifiable as such when consulted for this study. Outside of the hesperornithiforms, cranial material in Mesozoic ornithuromorphs is largely crushed, making detailed comparisons of morphology difficult. The skull of *Ichthyornis* is known from a number of three-dimensional specimens [61], and so is one of the only other non-neornithine ornithuromorphs for which comparisons of many aspects of the cranium can be made.

**Figure 4:**
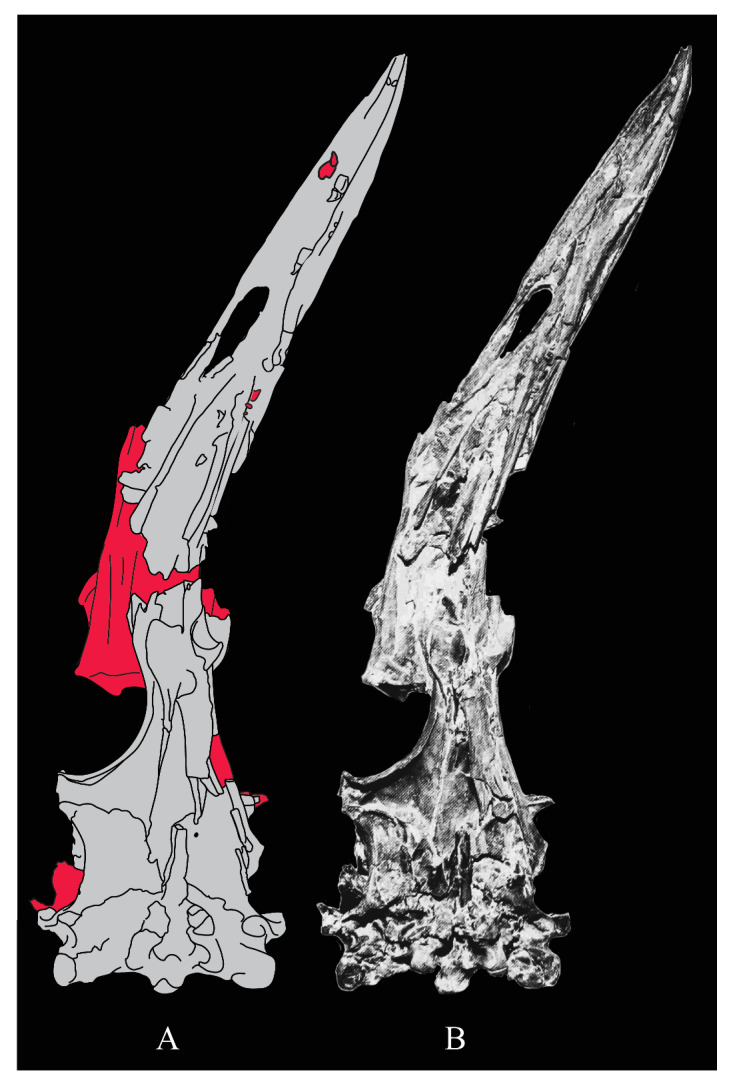
The articulated skull of KUVP 2287 before disarticulation [69]. Areas shaded red denote portions no longer present with the specimen.

**Figure 5:**
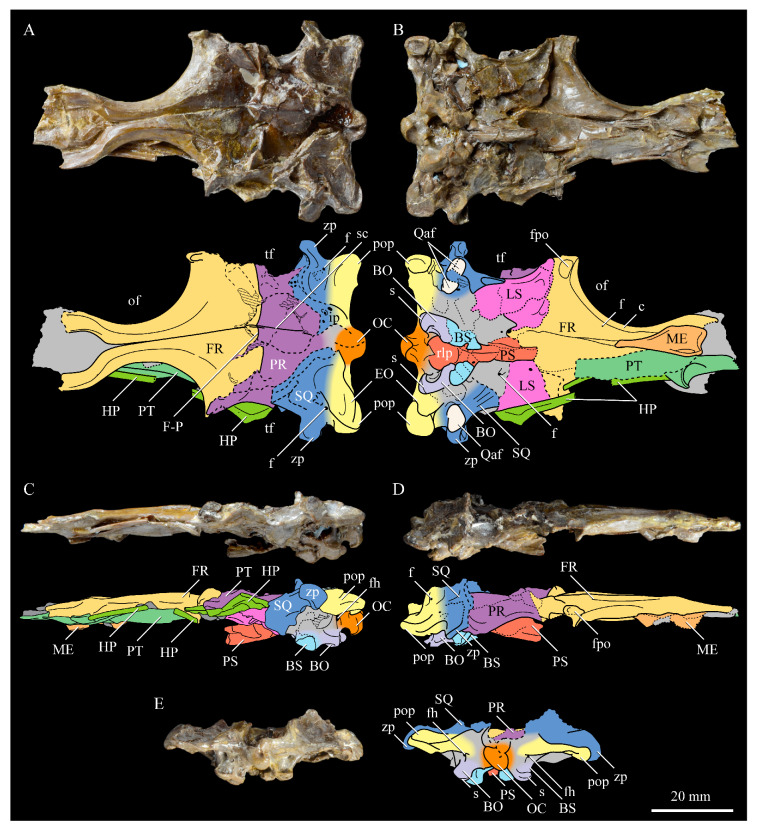
*Parahesperornis alexi* KUVP 2287, photographs (upper) and line drawings (lower) of the articulated braincase and frontals in dorsal (**A**), ventral (**B**), left lateral (**C**), right lateral (**D**), and caudal (**E**) views. Abbreviations: BO—basioccipital, BS—basisphenoid, c—crest (see text for discussion), EO—exoccipital, F-P—frontoparietal suture, f—foramen, fh—foramen for the hypoglossal nerve, fpo—facet for articulation of the prostorbital process of the parietal, FR—frontal, HP—hemipterygoid, ip—internal pneumaticity, ip—visible internal pneumaticity, LS—laterosphenoid, ME—mesethemoid, OC—occipital condyle, of—orbital fenestra, PR—parietal, pop—paraoccipital process, PS—parasphenoid rostrum, PT—palatine, Qaf—quadrate articular facet, rlp—recess in the *lamina parasphenoidalis,* sc—sagittal crest, s—suture, SQ—squamosal, tf—temporal fenestra, zp—zygomatic process.

**Figure 6:**
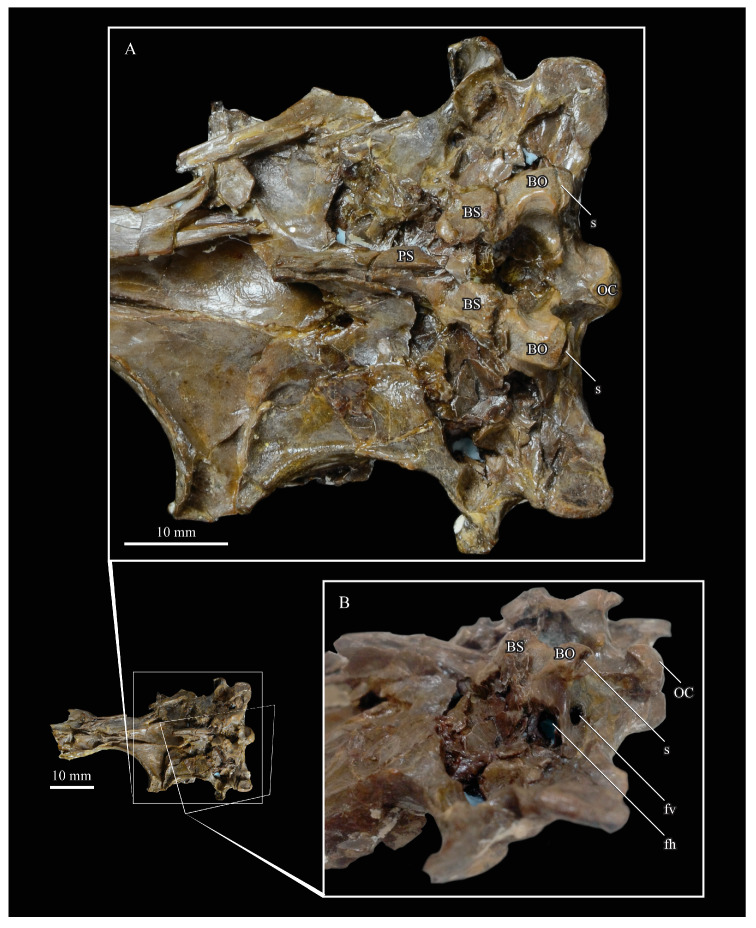
*Parahesperornis alexi* KUVP 2287, enlargement of the dorsal surface of the braincase in dorsal (**A**) and in laterodorsal view (**B**). Abbreviations: BO—basioccipital, BS—basisphenoid, fh—hypoglossal foramen, fv—vagus foramen, OC—occipital condyle, s—suture.

**Figure 7:**
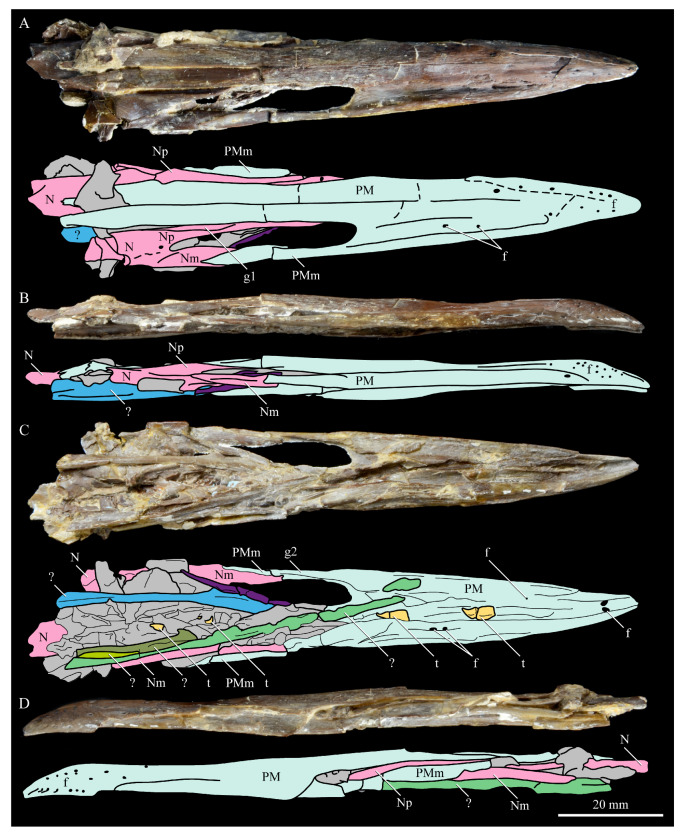
*Parahesperornis alexi* KUVP 2287, premaxillary region photographs (upper) and line drawings (lower) in dorsal (**A**), right lateral (**B**), ventral (**C**), and left lateral (**D**) views. Abbreviations: f—foramen, g1—groove on the premaxillary process of the nasal, g2—groove on the maxillary process of the premaxilla, N—nasal, Nm—maxillary process of the nasal, Np—premaxillary process of the nasal, PM—premaxilla, PMm—maxillary process of the premaxilla, t—tooth, ?—unidentified palatal bones, see text for more detail.

**Figure 8:**
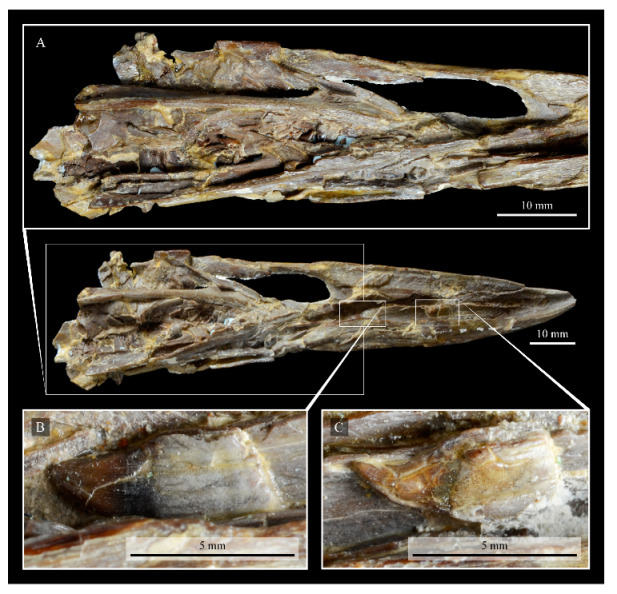
*Parahesperornis alexi* KUVP 2287, details of the ventral surface of the premaxilla showing the region preserving unidentified palatal elements (**A**) and individual teeth (**B**,**C**).

The skull of KUVP 2287 was originally preserved partially disarticulated. The majority of the cranium and upper jaw were articulated (Figure 4), while the lower jaws, quadrates, pterygoids, palatines, and lacrimals were preserved as isolated elements. At some point, the cranium and upper jaws were disarticulated into two pieces, one including the braincase and frontals (Figure 5 and Figure 6) and the second piece consisting of the premaxillae, nasals, and a number of palatal elements (Figure 7 and Figure 8). This is important to note, as during this process some material from the specimen was lost (see Figure 4). Possible identifications for these missing pieces are discussed below.

**Figure 9:**
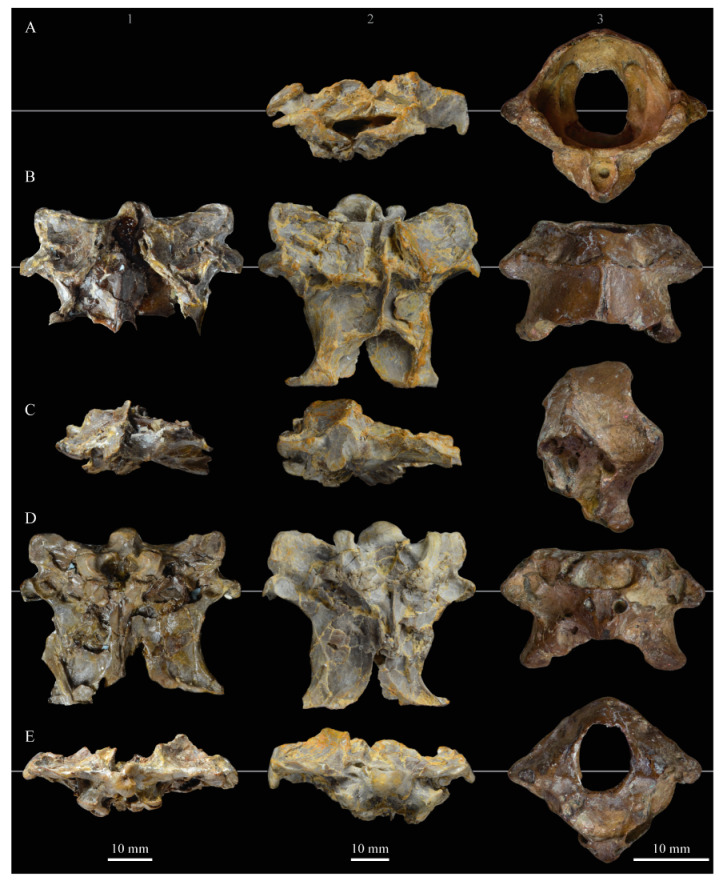
Comparison of the braincases of *Parahesperornis* KUVP 2287 (1), *Hesperornis* KUVP 71012 (2), and *Enaliornis* SMC B54404 (3) in rostral (**A**), dorsal (**B**), lateral (**C**), ventral (**D**), and caudal (**E**) views. Specimens are scaled to be of similar widths at the zygomatic processes. The articulated frontals have been digitally removed for 1.

**Braincase.** The braincase of KUVP 2287 is highly crushed dorsoventrally and articulated to the frontals (Figure 5 and Figure 6). For comparisons, braincases of *Hesperornis* (KUVP 71012) and *Enaliornis* (SMC B54404) were examined (Figure 9). In ventral view (Figure 5B), the occipital region of KUVP 2287 is fairly clear. The occipital condyle is broken into halves and is slightly hook-shaped, with the rounded surface curving over the somewhat indented dorsal base of the condyle. Despite the breakage, the occipital condyle of *Parahesperornis* appears to be much smaller proportionally than that of *Hesperornis* (Figure 9). The occipital condyle of *Enaliornis* is not preserved.

Ventrally, rostral to the occipital condyle is a deep, round recess in the *lamina parasphenoidalis* (rlp in Figure 5). This recess appears to be narrower and more deeply excavated in KUVP 2287 than in *Hesperornis*, however this may be enhanced by crushing. In *Enaliornis* this recess is prominent and mediolaterally elongate, taking up a larger portion of the ventral skull than in either *Parahesperornis* or *Hesperornis*, perhaps indicating the degree of crushing in both KUVP 2287 and KUVP 71012.

In *Parahesperornis* the recess of the *lamina parasphenoidalis* is defined by the basioccipital and basisphenoid (BO and BS, respectively, in Figure 5); the latter bones wrap around the recess and nearly meet at the base of the parasphenoid rostrum (PS in Figure 5). The basioccipitals of *Parahesperornis* are roughly kidney-shaped, with what appear to be prominent sutures on the caudal ends, the right of which is somewhat open (s in Figure 5 and Figure 6). In *Hesperornis* the basioccipitals are more elongate with less curvature and lack any visible sutures. These elements are not preserved in *Enaliornis.*

The basisphenoids of *Parahesperornis* are both somewhat crushed but appear to overlap the basioccipitals, with a flattened face that angles toward the parasphenoid rostrum, as in *Hesperornis*. There appears to be a suture present between the basioccipital and basisphenoid of *Parahesperornis*. A similar suture is present and more obvious in *Hesperornis*. In *Enaliornis,* the area rostral to the recess in the *lamina parasphenoidalis* is very different, preserving a distinct pyramidal projection with two large fossae on the lateral and medial margins, tentatively identified as the *canalis orbitalis* by Elzanowski and Galton [72].

The parasphenoid rostrum of *Parahesperornis* extends rostrally as a straight, paired bone with a smoothly rounded ventral surface (PS in Figure 5). Lucas [13] noted that the parasphenoid rostrum extended to the mesethemoid in KUVP 2287, however today it is broken considerably caudal to the mesethemoid. In *Hesperornis* the ventral surface of parasphenoid rostrum is projected and forms a crest. The parsphenoid rostrum is not preserved in *Enaliornis.*

The basioccipitals are propped up on struts of bone, into which two foramina are visible piercing the base of the strut (Figure 5 and Figure 6). While the exact identity of these foramina is uncertain, it is likely that the smaller, more caudal foramen corresponds to the foramen for the exit of the hypoglossal nerve (XII) (fh in Figure 5 and Figure 6) while the larger, laterally facing one corresponds to the foramen for the vagus nerve (X) (fv in Figure 6). This is unlike *Hesperornis*, where three small foramina are seemingly associated with the hypoglossal nerve, while *Parahesperornis* has only one. In *Enaliornis* these foramina are present at the caudomedial and -lateral borders of the recess in the *lamina parsphenoidalis* (Figure 9).

Lateral to these foramina the exoccipital extends sideways, forming a prominent rounded paraoccipital process with a flattened ventral surface (pop in Figure 5; sometimes referred to as the exoccipital process [67]). This process is separated from the more rostrally-located zygomatic process (zp in Figure 5) by a deep indentation in the lateral margin. This indent is much less dramatic in *Hesperornis*, where the exoccipital process and prootic appear as a more continuous rectangular shape in ventral view. The indentation is lacking entirely in *Enaliornis,* where the paraoccipital processes are much reduced and appear as very slight protuberances in dorsal view (Figure 9).

The zygomatic process of *Parahesperornis* extends laterally and slightly rostrally from the edge of the dorsal cranium and has a flattened face that has been crushed under ventrally on both sides. This face is better preserved in *Hesperornis* KUVP 71012, where it is oval shaped with a slightly domed surface and appears to form a slight hook, however discrepancies between the left and right sides make it unclear how much of that shape is due to crushing (Figure 9). The zygomatic processes of both *Parahesperornis* and *Hesperornis* are much reduced as compared to those of *Enaliornis*, where they form broad triangular processes directed more rostrally than in hesperornithids (Figure 9).

Dorsally, the area of the squamosals and exoccipitals medial to the zygomatic and paraoccipital process is developed as a broad, shallowly depressed face that is roughly rectangular. A few small foramina are preserved near the lateral indentation in the margin between the zygomatic and paraoccipital processes. In contrast to *Parahesperornis*, a single foramen is observed in *Hesperornis*; however, the region is obscured by crushing in KUVP 71012, the only skull available for study where this feature is observed. In *Hesperornis* this area also forms a broad depression; however, the shape approaches a square in *Hesperornis* as opposed to the rectangle in *Parahesperornis*. In *Enaliornis* this area is smaller and flattened as compared to the depressed surface in hesperornithids.

Ventrally, deep facets are developed medial to the zygomatic processes for the articulation with the otic processes of the quadrate (Qaf in Figure 5). This facet is better preserved on the left side of the skull and is like that seen in *Hesperornis.* In *Enaliornis* the articulation for the quadrate is less defined, and forms a shallower depression divided into two similarly sized facets for articulation with the squamosal and otic heads of the quadrate, separated by the dorsal pneumatic foramen (Figure 9). This foramen is not observed in the facet in KUVP 2287 or KVUP 71012.

In ventral view, the braincase of *Parahesperornis* makes a sweeping curve at the margin of the temporal fossa that ends in a laterally projecting terminus where the braincase articulates with the frontals. This terminus does not appear to form a well-developed postorbital process (as in *Ichthyornis* [61]), however it is possible breakage has obscured this. The margin of the temporal fossa echoes that of the orbital margin of the frontals but is slightly shorter and indented to a lesser degree. It is unclear where the boundary between the parietals and the laterosphenoid occurs. As such our identifications of the boundaries of these bones (PR and LS in Figure 5) should be considered tentative. *Hesperornis* has a much more exaggerated lateral projection of the postorbital process than *Parahesperornis*, while that of *Enaliornis* is reduced (Figure 9). The ventral walls of the braincase leading up to this margin are smooth but extremely crushed in *Parahesperornis*. In *Hesperornis,* they slope slightly ventrally to meet below the parasphenoid rostrum. The breakage pattern in KUVP 2287 might suggest that the walls of the braincase were not fused where they met at the midline, however this cannot be confirmed.

While crushing might obscure some details of the exact margins of the temporal fossa, it appears to be mostly open and only partially enclosed by the zygomatic process caudally and the postorbital process rostrally. The temporal fossa of *Hesperornis* is similar, while that of *Enaliornis* is much shorter caudorostrally. The structure of the temporal fossa is intermediate between most modern birds, where it is much reduced and only slightly enclosed by bone, and *Ichthyornis*, where the temporal fossa is almost completely enclosed by bone, more similar to the primitive dinosaurian condition [61]. The temporal fossa in some Neornithes, such as the extinct (Mio-Pliocene) flightless alcids Mancallinae, appear to be most similar to that of the hesperornithids [74].

In dorsal view the caudal portion of the surface of the braincase of *Parahesperornis* KUVP 2287 has split along the sagittal crest (sc in Figure 5), obscuring the foramen magnum. While it is not possible to describe the sagittal crest in *Parahesperornis,* in *Hesperornis* it is a large, sharp crest tightly sutured along its length (Figure 9). As the point of attachment for the deep fascia of the neck muscles^67^, the robust sagittal crest indicates the significance of these muscles in stabilizing the neck while diving. In *Enaliornis* the sagittal crest is only faintly developed caudally, between the squamosals, and appears to become less sutured rostrally, with the rostral extent partially open.

**Figure 10:**
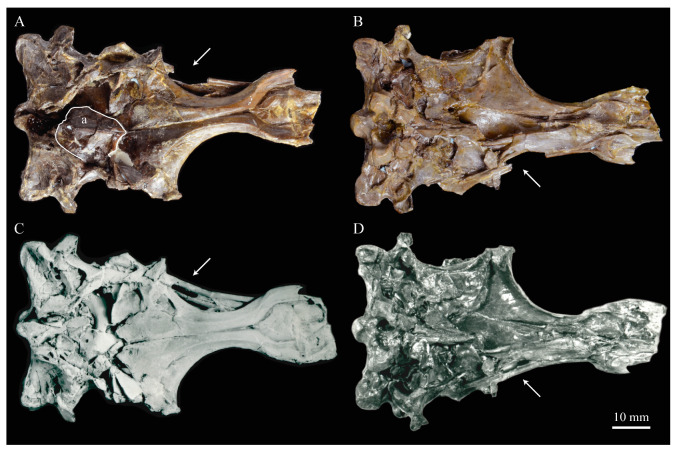
Braincase and orbital region of *Parahesperornis alexi* KUVP 2287 after (**A**,**B**) and before (**C**,**D**) preparation in dorsal (**A**,**C**) and ventral (**B**,**D**) views. Arrows indicate a missing section of the hemipterygoid, while the outline in A shows a small piece of the parietals apparently added.

**Figure 11:**
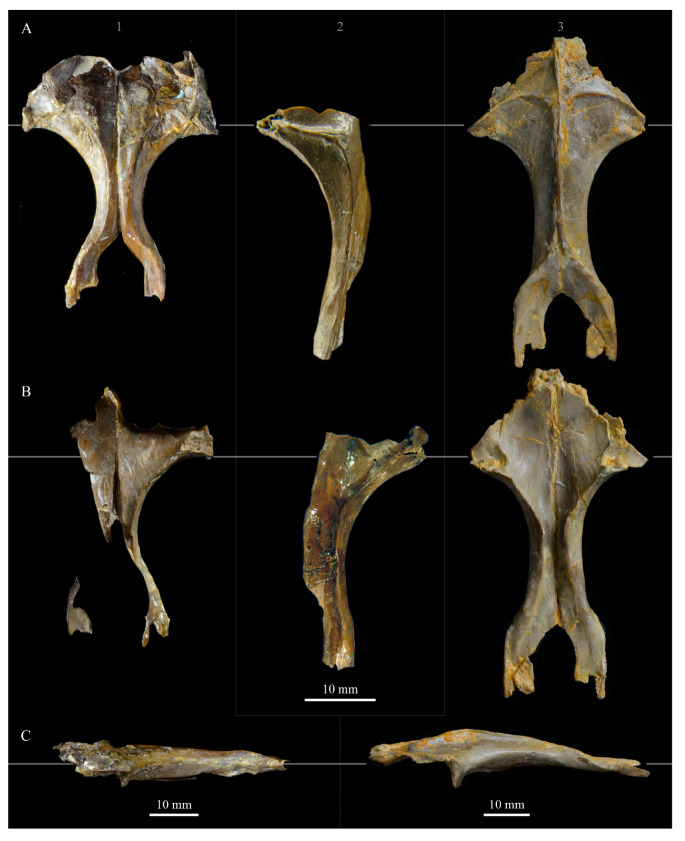
Comparison of the frontals of *Parahesperornis* KUVP 2287 (1), *Hesperornis* KUVP 71012 (2), and *Pasquiaornis* RSM P2995.4 [65] (3) in dorsal (**A**), ventral (**B**), and right lateral (**C**) views. Specimens are scaled to be of similar size across the right frontal and aligned at the caudal margin of the orbit. Extraneous bones and fragments have been digitally removed from 1.

Rostral to the squamosal area on the dorsal side, the braincase of KUVP 2287 is heavily crushed, however a small piece in the center of the crushed area preserves a section of the parietals with the sagittal crest and a piece that appears to contain the frontoparietal suture (F-P in Figure 5). It should be noted that this piece may have been positioned during preparation, as undated photographs show the specimen without this piece (Figure 10). The frontoparietal suture is better preserved in *Hesperornis*, where it runs in a broad transversal arc and forms a cross where it intersects the sagittal crest (Figure 11). In *Hesperornis* the frontoparietal contact appears to be fully sutured. The small portions of the preserved frontoparietal suture in *Parahesperornis* KUVP 2287 agree with this morphology.

This observation contradicts Martin’s [40] interpretation of the frontals and parietals as meeting in a moveable joint, giving *Parahesperornis* a mesokinetic cranium, while the juncture in *Hesperornis* was fused and immobile. Later work by Buhler et al. [57] described the frontoparietal juncture of *Parahesperornis* as consisting of a posterior ridge on the frontals that fit into an anterior groove on the parietals; however, due to the degree of crushing in KUVP 2287 this cannot be confirmed. Rather, the small portion of the frontoparietal suture preserved where it intersects the sagittal crest forms a crest and appears to be sutured.

In *Enaliornis,* the three known braincases are broken along what has been interpreted to be the frontoparietal suture [21]. If correct, then the frontoparietal suture was nearly linear in *Enaliornis*, not arced as in hesperornithids. This consistent breakage pattern was also used to infer an open frontoparietal suture in *Enaliornis* [21]. One specimen assigned to *Pasquiaornis* (RSM P2995.4) preserves a portion of the frontoparietal suture, which does not appear to be open [65]. The suture forms a low, thin crest, that is fairly straight and angles caudally from the juncture with the sagittal crest. In all, despite apparent differences in the trajectories of the frontoparietal suture, with the possible exception of *Enaliornis*, the frontals and parietals of hesperornithiforms appeared to have been sutured to each other.

The braincases of *Parahesperornis* and *Hesperornis* show much less pneumaticity than *Enaliornis*. In *Enaliornis* the braincase is highly pneumatic, with multiple foramina on the caudal end in the occipital region [72]. While the crushing of the braincases of *Parahesperornis* KUVP 2287 and *Hesperornis* KUVP 71012 may have obscured some foramina in the rostral portions, the caudal braincase is fairly well preserved, and it is doubtful that either skull would have approached the level of pneumaticity seen in *Enaliornis*. In KUVP 2287, internal views along the broken sagittal crest show some pneumatization of the interior of the bone (ip in Figure 5). However, there is no evidence of internal pneumaticity in the broken edges of *Hesperornis* KUVP 71012.

**Frontals.** The frontals of *Parahesperornis* KUVP 2287 are preserved somewhat articulated with and flattened back upon the braincase (Figure 5). For comparison, the frontals of *Hesperornis* (KUVP 71012, YPM 1206) and *Pasquiaornis* (RSM P2995.4 [65]) were examined (Figure 11). In dorsal view, the frontals of KUVP 2287 meet at the interfrontal suture, which forms a slight groove. To either side of this groove the frontals are flattened, with an arced, central crest that parallels the margin of the orbit, running along the length of the dorsal surface. Such morphology is very different from that of the frontals of *Hesperornis* and *Pasquiaornis,* where the interfrontal suture forms a longitudinal crest, continuous with the sagittal crest.

The central region of the sutured frontals is shortened in *Parahesperornis* as compared to *Hesperornis*. This gives the mediocaudal margin of the orbit, as formed by the lateral edge of the frontals, a more circular shape in *Parahesperornis*, whereas in *Hesperornis* the orbit is elongate and much more shallowly curved. It is difficult to compare the shape of the incomplete caudal orbit in *Pasquiaornis*; however, it appears to be intermediate between the morphologies of *Hesperornis* and *Parahesperornis.* In relative terms, *Parahesperornis* has a much broader skull than *Hesperornis* at the level of the orbits. The frontals of *Hesperornis* and *Parahesperornis* have similar widths, despite *Parahesperornis* being a much smaller animal overall. *Pasquiaornis* appears proportionally narrower as well, more like *Hesperornis*.

The frontals of *Parahesperornis* appear to be tightly sutured along the majority of their length, however at the rostral-most end they become slightly separated from one another before the left and right frontals diverge and define a dramatic U-shaped rostral terminus (Figure 5A). This U-shaped rostral end of the frontals is also seen in *Hesperornis* and *Pasquiaornis*, however the U-shape is narrower in the latter (Figure 11). This contrasts with *Ichthyornis*, where the frontals angle away from each other slightly, forming a Y-shape [61]. In lateral view, the frontals of *Parahesperornis* are fairly flat, as in *Pasquiaornis*, however in *Hesperornis* they are strongly domed caudally (Figure 11C).

In *Parahesperornis,* the dorsal margin of the frontals forming the upper orbit slope toward the orbit fairly steeply along their entire length, forming an angle dorsally where they flatten out medially before meeting at the interfrontal suture. *Pasquiaornis* resembles *Parahesperornis* in the angle of the flattened surface, however in *Hesperornis* the angle is entirely lacking, and the sides of the frontals slope continuously from the juncture of the interfrontal and sagittal crests with the frontoparietal suture. Lucas [13] described *Parahesperornis* KUVP 2287 as having depressions for supraorbital glands (*fossa glandula nasales*). In modern birds the lateral edges of the frontal dorsal to the orbit are often broad with a well-developed depression for the supraorbital gland. The morphologies seen in hesperornithiforms cast doubt about the interpretation of supraorbital glands, which if present, would have been rather small.

In ventral view, the orbital margin of the frontals forms a sharp lip or crest in *Parahesperornis* (c in Figure 5), as in *Pasquiaornis*, while in *Hesperornis* the frontals wrap over and nearly form a tunnel along the ventral orbital margin (Figure 11). At its caudal-most end, this lip remains a consistent height in *Parahesperornis*, while in *Hesperornis* it forms an exaggerated ventrally directed projection, best seen in lateral view (Figure 11C). At the caudal-most edge of the orbital margin the frontals narrow to a triangular projection with a facet for the postorbital process of the parietal (fpo in Figure 5). This facet is much more strongly developed in *Hesperornis*, where it is separated from the medial region of the frontals by a ridge that is only faintly visible in *Parahesperornis*.

**Figure 12:**
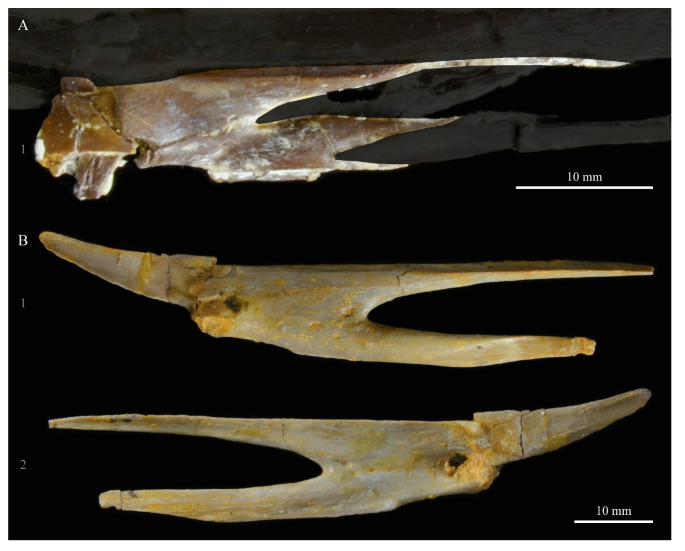
Comparison of the nasals of *Parahesperornis* KUVP 2287 (**A**) in lateral view (1) and *Hesperornis* KUVP 71012 (**B**) in lateral (1) and medial (2) views. Specimens are scaled to be of similar size.

**Nasals.** The nasals of *Parahesperornis* KUVP 2287 are poorly preserved. Both the left and right are preserved crushed against the premaxillae, with the right more easily observed (Figure 7). For comparison, the nasals of *Hesperornis* (KUVP 71012, YPM 1206), the only other hesperornithiform from which nasals have been reported, were examined (Figure 12). The premaxillary and maxillary processes of the nasal of *Parahesperornis* (Np and Nm, respectively, in Figure 7) extend cranially in a similar orientation and run parallel to each other along their length, as in *Hesperornis*. A small foramen is present on the main body of the nasal at the juncture of these processes in both *Parahesperornis* and *Hesperornis* (Figure 12).

The dorsal surface of the premaxillary process of the nasal of *Parahesperornis* appears to have a broad, faint groove caudally, near the juncture with the maxillary processes, that flattens rostrally (g1 in Figure 7). In *Hesperornis*, the dorsal surface of the premaxillary process is flattened along its length with a similar groove along the dorsal-most edge that does not extend as far caudally as in *Parahesperornis*. The articulation between the maxillary processes of the nasal and premaxilla is clearly visible; the maxillary process of the premaxilla thins dramatically to a very thin sheet of bone that overlays the lateral face of the maxillary process of the nasal for a few centimeters, forming what Gingerich [58] termed a subnarial bar. Details of how this subnarial bar articulated with the maxilla are not well preserved in KUVP 2287. However, the ventral surface of the maxillary process of the right premaxilla appears to be flattened with a very faint caudal groove (g2 in Figure 7). Previously, Gingerich [58] identified the subnarial bar as sliding into a prominent groove developed on the dorsal margin of the maxilla from observations of *Hesperornis* YPM 1206. This is also seen in *Hesperornis* KUVP 71012, but a maxilla is not preserved (or is not complete enough to be identified) with KUVP 2287.

**Figure 13:**
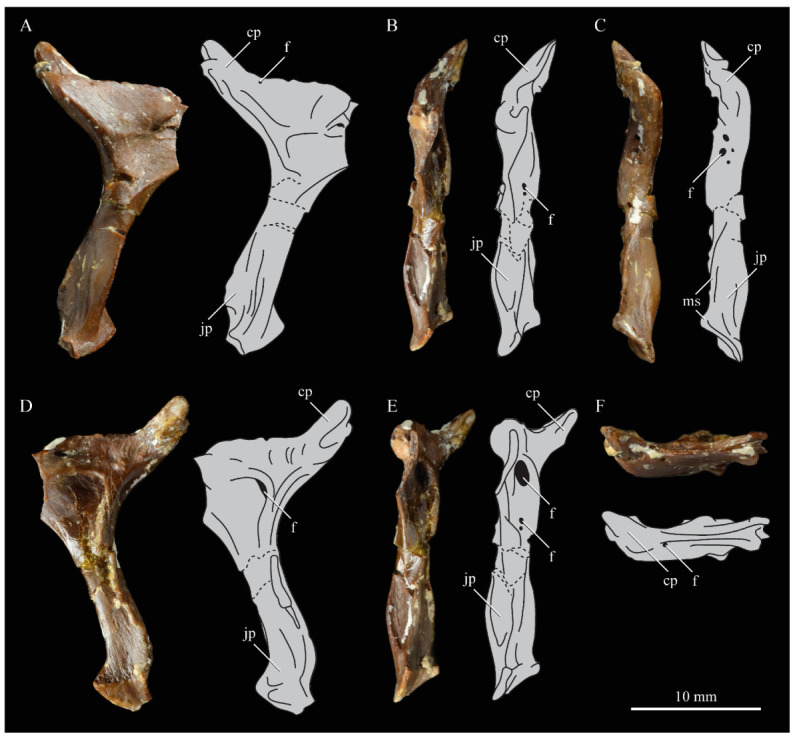
*Parahesperornis alexi* KUVP 2287 photographs (left) and line drawings (right) of the right lacrimal in lateral (**A**), cranial (**B**), caudal (**C**), medial (**D**), craniomedial (**E**), and dorsal (**F**) views. Abbreviations: cp—caudal process, f—foramen, jp—jugal process, ms—muscle scar.

**Figure 14:**
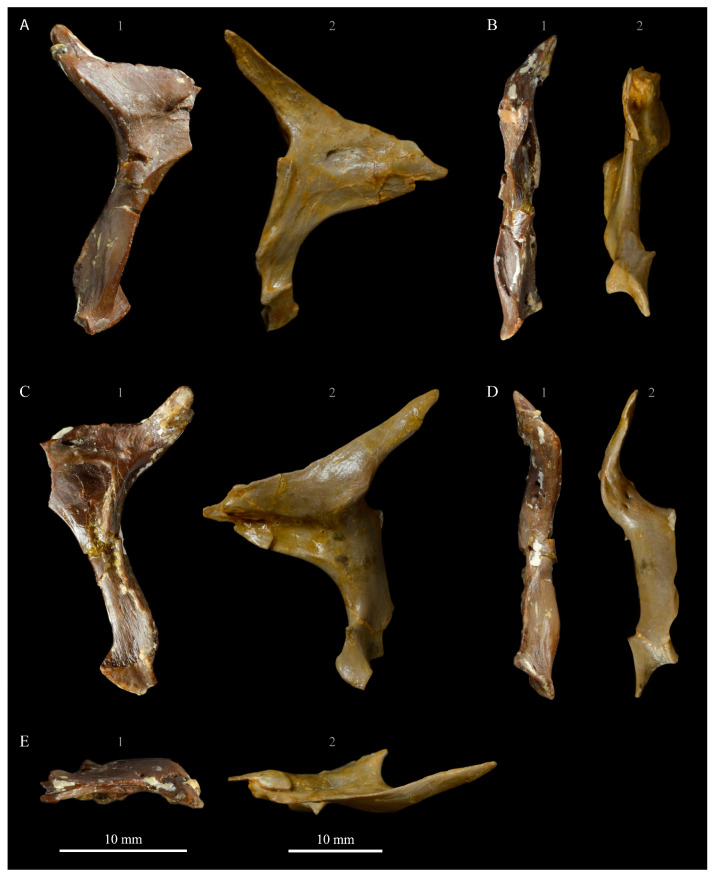
Comparison of the lacrimals of *Parahesperornis* KUVP 2287 (1) and *Hesperornis* KUVP 71012 (2) in medial (**A**), cranial (**B**), lateral (**C**), caudal (**D**), and dorsal (**E**) views. Specimens are scaled to be of similar dorsoventral heights.

**Lacrimals**. Portions of both lacrimals are preserved with *Parahesperornis* KUVP 2287. The right lacrimal is nearly complete but lacks the cranial process (Figure 13), while the left consists of only a small fragment of the central region. For comparison, the lacrimals of *Hesperornis* KUVP 71012 were examined (Figure 14). The lacrimal of *Parahesperornis* is roughly T-shaped, with the caudal process extending dorsocaudally and the jugal process extending ventrally and slightly caudally.

The lacrimal of *Parahesperornis* differs from that of *Hesperornis* in several respects. Overall, the lacrimal of *Parahesperornis* is much more elongate than that of *Hesperornis*, with a relatively narrow body and proportionally longer jugal process. The end of the jugal process in *Parahesperornis* terminates in an angled, flattened face with an upturned lip around the margin for articulation with the jugal. However, the jugal process of *Hesperornis* terminates in a pointed foot. At the base of the caudal margin of the jugal process, *Parahesperornis* has a muscle scar that is absent in *Hesperornis*. Additionally, in caudal view, the face of the jugal process of *Parahesperornis* is highly irregular, with a deep ovoid depression along its surface. In *Hesperornis* the corresponding area is narrow and rounded, with only a slight depression centered at the distal end.

In lateral view, the side of the lacrimal of *Parahesperornis* has an ovoid depression that appears to shallow rostrally, such that it would not have extended beyond the broken edge of the cranial process. This region is more deeply excavated in *Hesperornis*, where it forms a groove that parallels the dorsal margin of the body of the lacrimal. Also in lateral view, the caudal process forms a dorsal curve in *Parahesperornis* but angles dorsally in a straight line in *Hesperornis.*

Witmer [75] established that the lacrimals of both *Hesperornis* and *Parahesperornis* are pneumatic. In *Parahesperornis* a large, funnel-like opening is present on the caudal margin of the depression in the lateral face of the lacrimal (best seen in craniomedial view, Figure 13E). In *Hesperornis*, the external evidence of this pneumaticity is limited to a small foramen located in the middle of the medial face of the lacrimal.

The overall shape of the lacrimal, and in particular the elongation of the jugal process, in *Parahesperornis* is similar to that of other Mesozoic ornithuromorphs (e.g., *Ichthyornis* [61] and *Dingavis* [76]), which share an overall T-shaped morphology with *Archeopteryx* [77], reflecting the ancestral state in birds. This highlights the more compact lacrimal of *Hesperornis*, with a shortened jugal process, as an autapomorphy among Mesozoic birds.

**Figure 15:**
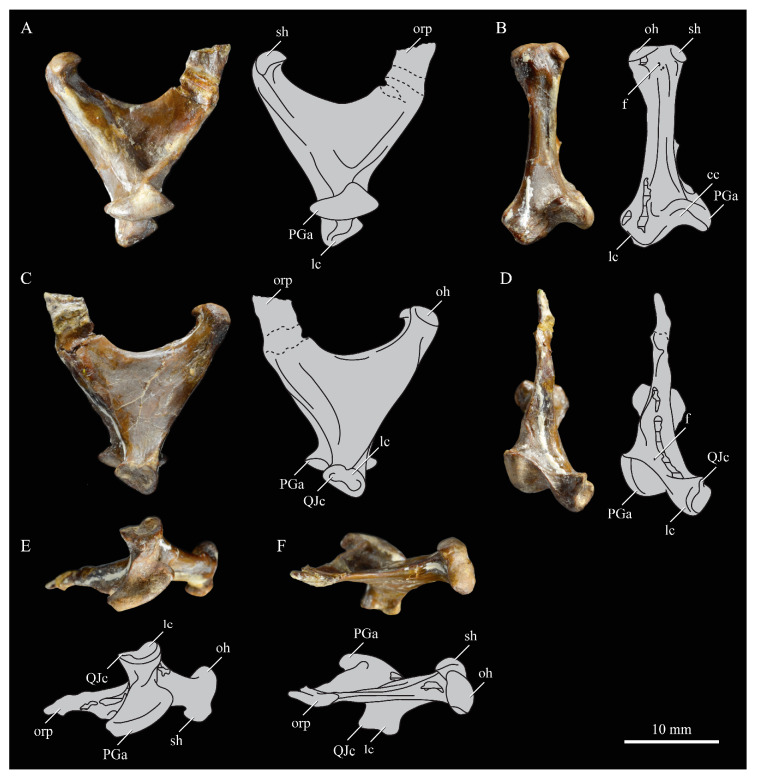
*Parahesperornis alexi* KUVP 2287, photographs (left) and line drawings (right) of the left quadrate in medial (**A**), caudal (**B**), lateral (**C**), rostral (**D**), ventral (**E**), and dorsal (**F**) views. Abbreviations: cc—caudal condyle, f—foramen, lc—lateral condyle, oc—otic head, or—orbital process, PGa—pterygoid condyle, QJc—quadratojugal cotyla, sc—squamosal head.

**Figure 16:**
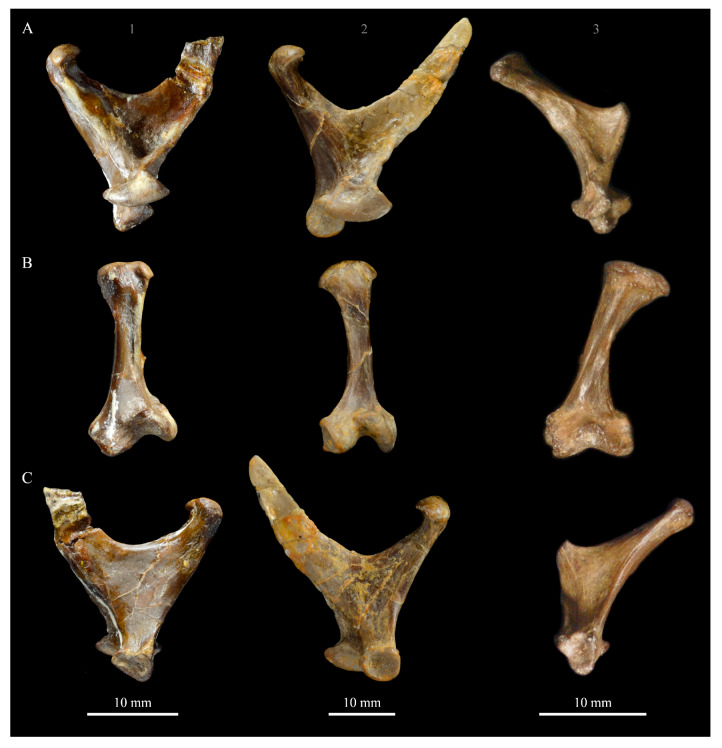
Comparison of the quadrates of *Parahesperornis* KUVP 2287 (1), *Hesperornis* KUVP 71012 (2), and *Potamornis* UCMP 73013 (3) in medial (**A**), caudal (**B**), and lateral (**C**) views. Specimens are scaled to be of similar height at the caudal end and aligned at the top of the head.

**Quadrate.** *Parahesperornis* KUVP 2287 preserves the complete left quadrate (Figure 15) and fragments of the otic process and dorsal articular region of the right quadrate. In addition to *Hesperornis* (KUVP 71012), a well-preserved quadrate is also known for *Potamornis* [49] (UCMP 73013) (Figure 16), and two poorly preserved quadrates have been reported for *Pasquiaornis* (RSM P2988.25, RSM P2831.52) [65]. As discussed above, a partial quadrate previously described [19] as belonging to *Baptornis* (AMNH 5101) was likely a mistaken attribution [49]. Anatomical terminology for the discussion of the quadrate comes from Elzanowski [49], who expanded upon the terminology of Baumel and Witmer [67]. In general, the quadrates of hesperornithiforms are similar, with some minor differences in proportion and details of morphology, and much like those of modern birds and *Ichthyornis*.

The otic process of the quadrate is poorly divided in *Parahesperornis*, as in other hesperornithids and modern paleognathes, but unlike the better defined otic and squamosal head in *Ichthyornis* and many modern birds. The otic head is slightly smaller than the squamosal head, while in *Hesperornis* and *Potamornis* the otic head is much smaller than the squamosal. While the quadrate of *Enaliornis* is not known, the division of the quadrate’s articular facet on the squamosal into visible medial and lateral cotylae indicate the otic and squamosal heads of the quadrate of *Enaliornis* may have been better divided.

In caudal view, the intercapitular incision wraps down onto the caudal face of the otic process and around the ventral margin of the otic head forming a caudomedial depression. This depression is much better developed in *Parahesperornis* than in *Potamornis*, while in *Hesperornis* the depression is absent. In medial view the otic process hooks backward over the neck and projects rostrally, as in *Hesperornis* and unlike *Potamornis,* where it is not hooked at all. In both *Parahesperornis* and *Hesperornis,* a small pocket is present beneath the lip of the head on the rostral surface of the neck. The neck is more robust in *Parahesperornis* and *Potamornis,* where it narrows very little before the otic process, while in *Hesperornis* the neck is more restricted.

The dorsal margin of the quadrate, between the otic process and the orbital process, is broad and u-shaped in *Parahesperornis*, while in *Hesperornis* it is slightly deeper (resulting from the longer neck of the otic process in *Hesperornis*) and more v-shaped. The orbital process of *Parahesperornis* KUVP 2287 lacks the anterior end, but the preserved portion is very thin mediolaterally and tapers anteriorly, as in *Hesperornis.* Elzanowksi reported the orbital process of *Potamornis* as only missing the anterior-most end. If this is true, then the orbital process was dramatically reduced in *Potamornis* as compared to hesperornithids. The thin, flattened orbital process plays a role in the mobility of the quadrate, as that is where the m. protractor pterygoidei et quadrati, which pulls the quadrate dorsorostrally, inserts, and the m. pseudotemporalis profundus, which adducts the mandible [78], originates [57].

In medial view, the body of the quadrate of *Parahesperornis* just above the medial condyle is deeply excavated in a triangular pocket, as in *Pasquiaornis*. *Potamornis* has a shallow depression in this area, while the corresponding region in *Hesperornis* is only faintly depressed. In lateral view the body of the quadrate of *Parahesperornis* is fairly featureless and flat, while in *Hesperornis* and *Potamornis* the central region is slightly depressed. Also in *Hesperornis* and *Potamornis,* the neck is slightly twisted, such that the lateral crest crosses the lateral face just below the otic process, while in *Parahesperornis* the lateral crest is restricted to the lateral margin.

The caudal condyle on the mandibular process of *Parahesperornis* is small and thin, located to the medial side of the distocaudal surface. In *Hesperornis* this condyle is more robust, present as a thick, triangular projection that occupies most of the caudal surface of the pterygoid condyle. *Potamornis* has a small caudal condyle, similar in size to that of *Parahesperornis,* but developed as a hook projecting caudodorsally, unlike in other hesperornithiforms.

The pterygoid condyle is very well-developed and like that of modern birds. In *Parahesperornis* and *Potamornis* the pterygoid condyle is broad and triangular, while in *Hesperornis* it is oval with a slightly concave surface. The medial condyle of *Parahesperornis, Potamornis,* and *Pasquiaornis* is elongate and narrower at the caudal end than at the rostral end, with a slightly convex dorsal margin. That of *Hesperornis* is more uniform in width with much deeper curvature along the dorsal margin.

The lateral condyle of *Parahesperornis* is narrower rostrocaudally than the pterygoid condyle, with an oval quadratojugal cotyla facing laterally and slightly rostrally. The margins of the cotyla are slightly pinched in toward the rostral end, creating a rostral projection that may correspond to the quadratojugal buttress identified in *Potamornis* by Elzanowski [49]. The quadratojugal cotyla is nearly circular in *Hesperornis*, where it faces directly laterally. That of *Potamornis* is oval in shape, however the long axis of the cotyla is oriented dorsoventrally, while in *Parahesperornis* the long axis is oriented rostrodorsally to caudoventrally. The pneumaticity of the quadrate in hesperornithids was discussed by Witmer [75] and Elzanowski [49]. A single visible pneumatopore is located on the middle portion of the caudal face of the quadrate of *Parahesperornis*. In *Hesperornis* this pneumatopore is displaced toward the distal end. In *Potamornis* no foramen is visible in this area.

**Palatines.** The palatines of *Parahesperornis* and *Hesperornis* have been somewhat contradictorily described by previous authors. What Marsh [7] initially identified as a vomer of *Hesperornis* (YPM 1206), Gingerich [58], working with both KUVP 2287 and YPM 1206, later described as a palatine, and what Marsh [7] referred to as a palatine Gingrich [58] described as a vomer. Gingerich [58,69] described the palatine as articulating with the pterygoid and tapering anteriorly to where it is fused with the vomer. There is no evidence of this relationship preserved today with *Parahesperornis* KUVP 2287. Working with KUVP 2287 before it was disarticulated, Gingerich [69] described both palatines as “virtually complete” and identified the left palatine as preserved alongside the frontals and a portion of the right palatine alongside the premaxilla (see Figure 4).

In his palatal reconstruction, Gingerich [69] placed the palatines as articulating with the pterygoids (Figure 4). While work by Witmer and Martin [70] supported Gingerich’s interpretation, later work by Elzanowski [71] rejected Gingerich’s [69] reassignment of the bones of YPM 1206 as the palatine and vomer, reverting to Marsh’s [7] original identifications. While Elzanowski did not specifically identify these elements in KUVP 2287, the bones identified as the palatines by Gingerich [58,69] in his reconstruction would be equivalent to the hemipterygoids of Elzanowski [56]. The present study supports Elzanowski’s interpretation of the hemipterygoids.

Today, a portion of the left palatine is preserved pressed against the ventral side of the frontals of KUVP 2287 (PT in Figure 5). The identification of the palatine of *Parahesperornis* KUVP 2287 as supported by this study is based on the distinctive hook present at the cranial end of this bone, which is difficult to interpret as belonging to any other element. The remainder of this palatine is difficult to identify. Historic photos demonstrate that the small rod preserved alongside this hook is not associated with the palatine and is instead most likely the hemipterygoid (see discussion above). It seems likely that some portion of the palatines is preserved in the jumble of thin bones crushed into the ventral surface of the premaxillae (Figure 7), however precisely identifying these bones is not possible.

**Figure 17:**
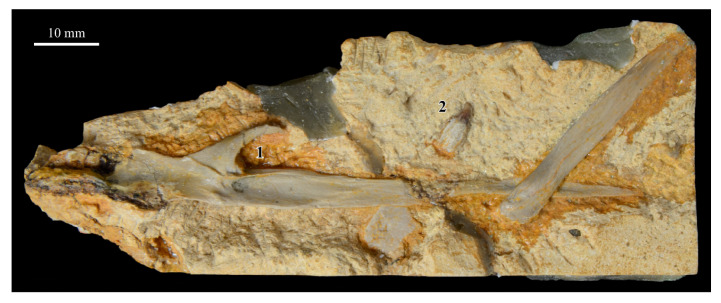
Palatine of *Hesperornis* KUVP 71012 showing caudally directed hook (1) and isolated tooth (2).

The preserved portion of the left palatine of *Parahesperornis* consists of a small, caudally directed hook, somewhat crushed into the shaft, which is elongate and very thin mediolaterally. The hook is much more compact than that of *Hesperornis*, which is elongate and reaches back further over the shaft (Figure 17). The midsection of the shaft of the left palatine of *Parahesperornis*, immediately rostral to the hook, appears to have a weakly-developed groove, which is more clearly seen in *Hesperornis*.

**Figure 18:**
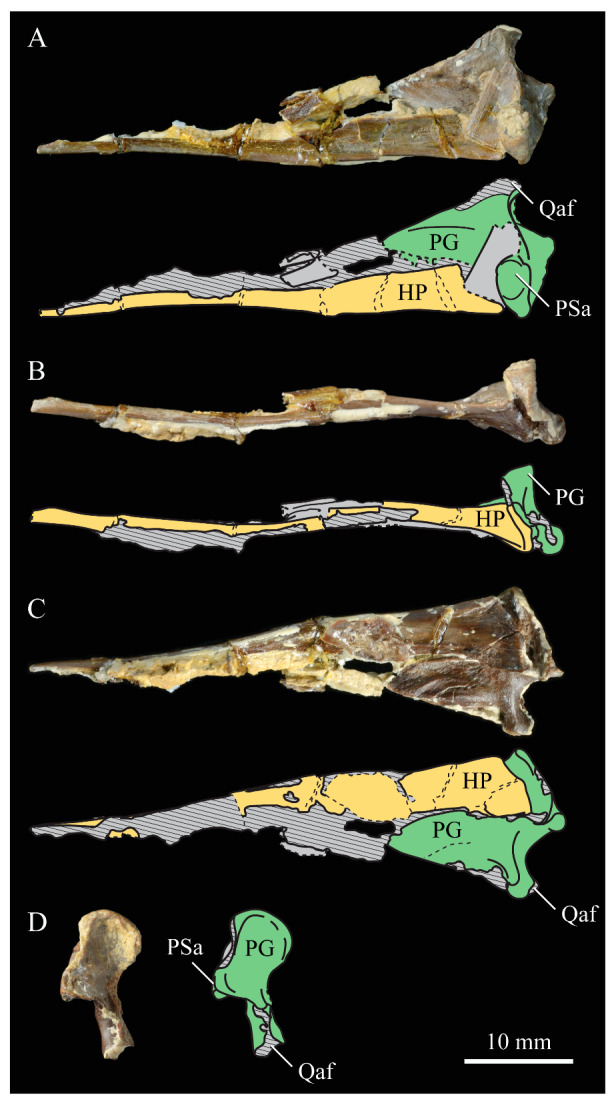
*Parahesperornis alexi* KUVP 2287, photographs (upper) and line drawings (lower) of the right pterygoid and hemipterygoid in medial (**A**), rostral (**B**), lateral (**C**), and ventral (**D**) views. Color coding: green—pterygoid; yellow—hemipterygoid; grey—unknown; cross-hatching—matrix. Abbreviations: HP—hemipterygoid, PG—pterygoid, PSa—parasphenoid articulation, Qaf—quadrate articular facet.

**Figure 19:**
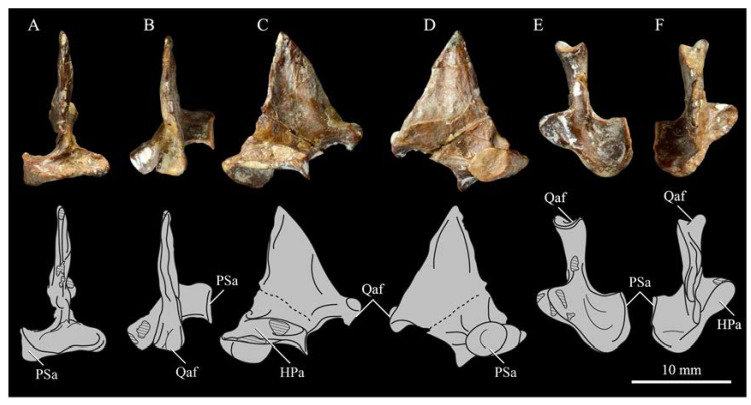
*Parahesperornis alexi* KUVP 2287, photographs (upper) and line drawings (lower) of the left pterygoid in rostral (**A**), caudal (**B**), lateral (**C**), medial (**D**), ventral (**E**), and dorsal (**F**) views. Abbreviations: HPa—hemipterygoid articulation, PSa—parasphenoid articulation, Qaf—quadrate articular facet.

**Figure 20:**
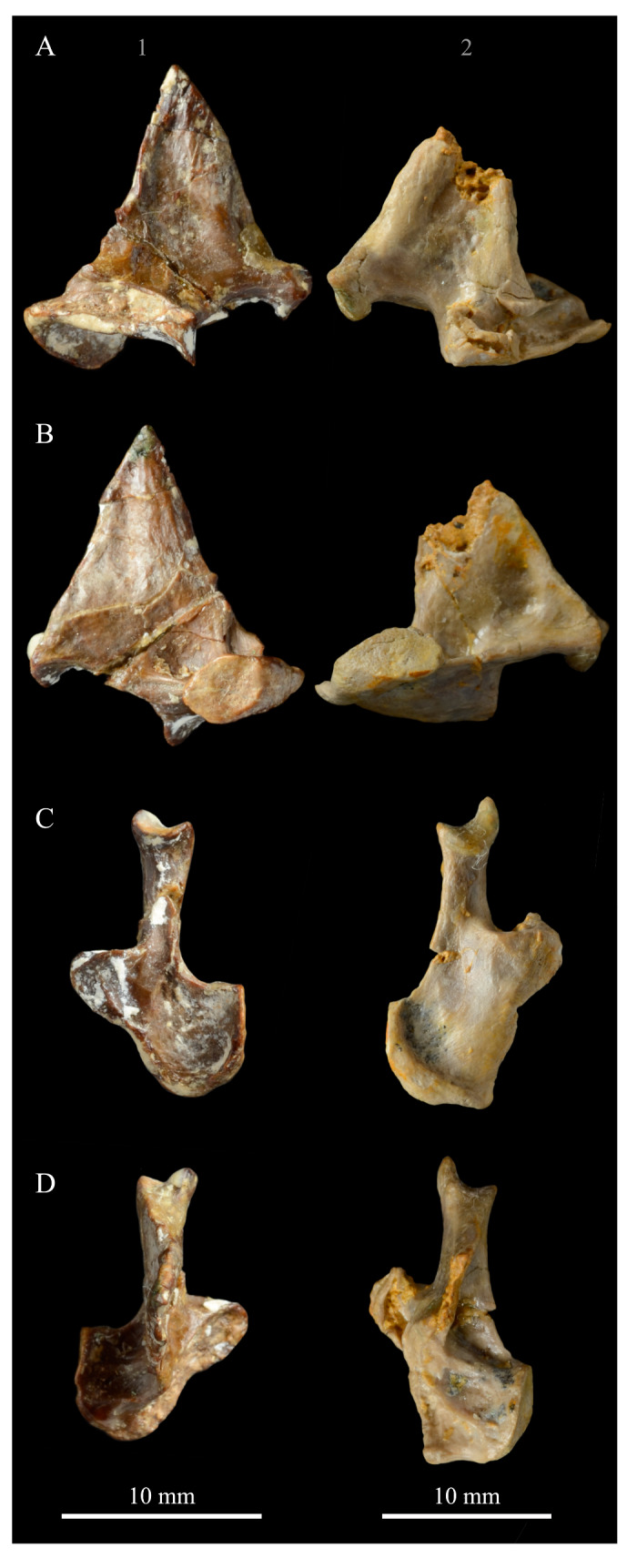
Comparison of the left pterygoid of *Parahesperornis* KUVP 2287 (1) and right pterygoid of *Hesperornis* KUVP 71012 (2) in lateral (**A**), medial (**B**), ventral (**C**), and dorsal (**D**) views. Specimens are scaled to be of similar size and aligned at articular facets.

**Pterygoids**. *Parahesperornis* KUVP 2287 preserves both pterygoids; the right is articulated with the right hemipterygoid (Figure 18), while the left is disarticulated (Figure 19). For comparison the pterygoid of *Hesperornis* (KUVP 71012, YPM 1206) was examined (Figure 20). The body of the pterygoid of *Parahesperornis* is triangular and very thin, with nearly flat sides. In *Hesperornis*, the sides are not as flat, with a depression near the articulation for the quadrate on the medial face and a broad groove developed dorsoventrally across the lateral face. The ventral margin of the lateral face of the pterygoid in *Parahesperornis* forms a broad, flattened c-shape, while in *Hesperornis* it is a deep, nearly enclosed u-shape.

The ventral surface of the pterygoid of *Parahesperornis* is semicircular and shallowly depressed with a thin crest along the lateral margin, while in *Hesperornis* it is almost rectangular with a thickened lateral margin bordered by a deep recess. The quadrate articulation of the pterygoid of *Parahesperornis* is a tiny facet at the caudal end of the triangular body that is more shallowly excavated than in *Hesperornis.* In *Hesperornis* a small pointed process is present on the medioventral margin of the pedicel for the parasphenoid articulation (best seen in dorsal view), however this process is absent in *Parahesperornis*, where the margin is smoothly rounded. In caudal view, the palatine articulation is more concave in *Parahesperornis* and flattened in *Hesperornis* with a well-developed lip around the caudodorsal margin.

**Figure 21:**
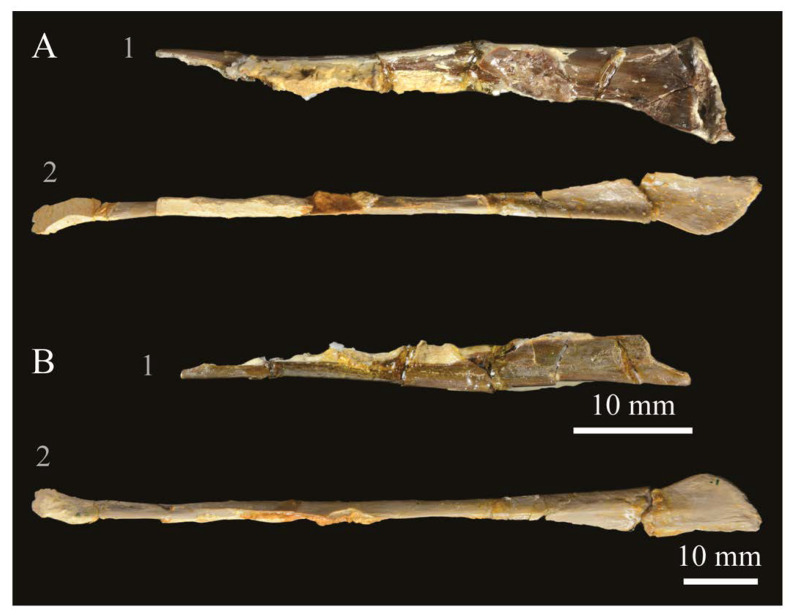
Comparison of the hemipterygoids of *Parahesperornis* KUVP 2287 (1) and *Hesperornis* KUVP 71012 (2) in lateral (**A**) and medial (**B**) views. Specimens are scaled to be of similar size, with the overlapping pterygoid digitally removed from 1.

**Hemipterygoid***. Parahesperornis* KUVP 2287 preserves both hemipterygoids; the left is preserved alongside the braincase and frontals and overlapping the left palatine (Figure 5), while the right is preserved separately and articulated with the right pterygoid (Figure 18). For comparison, the hemipterygoid of *Hesperornis* (KUVP 71012) was examined (Figure 21). While the left hemipterygoid identified here was described by Gingerich [69] as the left palatine, the preserved caudal end agrees much more with that of the right hemipterygoid, as both have a rounded ventral margin and an angled caudal margin.

The hemipterygoid is a very long, thin bone that widens caudally at the articulation with the pterygoid. This caudal expansion is more dramatic in *Parahesperornis* than in *Hesperornis*. The hemipterygoid appears not to have been fused or strongly sutured to the pterygoids, as indicated by the disarticulation of the elements. At the caudal end, the lateral and medial surfaces are relatively flat in *Parahesperornis*, while in *Hesperornis* the lateral surface is rounded and the medial is broadly indented.

**Figure 22:**
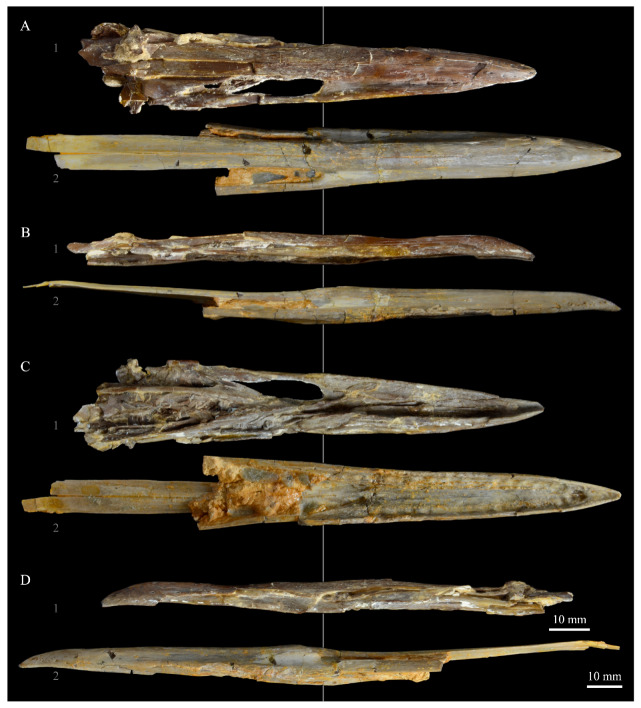
Comparison of the premaxillae of *Parahesperornis* KUVP 2287 (1) and *Hesperornis* KUVP 71012 (2) in dorsal (**A**), right lateral (**B**), ventral (**C**), and left lateral (**D**) views. Specimens are scaled to be of similar width at the opening of the nares and aligned at the rostral end of the nares.

**Premaxilla.** The premaxillae of *Parahesperornis* KUVP 2287 were preserved in articulation with the bulk of the skull (Figure 4) and are slightly crushed dorsoventrally and to the left side (Figure 7). The caudal ends appear to narrow before the broken ends, indicating that perhaps not much length has been lost due to breakage. For comparison the premaxilla of *Hesperornis* (KUVP 71012, YPM 1206) was examined (Figure 22). Despite being of a similar width at the rostral end of the nares as *Hesperornis* (and accounting for the slight crushing to the element), the length from the tip of the premaxilla to the opening of the nares is much shorter in *Parahesperornis* (width at the nares is approximately 35% the pre-nares length) than in *Hesperornis* (width at the nares is approximately 20% the pre-nares length), implying a more elongate skull in the latter.

The premaxillae are edentulous with a straight tomial margin, ending in a slight terminal hook, similar to that in *Hesperornis* and other Mesozoic ornithuromorphs (*Dingavis* [76], *Changzuiornis* [79]) but much shallower than in *Ichthyornis*. The premaxillae of hesperornithids were quite long and narrow, making up close to half the length of the rostrum, as compared to the shorter premaxillae in some other ornithuromorphs (i.e., *Ichthyornis, Yanornis*). This is likely a foraging adaptation for the marine lifestyle of hesperornithiforms. Across Mesozoic birds, lengthening of the rostrum is commonly accomplished by lengthening of the maxilla, contrasted by the elongation of the premaxilla common in Neornithes [76]. While the maxilla does not appear to be preserved in *Parahesperornis* KUVP 2287, *Hesperornis* KUVP 71012 preserves a fairly complete maxilla that indicates *Hesperornis* achieved rostral elongation in the same way that modern birds do, through elongation of the premaxillae, and unlike the maxillary elongation documented in other Mesozoic birds [76].

In dorsal view, the suture between the left and right premaxillae of *Parahesperornis* is obvious caudally along the entire length of the frontal processes; it becomes fainter rostrally, disappearing about an inch before the rostral-most extent of the premaxillae. In *Hesperornis*, this suture is not visible for as much of its length. Both birds have a series of pinhole neurovascular foramina dotting the rostral-most end of the premaxilla, possibly indicating the presence of a keratinized ramphotheca. The maxillary process of the premaxilla is articulated to the maxillary process of the nasal in *Parahesperornis* KUVP 2287 (Figure 7). In ventral view the premaxillae of *Parahesperornis* lack impressions for the fitting of the dentary teeth, which are visible in *Hesperornis* and some other toothed birds (e.g., *Ichthyornis*). Both *Parahesperornis* and *Hesperornis* have a small pair of teardrop-shaped pits in the very tip of the ventral premaxillae (f in Figure 7).

**Figure 23:**
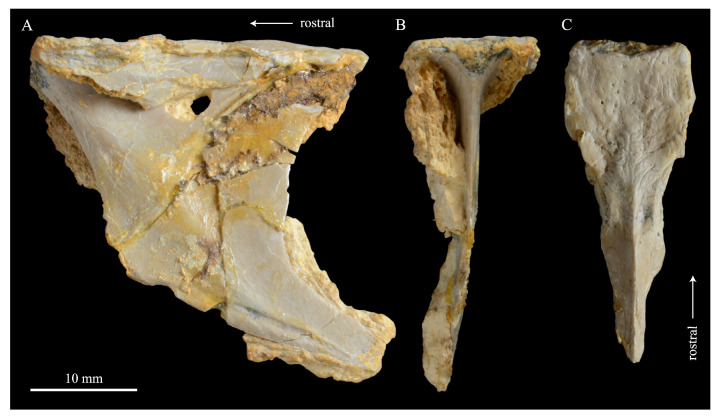
Mesethemoid of *Hesperornis* (KUVP 71012) in left lateral (**A**), rostral (**B**), and dorsal (**C**) views.

**Mesethmoid**. A bone which may represent the mesethmoid in *Parahesperornis* KUVP 2287 is preserved crushed against the ventral surface of the frontals, adjacent to the palatine (ME in Figure 5). If this identification is correct, then the bone is lying on its side and has been crushed into the frontals. This bone is broad along the dorsal margin and narrow at the ventral edge. This is similar to the more complete mesethmoid of *Hesperornis* as preserved with KUVP 71012 (Figure 23).

The mesethemoid has been reconstructed as fitting into the gap between the separate rostral-most ends of the frontals [57], terminating at the anterior end of the frontals, as has recently been described for *Ichthyornis* [61]. Unfortunately, the degree of crushing in KUVP 2287 does not provide additional information about this arrangement, however the current placement of the mesethemoid and the degree of separation of the frontals does not contradict this interpretation.

**Figure 24:**
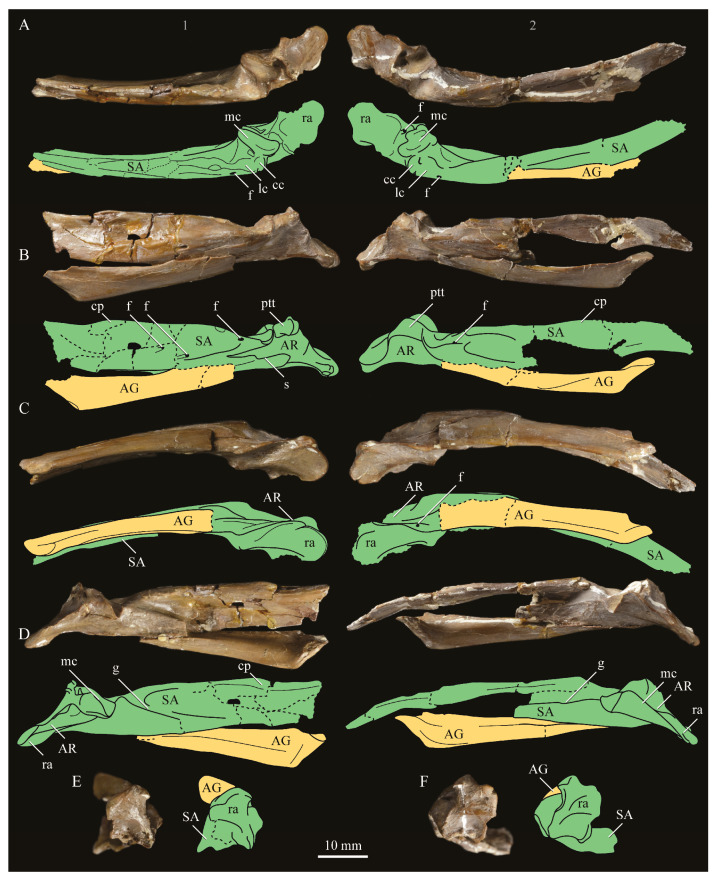
*Parahesperornis alexi* KUVP 2287, photographs (upper) and line drawings (lower) of the left (1) and right (2) articulated angulars, surangulars, and articulars of the mandible in dorsal (**A**), lateral (**B**), ventral (**C**), medial (**D**), and caudal (**E**,**F**) views. Abbreviations: AG—angular, AR—articular, cc—caudal cotyla, cp—coronoid process, f—foramen, g—groove, lc—lateral cotyla, mc—medial cotyla, ptt—pseudotemporale tubercle, ra - retroarticular process, s—suture, SA—surangular.

**Figure 25:**
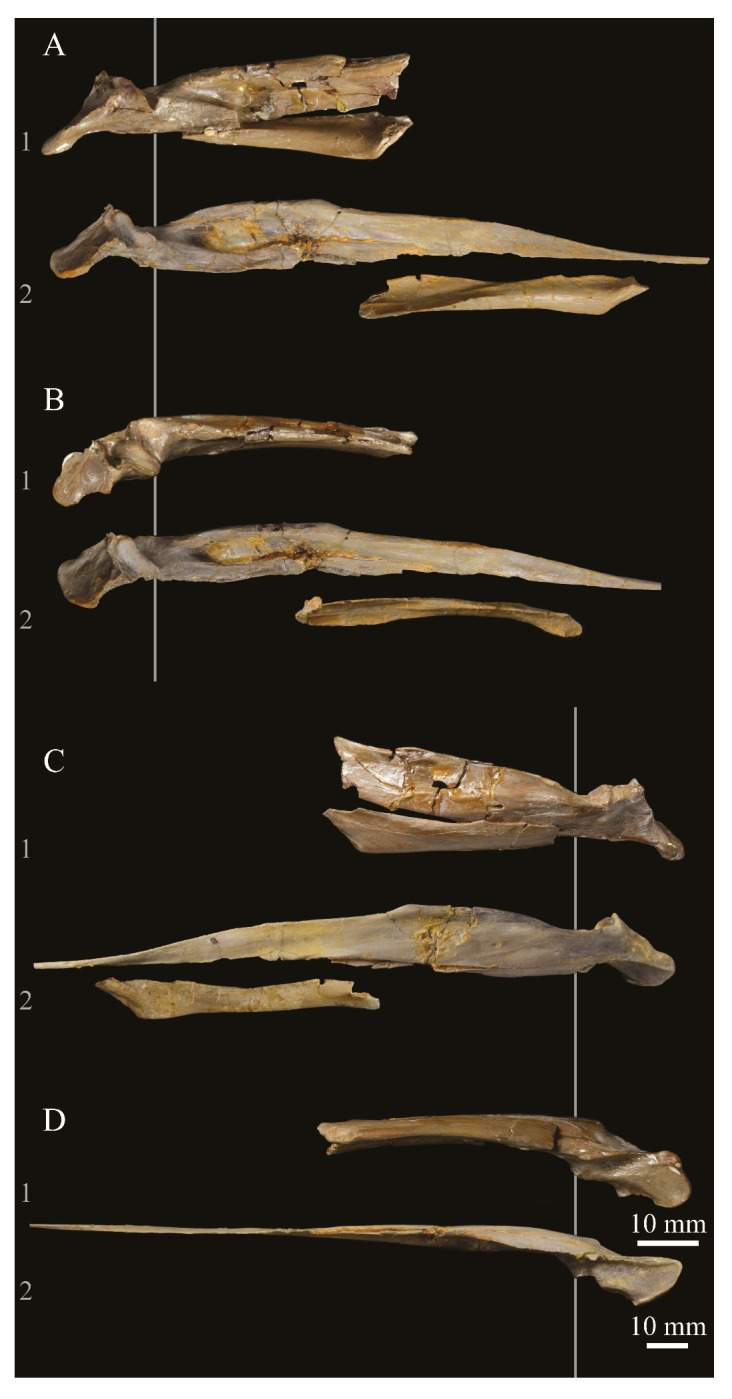
Comparison of the left angulars, surangulars, and articulars of *Parahesperornis* (KUVP 2287; (1) and *Hesperornis* (KUVP 71012; (2) in medial (**A**), dorsal (**B**), lateral (**C**), and ventral (**D**) views. Specimens are scaled to be of similar size and aligned at the medial cotyla of the articular.

**Angulars.** Both angulars of *Parahesperornis* KUVP 2287 are preserved and have been glued into rough articulation with the articulars and surangulars (Figure 24). For comparison the angular of *Hesperornis* (KUVP 71012, YPM 1206) was examined (Figure 25). While angulars have been assigned to *Pasquiaornis* [65], these bones are highly fragmentary and provide little comparative information.

The angular of *Parahesperornis* is straight along its ventral margin and bows slightly in dorsal or ventral view, as in *Hesperornis*. The articular surfaces are somewhat obscured caudally by the surangulars. In medial view, the articular surface forms a faint, broad ridge bounded laterally by the thin, flat lateral side of the bone. At the caudal-most end, the articular surface transitions to a flat, dorsally facing surface that is glued onto the surangular in KUVP 2287. In *Hesperornis* the surangulars are disarticulated, making the entire articular surface visible. The caudal-most end of this articular surface is broad and concave, narrowing rostrally and developing a lateral ridge as in *Parahesperornis*. However, at the rostral-most end the articular surface of *Hesperornis* forms a slightly expanded, concave surface unlike the flattened surface of *Parahesperornis*. The morphology of the dorsal surface implies that the angular *Parahesperornis* articulated with the surangular through a broader, sheeted surface along its length, while that of *Hesperornis* was somewhat more restricted to the concave surface.

It should be noted that due to the breakage of the ventral margin of the surangular, the angulars of KUVP 2287 have been glued on imperfectly. The present reconstruction of the left, and more complete, mandible has a slight gap between the surangular and rostral angular. This gap has also been figured in reconstructions of the lower jaw of *Hesperornis* that were based off this specimen [58,59]. However, the surangulars and angulars of *Hesperornis* KUVP 71012 are complete enough to allow a good articulation, and the angular fits smoothly and completely over the surangular, with no gap.

Surangulars. The surangulars of *Parahesperornis* KUVP 2287 are preserved in articulation with the articulars and glued to the angulars but crushed and poorly preserved (Figure 24). For comparison the surangulars of *Baptornis* (FMNH 395) and *Hesperornis* (KUVP 71012, YPM 1206) (Figure 25) were examined. The surangular of *Parahesperornis* is a very thin bone that articulates with the dorsal surface of the articular just cranial to the pseudotemporale tubercle, where a suture is visible (Figure 24). In dorsal view, both surangulars of *Parahesperornis* are convex, bowing out laterally to a much greater degree than in *Hesperornis*.

The medial face of the surangular of *Parahesperornis* has a broad, shallow depression. A mandibular foramen is not present. The mandibular foramen is also absent in *Hesperornis,* contra Marsh^7^. A slight groove is present running diagonally from the ventral margin to the dorsal margin, opening into this depressed area. In *Hesperornis* this groove is not present, and the depression on the medial face is much more deeply excavated than in *Parahesperornis*. The lateral face of the right surangular of *Parahesperornis* KUVP 2287 preserves three foramina, one located near the dorsal margin by the articular and two nearly centered on the lateral surface. While the foramen near the dorsal margin is not present in *Hesperornis*, the central ones are visible in a similar location. The surangular of *Baptornis* is minimally preserved, with FMNH 395 only preserving the caudal-most end, which is fully fused to the angular, with a groove marking this fusion in medial view, as in hesperornithids.

**Articulars.** Both articulars of *Parahesperornis* KUVP 2287 are preserved in articulation with the surangulars (Figure 24). For comparison the articulars of *Baptornis* (FMNH 395), *Hesperornis* (KUVP 71012; Figure 25), and *Pasquiaornis* (RSM P2989.21, RSM P2989.19) [65] were examined. The retroarticular process of *Parahesperornis* is somewhat oval and dorsally concave, with raised edges that almost form a lip, while that of *Hesperornis* appears to be proportionally broader and flatter. The retroarticular process of *Parahesperornis* is extremely elongate, slightly more so than in *Hesperornis*, however the elongation in both taxa is extreme compared to modern birds. The retroarticular process in *Baptornis* is more like that of modern birds, with the caudal end the same width as the remainder of the element and extending only slightly caudally beyond the cotylae. The articular is not fully fused to the surangular, with a suture visible in both *Parahesperornis* and *Hesperornis*. In *Baptornis* these elements appear fully fused, with no suture present, at least at the preserved caudal end.

Rostral to the retroarticular process is a small but very deep depression separating the retroarticular process from the medial articular cotyla of the quadrate articulation. This deep fossa is not present in *Hesperornis* or *Baptornis* but is in *Pasquiaornis*. The medial cotyla is developed as a deep groove oriented almost transversely and angled such that the lateral edge is ventral to the medial edge. In *Hesperornis* the medial cotyla is oriented even more transversely than in *Parahesperornis.* The degree of excavation of the medial cotyla is highest in *Parahesperornis,* reduced but still prominent in *Hesperornis* and *Pasquiaornis,* and faint in *Baptornis.* This cotyla is located primarily on the dorsal surface of the mandible in *Parahesperornis*, as is also the case in *Baptornis,* but slightly more offset to the medial side of the mandible in *Hesperornis* and located almost entirely on the medial side of the mandible in *Pasquiaornis*.

Medial and slightly rostral to the medial cotyla in *Parahesperornis,* the caudal cotyla is developed as a small oval facet that is bounded by prominent ridges on all but the rostral margin, which is indistinct. The caudal cotyla in *Hesperornis* is directly medial to the medial cotyla and not offset rostrally. It is only faintly developed and lacks the prominent margins in *Parahesperornis.* The caudal cotyla is not discernible in *Baptornis* FMNH 395. The lateral cotyla of *Parahesperornis* is very faint and not clearly divided from the caudal, constituting a very faint, flattened face rostral to the caudal condyle. This is also true for *Hesperornis.*

**Figure 26:**
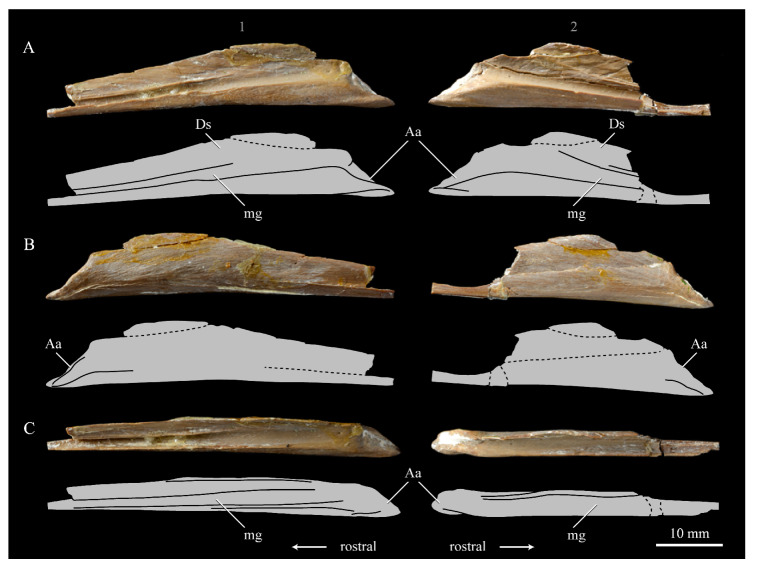
*Parahesperornis alexi* KUVP 2287, photographs (upper) and line drawings (lower) of the left (1) and right (2) splenials in lateral (**A**), medial (**B**), and dorsal (**C**) views. Abbreviations: Aa—articular surface for angular, Ds—sheet of bone overlaying dentary, mg—Meckel’s groove.

**Figure 27:**
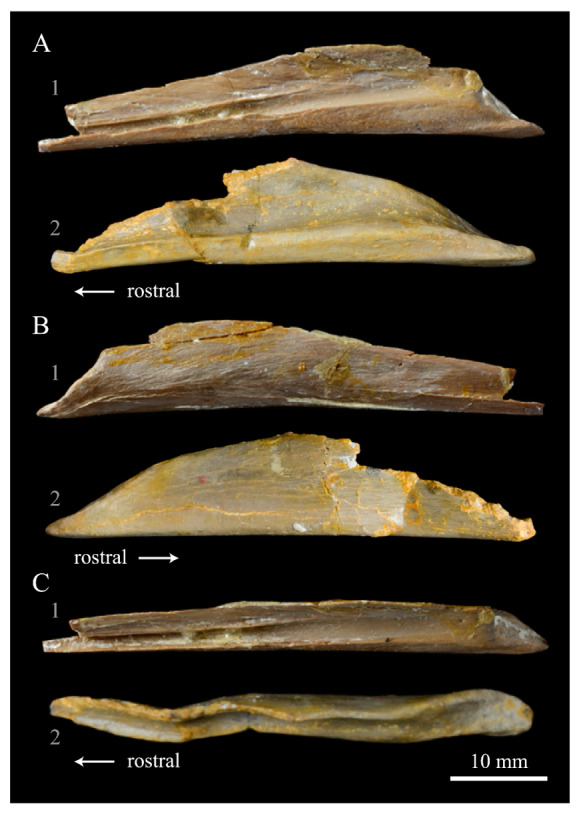
Comparison of the splenials of *Parahesperornis* KUVP 2287 (1) and *Hesperornis* KUVP 71012 (2) in medial (**A**), lateral (**B**), and dorsal (**C**) views.

**Splenials.** Portions of both splenials are preserved as isolated elements with *Parahesperornis* KUVP 2287 (Figure 26). For comparison the splenials of *Hesperornis* (KUVP 71012) and *Pasquiaornis* (RSM P2985.9) [65] were examined (Figure 27). The splenial of *Parahesperornis* has a slightly rounded lateral surface and a medial surface divided lengthwise by Meckel’s groove, which forms a slight shelf for the dentary. Above this shelf the splenial is very thin and appears to form a sheet over the ventral bladed portion of the dentary. This sheeted overlay of the splenial on the dentary is unusual in birds, but is also seen in *Ichthyornis* [61].

The caudal end of the splenial angles ventrally to form an articular surface for the angular. The splenial of *Parahesperornis* is very similar to that of *Hesperornis*, including in size, despite the overall larger size of the mandible of *Hesperornis*. In *Parahesperornis* the caudal end of the articular surface for the dentary angles more sharply ventrally at the caudal end than in *Hesperornis*, while in *Pasquiaornis*^65^ the transition is smooth and broad, more a curve than an angle.

**Figure 28:**
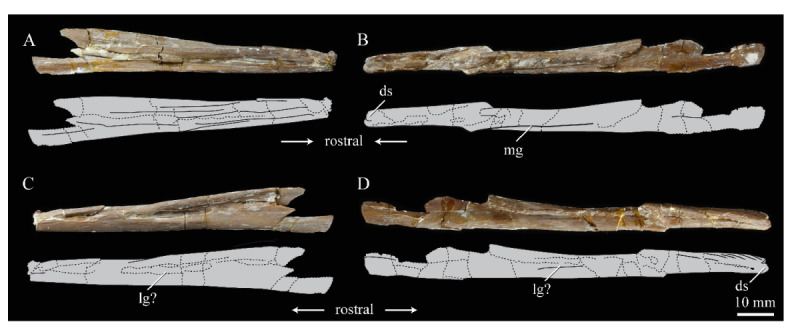
*Parahesperornis alexi* KUVP 2287, photographs (upper) and line drawings (lower) of left (**A**,**C**) and right (**B**,**D**) dentaries in medial (**A**,**B**) and lateral (**C**,**D**) views. Abbreviations: ds—dentary symphysis, mg—medial groove, lg?—possible lateral groove.

**Figure 29:**
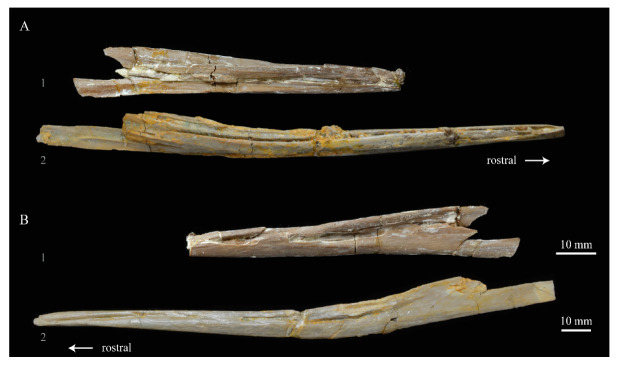
Comparison of the dentaries of *Parahesperornis* KUVP 2287 (1) and *Hesperornis* KUVP 71012 (2) in medial (dorsomedial in *Hesperornis* due to crushing) (**A**) and lateral (**B**) views. Specimens are scaled to be similarly sized.

**Dentaries.** The dentaries of *Parahesperornis* KUVP 2287 are poorly preserved, being incomplete and crushed (Figure 28). The right dentary preserves more of the length than the left, including the symphysis. For comparison, the dentaries of *Hesperornis* (KUVP 71012, YPM 1206) (Figure 29) and *Pasquiaornis* (RSM P 2831.60, RSM P 2985.10, RSM P 2988.11) [65] were examined, as well as a partial dentary assigned to *Asiahesperornis* (IZASK 4/KM 97, but see discussion below) [80]. All hesperornithiform dentaries show partial individual sockets for the teeth, opening into a dental groove running along the dorsal surface of the dentary [81] (a dental implantation similar to the ‘aulacodonty’ of non-avian reptiles [82]. While these partial sockets have been described as “faint” and insufficient to reduce the width of the dental groove [7], specimens that have broken open show the sockets to be clear divisions within the dentary, but not true sockets as in *Ichthyornis* (e.g., *Hesperornis* YPM 1206 and *Pasquiaornis* RSM P 2985.10 and RSMP P 2988.11). The dentary fragment assigned to *Asiahesperornis* (IZASK 4/KM 97) [80] shows clear alveoli for the teeth. As this is not the case among other hesperornithiforms, and the *Asiahesperornis* material consists entirely of unassociated elements, it is unlikely this specimen belongs to a hesperornithiform bird.

In *Parahesperornis*, the dental grooves appear similar to those of *Hesperornis* and expand at the caudal-most end, however the degree of crushing makes identification of more specific features difficult. The dentaries of *Parahesperornis* appear to preserve a longitudinal groove along the lateral face, as in *Hesperornis* and *Pasquiaornis*, however the degree of crushing makes this identification tentative. A pair of medial grooves have been reported in *Pasquiaornis*, and the right dentary of *Parahesperornis* appears to have a short groove present near the ventral margin. *Hesperornis* appears to have a single groove running down both the lateral and medial faces of the dentary.

The symphysis preserved on the right dentary consists of two small bulbous projections stacked vertically. This configuration is also present in *Pasquiaornis* (RSM P 2831.6). Some specimens of *Hesperornis* (i.e., KUVP 71012) do not show this, having instead a smooth symphysis that tapers to a point (Figure 29), while others do appear to have the rounded projections (YPM 1206).

**Figure 30:**
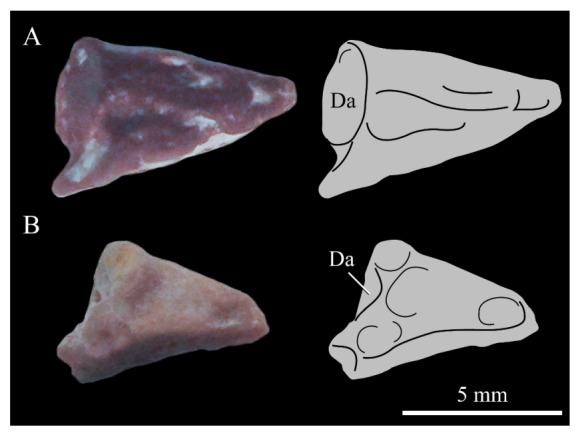
Predentaries of *Parahesperornis* KUVP 2287 (**A**) and *Hesperornis* KUVP 71012 (**B**) in right lateral view. Abbreviation: Da—dentary articular surface.

**Predentaries**. Martin first reported the existence of a predentary bone in ornithuromorphs when he described those of hesperornithids [83]. Within the Hesperornithiformes to date, this bone has only been reported for a single specimen of *Hesperornis* (KUVP 71012) and *Parahesperornis* (KUVP 2287) [73] (Figure 30). The predentary is known in other Mesozoic ornithuromorphs, including *Ichthyornis*, *Yanornis, Yixianornis, Hongshanornis,* and *Jianchangornis* [84,85].

The predentary of *Parahesperornis* possesses a pair of small facets on the caudal face, the right of which is deformed and flattened. In *Hesperornis* KUVP 71012, a single facet appears to be present; however, it is possible that weathering of the element could have obscured the division of the facets. The predentary of *Parahesperornis* is more elongate than that of *Hesperornis*. While the predentary of *Hesperornis* is certainly more weathered than that of *Parahesperornis*, this is not enough to account fully for the size discrepancy. Recent work has hypothesized a unique mandibular kinesis in ornithuromorphs that possess a predentary, with a synovial joint involving ventral flexion of the dentaries and translation and compression of the predentary on the dentaries [85].

**Teeth.** *Parahesperornis* KUVP 2287 preserves several teeth, however none are in place in the dentaries, the only identifiable tooth-bearing bone. At least four teeth are visible in the underside of the premaxilla. These teeth could have come from the maxilla or dentaries. Two are easily visible and include the root and crown (Figure 7 and Figure 8). Martin [40] described the teeth of *Parahesperornis* as less pointed and recurved than those of *Hesperornis.* That observation is likely not accurate, as along the jaw the teeth of *Hesperornis* change shape slightly, becoming more recurved rostrally and less recurved caudally [81]. This appears to be true for *Parahesperornis* as well, as the two teeth preserved near each other in the ventral premaxillae are of different curvatures, however their placement within the jaw is unknown so this supposition cannot be confirmed (Figure 8). A comparison of both teeth preserved in the ventral surface of the premaxilla with the isolated tooth of *Hesperornis* KUVP 71012 preserved near the palatine (Figure 17) shows them to be largely similar, but smaller in size. Similar teeth have also been reported for *Pasquiaornis* [65], however none have been found associated with identifiable skeletal material.

**Unidentified Palatal Elements**. The ventral surfaces of the caudal premaxillae are filled with numerous small, thin bones jumbled together (Figure 7 and Figure 8). While the interpretations of the nasals and premaxillae presented here seem likely, other identifications are less so. At first glance it appears that there are two bones that form a V-shape and would perhaps be the left and right of the same element. However, close examination reveals that the left “bone” is actually three separate bones stacked together. Furthermore, none of these bones have the smoothly rounded ventral margin displayed by the right bone; rather, all three have thin, sharp edges. Of these three bones on the left, the lateral-most bone is the longest, extending as a thin blade past the nares.

Unfortunately, none of these bones can be positively identified at this time, however it is possible they may be portions of the jugals, quadratojugals, maxillae, or vomers, none of which have been identified thus far. It should be noted that there appears to be a tooth preserved in the center of this area in historical photographs (Figure 4), however the tooth is no longer present today.

#### 3.2.2. Vertebrae

*Parahesperornis* KUVP 2287 preserves 22 presacral vertebrae, including the axis, and five caudal vertebrae, while KUVP 24090 preserves at least 17 presacral vertebrae, four caudal vertebrae, and the pygostyle. The posterior cervical and thoracic vertebrae of KUVP 24090 are predominately articulated and encased in chalk while those of KUVP 2287 are fully prepared, with some isolated and some articulated sections. The vertebrae of *Parahesperornis* discussed here have been identified through a comparison to Marsh’s [7] figures of *Hesperornis*, which were based primarily on YPM 1207, YPM 1476, YPM 1477, and YPM PU 18589, as well as articulations that can be recreated between disarticulated elements.

The process of identification is complicated by the general similarity of neighboring vertebrae in some sections of the vertebral column. For example, vertebrae 18 to 21 are very similar in hesperornithiforms, making their precise identification in *Parahesperornis* difficult. However, the morphology of the more cranial vertebrae of hesperornithiforms is distinctive, making certain vertebrae easily identifiable. The following specimens were used for comparison of the presacral vertebrae: *Baptornis* (AMNH 5101, KUVP 2290, KUVP 16112); *Canadaga* (NMC 41050); *Chupkaornis* (MCM.A773) [12]; *Enaliornis* (SMC B55277, SMC B55279, SMC B55280); *Fumicollis* (UNSM 20030); *Hesperornis* (KUVP 2280, KUVP 2289, KUVP 71012, UNSM 4-19-5-36, YPM 1200, YPM 1207, YPM 1476, YPM PU 18589); and *Pasquiaornis* (RSM P2831, RSM P2988) [65].

The presacral vertebrae of hesperornithids can be divided into five general morphotypes to facilitate description [20]. While each vertebra is unique in terms of small details of morphology, there are several trends that can be used to identify the approximate location in the vertebral column. The presacral vertebrae of *Parahesperornis* are described below, in terms of the main morphotypes occurring along the sequence. Marsh [7] designated the first seventeen vertebrae of *Hesperornis* as the cervicals, with the last three (15 to 17) having free ribs. The atlas, or 1st cervical vertebra, is unknown for both *Hesperornis* and *Parahesperornis*.

**Figure 31:**
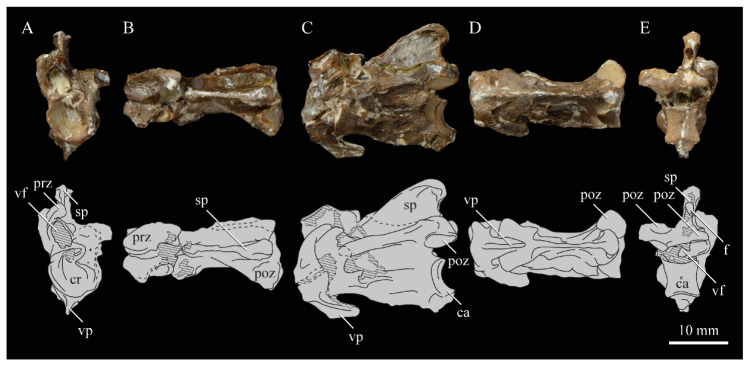
*Parahesperornis alexi* KUVP 2287, photographs (upper) and line drawings (lower) of axis in cranial (**A**), dorsal (**B**), lateral (**C**), ventral (**D**), and caudal (**E**) views. Abbreviations are as follows: ca—caudal articular surface, cr—cranial articular surface, poz—postzygapophysis, prz—prezygapophysis, sp—spinal process, vf—vertebral foramen, vp—ventral process.

**Axis.** The axis, or second cervical vertebra, is preserved in *Parahesperornis* KUVP 2287. It is somewhat crushed laterally and wedge-shaped in side view (Figure 31). The cranial articular surface forms a shallow, ovate dish, below which is a small, partially preserved ventral process. This process is not seen on the axis of *Hesperornis* (YPM 1207, KUVP 71012). The dens is crushed back against the body of the axis, with no discernable features. In *Hesperornis* (YPM 1207) the dens extends quite far cranially from the front of the axis, ending in a point in lateral view. Much of the sides of the centrum of KUVP 2287 are obscured by crushing. Near the craniocaudal midpoint of the centrum, the complete spinal process expands upward from the dorsal surface, sloping caudally. The caudal-most portion of the spinal process is rounded and blunt, extending to a height roughly one-third of the total dorsoventral height of the vertebra. This is similar in *Hesperornis*. However, the spinal process extends caudally past the end of the vertebral body.

The left postzygapophysis is completely preserved and consists of a domed dorsal surface over a shallow, ventrally directed facet that extends laterally. In *Hesperornis* the postzygapophyses are capped by high crests roughly 75% the height of the spinal process. In the axis of *Parahesperornis,* the caudal articular surface is taller dorsoventrally than it is wide, with a slight depression along the dorsal margin and a deeper depression on the ventral margin. The center of the caudal articular surface is marked by a small, circular depression that has been suggested to represent the trace of the notochord [19]. A small projection extends ventrally from the caudal body of the axis in *Parahesperornis*, while in *Hesperornis,* this expansion forms a prominent crest nearly as large as the spinal process.

**Figure 32:**
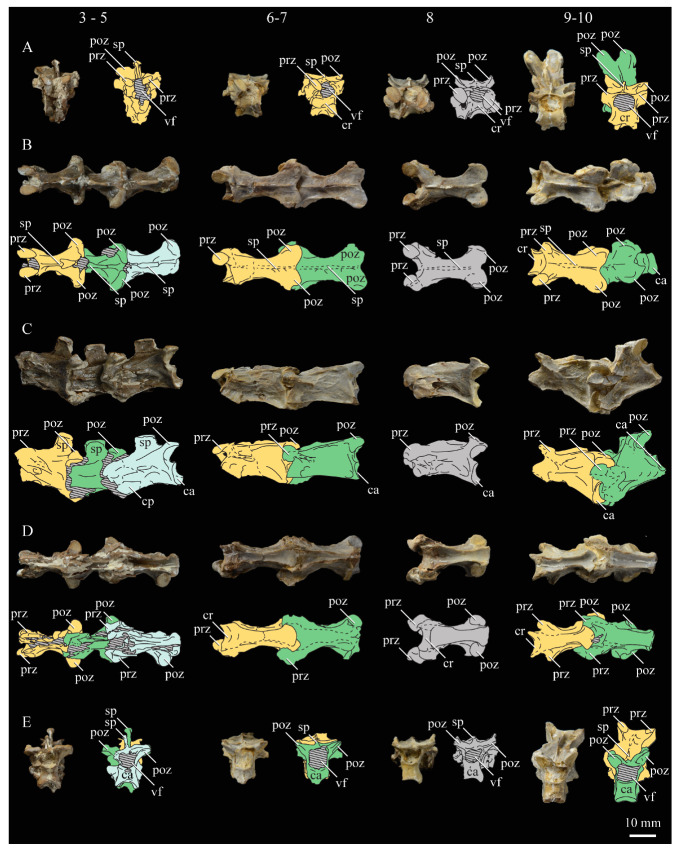
*Parahesperornis* KUVP 2287, photographs and line drawings of cervical vertebrae: articulated third (yellow), fourth (green), and fifth (grey) (3–5); articulated sixth (yellow) and seventh (green) (6–7); isolated eighth (8); and articulated ninth (yellow) and tenth (green) (9–10) vertebrae in cranial (**A**), dorsal (**B**), lateral (**C**), ventral (**D**), and caudal (**E**) views. Hatch marks indicate areas obscured by matrix. Abbreviations are as follows: ca—caudal articular surface, cp—costal process, cr—cranial articular surface, poz—postzygapophysis, prz—prezygapophysis, sp—spinal process, vf—vertebral foramen.

**Figure 33:**
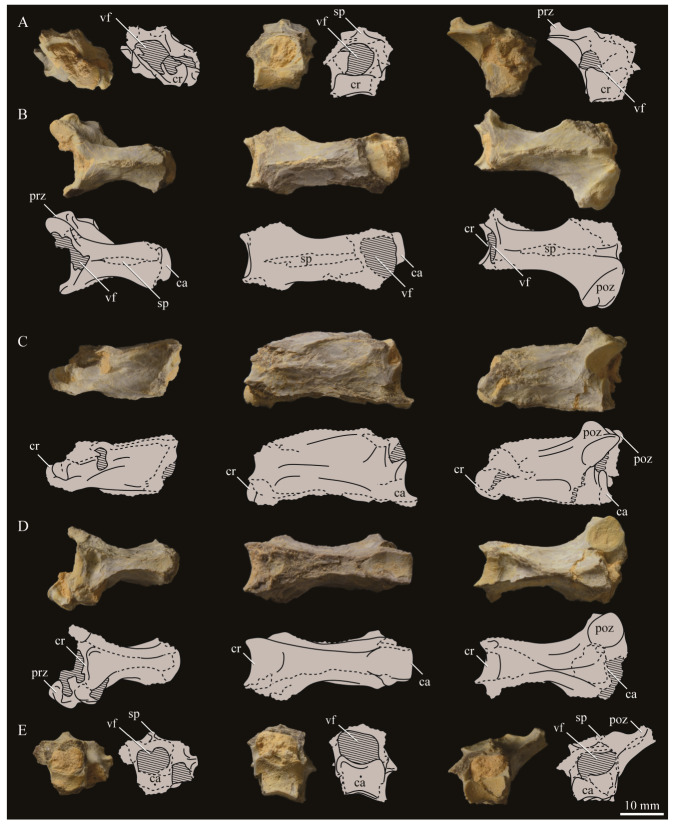
*Parahesperornis alexi* KUVP 24090, photographs and line drawings of cervical vertebrae 4 (left), 5 (center), and sixth or seventh (right) vertebrae in cranial (**A**), dorsal (**B**), lateral (**C**), ventral (**D**), and caudal (**E**) views. Hatch marks indicate areas obscured by matrix. Abbreviations are as follows: ca—caudal articular surface, cr—cranial articular surface, poz—postzygapophysis, prz—prezygapophysis, sp—spinal process, vf—vertebral foramen.

**Cervical Vertebrae 3 to5**. The third throughfifth cervicals of *Parahesperornis* KUVP 2287 are preserved in articulation (Figure 32). Their placement was identified by the ability of the third vertebra to articulate with the axis, as well as the similar shapes of the centra in dorsal view to those of *Hesperornis*. Most of the fourth and the dorsocaudal portion of the fifth cervical vertebrae are preserved as isolated elements in *Parahesperornis* KUVP 24090 (Figure 33). The placement of these vertebrae was identified primarily by the shape of the postzygapophyses, which are broad and fan shaped, with only a slight indentation between the zygapophyses.

In hesperornithids, cervical vertebrae three through five are characterized by a triangular shape in dorsal view with a narrow cranial end and broad caudal end. These vertebrae in *Parahesperornis* are very narrow mediolaterally with a grooved ventral surface that expands into a fan at the caudal end. The ventral surface is very narrow in the third cervical and broadens along the series. While the anterior cervical vertebrae of *Baptornis* are not well known, AMNH 5101 preserves two fragmentary vertebrae that are likely the third and fourth or fourth and fifth cervicals. The ventral surface of these vertebrae is much narrower than in hesperornithids, compressed into a thin crest at the caudal end of the third or fourth vertebra. A cervical proposed as being in the interval of cervical three to six was reported with the holotype of *Brodavis varneri* (SDSM 68430) [31], however the specimen is encrusted with gypsum and preserves little detail. Anterior cervical vertebrae have not been reported for any other hesperornithiform taxa.

In lateral view the centrum of the third cervical has a profile that is dorsoventrally tallest at the caudal end and narrows dramatically toward the cranial end, similar to, but less dramatic than, that of the third cervical of *Hesperornis*. In hesperornithids, this profile becomes progressively less exaggerated in the fourth and fifth cervicals, such that the fifth appears more rectangular in lateral profile. The lateral sides of the centra are nearly flat, with a slight craniocaudal groove along the ventral margin in *Parahesperornis* and *Hesperornis.* The costal processes of these anterior cervicals in *Parahesperornis* are only known from a short fragment preserved with the fifth cervical of KUVP 2287. Dorsal to where these processes would be in *Parahesperornis* is a small, oval depression.

The cranial end of the third vertebra of *Parahesperornis* KUVP 2287 is crushed and that of the others are obscured by articulation. The prezygapophyses are small and closely set, with oval articular surfaces facing dorsomedially, as in *Hesperornis*. Progressively along the series the prezygapophyses become more widely spaced. The spinal processes are well-preserved and offset caudally on the vertebral body. The spinal processes of these vertebrae are not preserved in any *Hesperornis* specimen available for study; however, from the broken surfaces they appear similarly located. In lateral view, the spinal processes of *Parahesperornis* become progressively craniocaudally longer and dorsoventrally shorter in the series, such that the spinal process of the fifth vertebra is almost twice the length of the third but only 75% the height.

The postzygapophyses are robust and expand laterally, making the dorsal outline of the vertebrae triangular. Along the series the articular facets become larger. The dorsal surfaces form peaks that become progressively lower and more rounded along the series. Breakage of the corresponding vertebra of *Hesperornis* makes these features difficult to compare; however, in the fifth cervical of YPM 1207 the dorsal surface of the left postzygapophysis is very highly peaked and does not appear rounded like that of *Parahesperornis*. Unlike the articular surfaces of the postzygapophyses of the preceding vertebrae, which are directed ventrally, those of the fifth cervical of *Parahesperornis* are directed slightly ventrolaterally, similar to the orientation of the fourth and fifth cervicals in *Hesperornis*.

**Cervical Vertebrae 6 to 9**. Within this series, *Parahesperornis* KUVP 2287 preserves the articulated sixth and seventh vertebrae, the isolated eighth, and the ninth articulated to the tenth (Figure 32), while KUVP 24090 preserves the isolated sixth or seventh vertebrae (Figure 33). The sixth to ninth cervical vertebrae of hesperornithids can be characterized by the division of the postzygapophyses into two distinct articular surfaces in dorsal view, which become progressively separated along the series. In dorsal view these vertebrae resemble an elongate X-shape instead of the triangular outline of the more anterior vertebrae. Other trends that began in the previous vertebrae are continued, such as the broadening of the ventral surface of the centra.

In cranial view, the articular surface of the sixth cervical narrows dramatically dorsoventrally in the center and wraps around and under the ventral margin of the centrum, expanding into the costal process on the sides (were they preserved). Further along the series, the center expands and is not as narrow. This is also seen in *Hesperornis.* The prezygapophyses are more circular than in the preceding vertebrae, with the articular facets steeply angled toward the midline of the centrum.

In lateral view, vertebrae six and seven are nearly rectangular in outline, with the caudal end only slightly taller than the cranial, and slightly more elongate than the preceding vertebrae. Progressing along the series shows this changing as the ventral margin angles downward, resulting in a more wedge-shaped outline in side view. The ventral margin flattens from the slight arch seen in the preceding vertebrae, and unlike the pronounced arch seen in the corresponding vertebrae of *Hesperornis*. Neither the costal nor spinal processes are preserved on these vertebrae in either specimen of *Parahesperornis*. From the broken area on the centrum it appears the spinal processes are longer and more centrally located on the centrum than in the previous vertebrae, as is also seen in *Hesperornis*.

The ventral surfaces of these vertebrae are shaped like an elongate hourglass, with a slight indent toward the caudal end. As in the more anterior vertebrae, the ventral surface is grooved along its length. This is also observed in *Hesperornis* and perhaps in *Baptornis,* however the cervical vertebrae are poorly preserved for that animal. In *Baptornis* the ventral surface remains very narrow, much more so than in the corresponding vertebrae of hesperornithids. A vertebra belonging to this range has been assigned to *Pasquiaornis* (RSM P2626.15) [65] which is like *Baptornis* in having a very narrow constriction of the ventral surface.

Unlike the previous vertebrae, in caudal view, the articular facets of the postzygapophyses face ventrally and slightly laterally, a trend that will continue in the subsequent vertebrae. This is similar to the case in *Hesperornis* but possibly different from that of *Baptornis,* where the postzygapophyses are spread further apart, angling outward to a degree not seen until around the fifteenth cervical in *Hesperornis* and *Parahesperornis.* In *Parahesperornis,* the postzygapophyses of these vertebrae have rounded margins in dorsal view and are separated by a slight indentation, as in *Hesperornis*, while in *Pasquiaornis* the postzygapophyses are more broadly spread, but without the projection seen in *Baptornis.* The eighth cervical of *Parahesperornis* is the first in the series to have high projections on the lateral margins of the prezygapophyses, as opposed to the rounded surfaces in the preceding vertebrae. The corresponding region is not preserved in *Hesperornis*.

**Figure 34:**
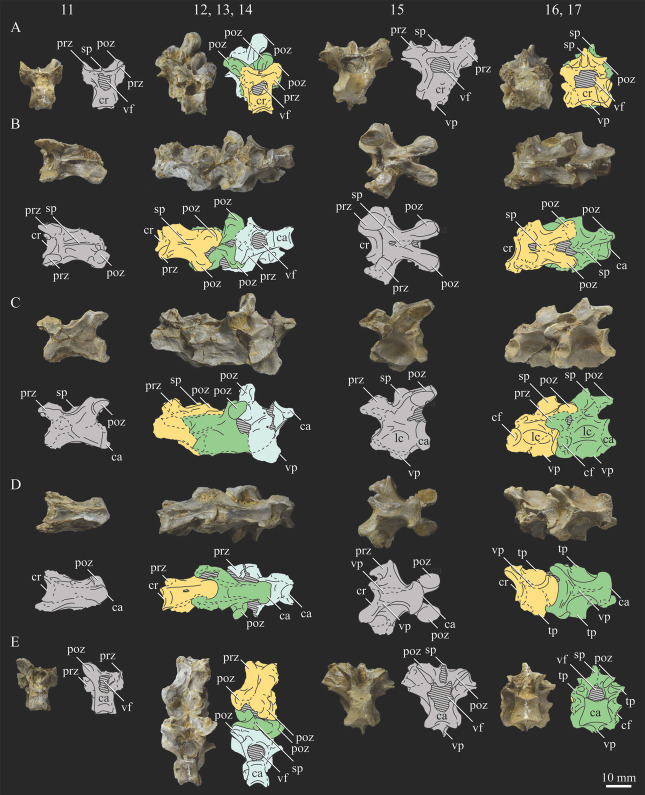
*Parahesperornis alexi* KUVP 2287, photographs and line drawings of cervicothoracic vertebrae: isolated eleventh (grey, 11); articulated twelfth (yellow), 13th (green), and 14th (light blue) (12, 13, 14); isolated 15th (grey, 15); and articulated 16th (yellow) and 17th (green) (16, 17) vertebrae in cranial (**A**), dorsal (**B**), lateral (**C**), ventral (**D**), and caudal (**E**) views. Hatch marks indicate areas obscured by matrix. Abbreviations are as follows: ca—caudal articular surface, cf—costal fovea, cr—cranial articular surface, lc—lateral concavity, poz—postzygapophysis, prz—prezygapophysis, sp—spinal process, tp—transverse process, vf—vertebral foramen, vp—ventral process.

**Figure 35:**
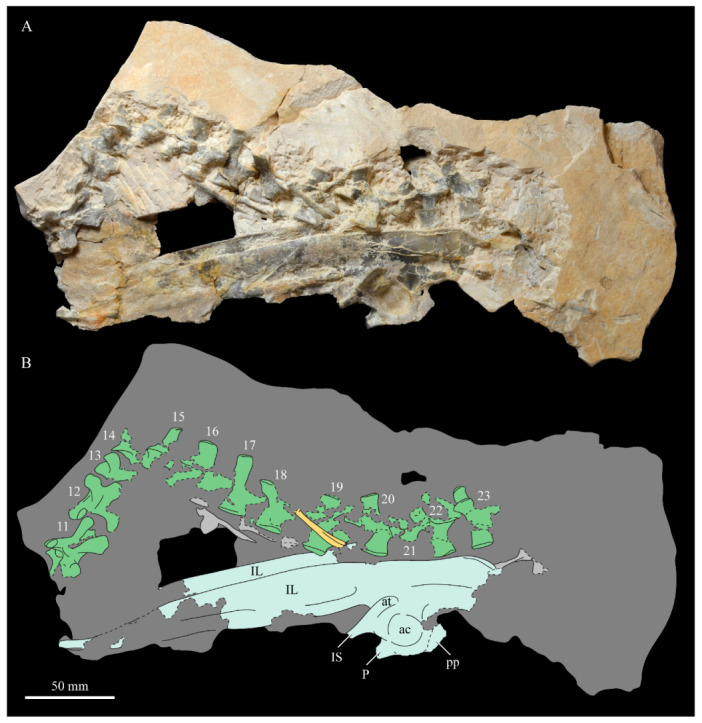
*Parahesperornis alexi* KUVP 24090, photograph (**A**) and line drawing (**B**) of the front of the slab containing vertebrae (green, numbered), pelvis (light blue), and ribs (yellow). Undetermined elements are light grey. Abbreviations: ac—acetabulum, at—antitrochanter, at—antitrochanter, IL—ilium, IS—ischium, P—pubis, pp—pectineal process.

**Figure 36:**
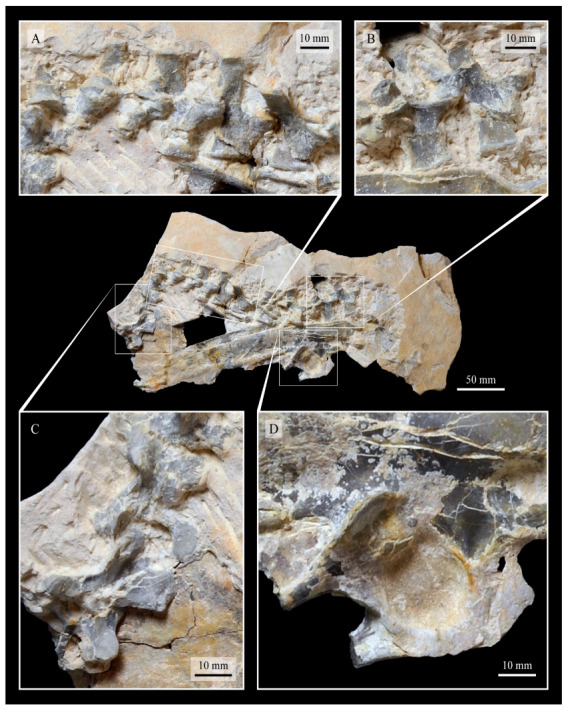
*Parahesperornis alexi* KUVP 24090, details of front of slab in Figure 35. Cervical vertebrae 14 to 18 (**A**), thoracic vertebrae 22 and 23 (**B**), cervical vertebrae 11 and 12 (**C**), and the acetabulum (**D**).

**Figure 37:**
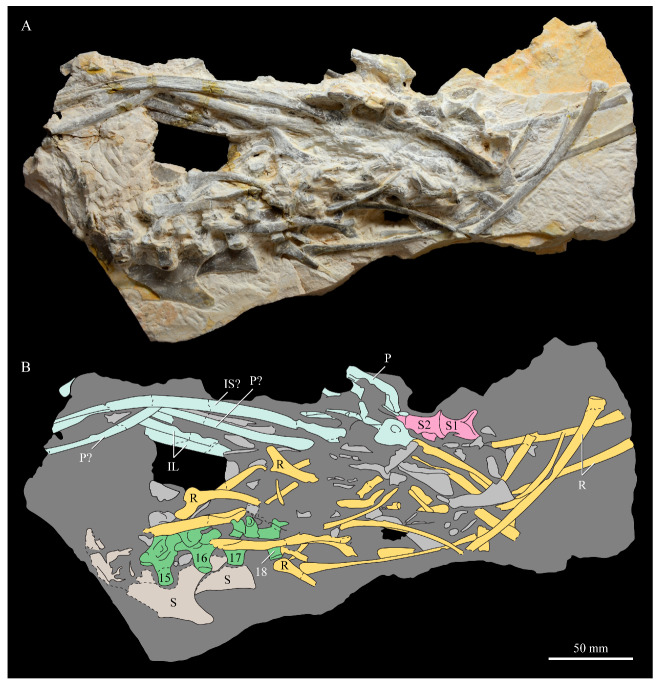
*Parahesperornis alexi* KUVP 24090, photograph (**A**) and line drawing (**B**) of the back of the slab in Figure 35 containing vertebrae (green), pelvis (light blue), synsacrum (pink), sternal fragments (tan), and ribs (yellow). Undetermined elements are light grey. Abbreviations: IL—ilium, IS—ischium, P—pubis, R—rib, S—sternum, S1—first centrum in the synsacrum, S2—second centrum in the synsacrum, 15–18—numbers of the thoracic vertebrae.

**Figure 38:**
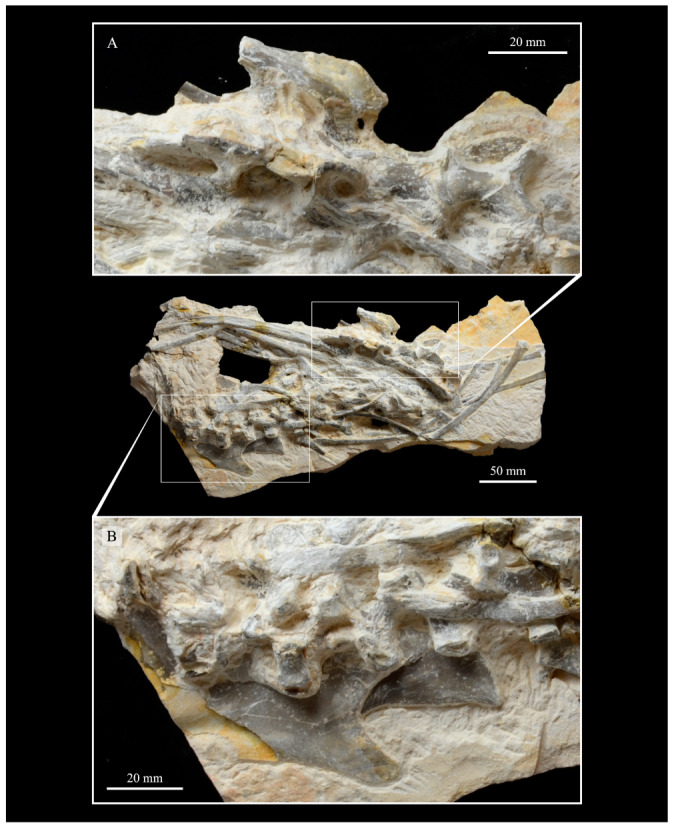
*Parahesperornis alexi* KUVP 24090, details of back of slab shown in Figure 37 showing synsacrum region of pelvis (**A**) and cervical vertebrae 15–18 (**B**).

**Cervical Vertebrae 10 to 13**. Within this series, *Parahesperornis* KUVP 2287 preserves the tenth cervical articulated to the ninth (Figure 32), the isolated eleventh, and the articulated twelfth, 13th, and 14th (Figure 34), while KUVP 24090 preserves the eleventh through 13th articulated in a slab (Figure 35, Figure 36, Figure 37 and Figure 38). The tenth to 13th cervical vertebrae of *Parahesperornis* are characterized by shortening of the centrum, as in *Hesperornis*, and the development of the postzygapophyses into widely spaced structures extending out from the centrum, a trend that is continued in the subsequent vertebrae. In *Hesperornis* these vertebrae are more compact than in *Parahesperornis.* A cervical vertebra proposed to be the twelfth, 13th, or 14th has been assigned to *Enaliornis* (YORYMG 507) [21]. Very little detail is preserved on that specimen; however, it is very compact and not elongated like those of other hesperornithiforms.

Cranially, the prezygapophyses are widely spaced and extend laterally past the edges of the cranial articular surface, which is rectangular. The articular facets of the prezygapophyses are angled to face dorsomedially, as in the preceding vertebrae. This is not seen in *Hesperornis*, where the articular facets face dorsally and are not angled. The cranial articular surfaces are narrower than in preceding vertebrae, as in *Hesperornis*. In lateral view, beginning with the eleventh cervical, the ventral margin arches dorsally, more like the state in the preceding vertebrae of *Hesperornis*. The sides of the centrum are nearly flat in the eleventh cervical but become broadly depressed in the subsequent vertebrae.

The ventral surfaces of these vertebrae are similar to those of the sixthto ninth cervicals, but do not narrow at the midlength. An isolated vertebra that corresponds most closely to the eleventh of hesperornithids has been reported for *Pasquiaornis* (RSM P2831.8) [65]. This vertebra is very similar in overall morphology, but more elongate and with a narrower ventral surface, including the cranial and caudal articular faces, as is also the case in the corresponding vertebrae of *Baptornis* (AMNH 5101).

The postzygapophyses are widely spaced and elevated above the height of the centrum, extending laterally from the caudal end of the centrum, as in *Hesperornis.* The postzygapophyses of *Baptornis* are exaggerated in this regard, extending dramatically from the end of the centrum on long necks. In dorsal view, the margins of the postzygapophyses are not smoothly rounded, as in the preceding vertebrae, but rather have more angular margins, a pattern which begins earlier, in the ninth cervical of *Hesperornis.* The postzygapophyses have peaked dorsal surfaces, as in *Hesperornis.* The peaks are more prominent and the postzygapophyses more elongate and widely spaced in *Baptornis.* The caudal articular surfaces jut outwards from the centrum, forming a shelf onto which the neural foramen opens. The articular surface is very narrow and curved, wrapping around the caudal end of the vertebrae and onto the sides to a greater degree than in the previous vertebrae, a trend continued in the subsequent vertebrae. This is also seen in *Hesperornis* and *Baptornis.*

**Cervical Vertebrae 14 to 15**. Within this series, *Parahesperornis* KUVP 2287 preserves the 14th vertebrae articulated to the twelfth and 13th and the isolated 15th (Figure 34), while the 14th and 15th vertebrae of KUVP 24090 are preserved articulated but unprepared (Figure 35 and Figure 36). The 14th and 15th cervical vertebrae of *Parahesperornis* are characterized by continued shortening of the centra. These are the first vertebrae to have transverse processes, as is also the case in *Hesperornis*. It is unclear whether the 14th cervical of *Baptornis* had transverse processes. Elaborate ventral processes are found in these vertebrae originating from the cranial end of the centrum. In *Parahesperornis* the 16th cervical has more in common morphologically with the subsequent vertebrae than with the preceding. This is very different from *Hesperornis,* where the 16th cervical shares the blocky, robust appearance of the 14th and 15th vertebrae, as well as possessing a short, robust ventral process. A 14th or 15th cervical vertebra has been assigned to *Asiahesperornis* (IZASK 2/KM 97) [80], which is morphologically similar to the cervical vertebrae of *Hesperornis* despite being closer in size to those of *Parahesperornis.*

The cranial articular surfaces of these vertebrae are much more robust than in either previous or subsequent vertebrae in the series, with thickened lateral margins that extend almost as wide as the prezygapophyses. In *Parahesperornis* and *Hesperornis* the ventral margins of the cranial articular surfaces form a broad arch, while in *Pasquiaornis* this arch is much narrower and deeper. The prezygapophyses are widely spaced with the articular surfaces facing dorsomedially, as in previous vertebrae. This is also seen in *Hesperornis*, whereas the orientation of the prezygapophyses in the preceding vertebrae was less angled. The neural spines are not preserved, but the scar indicates they were very short and caudally positioned on the centrum, as in *Hesperornis.* There is a deep, teardrop-shaped foramen present just caudal to the neural spine. This foramen is not seen in the corresponding cervicals of *Hesperornis* YPM 1206 but is present on the 15th cervical of *Canadaga*.

In lateral view, the 14th and 15th cervicals are the first vertebrae in the series with well-developed lateral concavities, as in *Hesperornis*. The lateral concavities of *Parahesperornis* are generally shallower than those of *Hesperornis*, while *Baptornis* has lateral concavities more deeply excavated than either hesperornithid. It should be noted that the excavation of the lateral concavity is uneven between the left and right sides of individual vertebrae in hesperornithiforms, as well as modern birds, making this feature of limited use in identification or diagnosis. A rudimentary facet that may be an articulation for a free rib is present on the cranioventral corner of the centrum in the 15th vertebrae, while the first well-developed rib articulation is present on the 16th cervical in *Parahesperornis*.

Beginning with the 14th vertebrae, the ventral surfaces are characterized by elaborate, often unique processes that are rarely preserved. While *Parahesperornis* KUVP 2287 does not preserve many of these processes, their general position can be determined by the pattern of breakage on the ventral surface. The corresponding vertebrae of *Baptornis* show similar evidence of breakage that may also indicate the presence of these processes. The ventral process of the 14th and 15th vertebrae differ from those of subsequent vertebrae by being very robust in their origin on the cranial end of the ventral surface and not as long, while the ventral processes of the thoracic vertebrae are very compressed mediolaterally and originate from the center of the ventral surface.

The 14th cervical of *Parahesperornis* has a broad, flaring surface extending from the ventral margins of the cranial articular surface that is broken along both margins. This paired breakage is suggestive of the pair of elongate, ventrally projecting flanges that protrude from the cranial end of the ventral surface of the centrum in *Hesperornis*. This morphology is unique to the 14th cervical of *Hesperornis,* making it readily identifiable. This is not the case in *Parahesperornis*, where the 15th cervical shows a similar pattern of breakage, suggesting a second vertebrae with paired ventral projections. In *Hesperornis*, however, the 15th cervical has a broad, thick ventral process descending from the central portion of the centrum and ending in a forked structure. While *Canadaga* NMC 41050 does not preserve the ventral processes completely, the remnant present in the 15th cervical resembles that of *Hesperornis*.

The ventral surfaces of the 14th and 15th vertebrae of *Parahesperornis* are the first in the series to have the exaggerated hourglass shape typical of the thoracic vertebrae of hesperornithiforms, with a narrow waist near the midline of the centrum and a flared, fan-shaped caudal margin. The waist is less pronounced in most hesperornithids than in *Baptornis.* The exception is *Canadaga*, a hesperornithid with extreme expansion of the cranial and caudal articular surfaces and a dramatic central waist in ventral view.

The postzygapophyses of these vertebrae have highly elongate necks that extend far past the centrum caudally, as is seen in earlier cervical vertebrae in *Baptornis*. The articular facets are ventrolaterally directed, with rounded caudal margins that do not extend past the margins of the neck, unlike in *Hesperornis* where the caudal ends are highly circular and expanded. In caudal view, the articular surface is narrow, with a waisted appearance and a flared ventral margin. The dorsal margin of the articular surface is narrower than that of *Hesperornis*.

**Figure 39:**
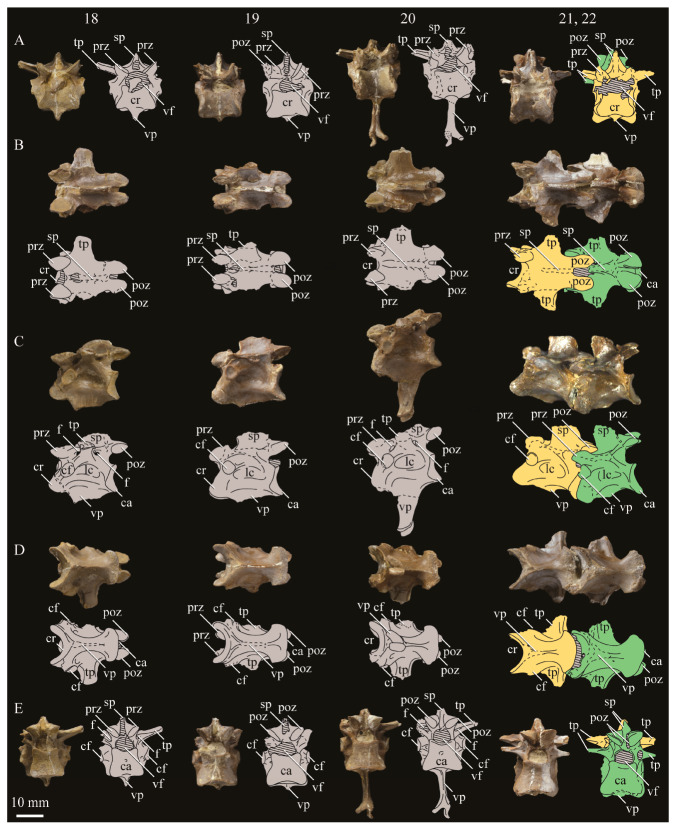
*Parahesperornis alexi* KUVP 2287, photographs and line drawings of thoracic vertebrae, from left to right: isolated 18th, 19th, and 20th (grey); articulated 21st (yellow) and 22nd (green) vertebrae in cranial (**A**), dorsal (**B**), lateral (**C**), ventral (**D**), and caudal (**E**) views. Abbreviations are as follows: ca—caudal articular surface, cf—costal fovea, cr—cranial articular surface, f—foramen. lc—lateral concavity, poz—postzygapophysis, prz—prezygapophysis, sp—spinal process, tp—transverse process, vf—vertebral foramen, vp—ventral process.

**Cervical vertebra 16 to 17 and thoracic vertebrae 18 to 23.** Marsh [7] began the series of thoracic vertebrae with the 18th vertebra, as it was the first vertebra to articulate with the sternum through a sternal rib. Despite this, both *Hesperornis* (the 17th vertebra) and *Parahesperornis* (the 16th and 17th) have preceding vertebrae that bear a closer overall resemblance to the 18th vertebrae (Figure 39), and so these vertebrae are discussed together.

The appearance of the 16th vertebra of *Parahesperornis* represents a transition along the vertebral column. The anterior cervical vertebrae of hesperornithids, particularly eleven through 15, each show unique features that make identifying them relatively easy as compared to the thoracic vertebrae, which are more similar. These features include things like the elaborate ventral processes and the shapes of the postzygapophyses in both dorsal and lateral views, as described above. The 16th vertebrae of *Parahesperornis* is interesting in that it clearly shows the beginnings of a number of features common among the subsequent vertebra, but not as developed. This includes features such as comparatively shorter and caudally-directed postzygapophyses, the sides of the centrum less waisted in ventral view than in the previous vertebrae, and the loss of the robust lateral expansions of the ventral half of the cranial articular surface. In *Hesperornis,* this sort of transitional appearance is instead found in the 17th vertebra.

**Figure 40:**
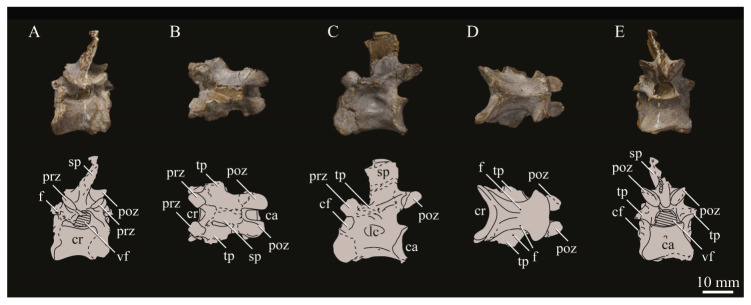
*Parahesperornis alexi* KUVP 2287, photographs (upper) and line drawings (lower) of the 23rd vertebra in cranial (**A**), dorsal (**B**), lateral (**C**), ventral (**D**), and caudal (**E**) views. Abbreviations are as follows: ca—caudal articular surface, cf—costal fovea, cr—cranial articular surface, f—foramen. lc—lateral concavity, poz—postzygapophysis, prz—prezygapophysis, sp—spinal process, tp—transverse process, vf—vertebral foramen.

As described above, the 16th to 23rd vertebrae of *Parahesperornis* are remarkably similar in form (Figure 38, Figure 39 and Figure 40), a departure from the previous vertebrae which were more variable (Figure 32, Figure 33 and Figure 34). This similarity makes definitive placement within the series difficult unless articulated. While KUVP 24090 preserves the articulated cervicothoracic series, the chalk they are preserved in obscures many details of morphology necessary for specific vertebral identification (Figure 35 and Figure 37). For the disarticulated KUVP 2287, identifications were made through the fit of articulations as well as small morphological differences, in particular, the placement of the 18th through 20th vertebrae is tentative.

Two fairly well-preserved thoracic vertebrae have been assigned to *Pasquiaornis* (RSMP P2957.15, and an additional un-numbered vertebra [65]). They have been described as “slightly amphicoelus” [65], which one specimen appears to be (RSM P2957.15) [65]. Another specimen (incorrectly labeled as RSM P2957.15 [65]), however, is heterocoelus. They appear to be highly pneumatic as well, with numerous foramina on the centra around the transverse processes and toward the caudal margin. Breakage along the spinal and transverse processes shows extensive pneumatization within the bone, more so than is seen in corresponding areas of breakage in hesperornithids. Thoracic vertebrae are also known for *Brodavis varneri* (SDSM 6843) [31], however they are heavily encrusted in gypsum and very little can be said about the morphology other than that they seem to agree with other hesperornithiforms. *Chupkaornis* preserves a vertebra identified as the 16th [12], which is poorly preserved but agrees in general appearance with that of *Parahesperornis*.

The 16th to 23rd vertebrae of *Parahesperornis* are characterized by shortened and oval-shaped postzygapophyses; tall and centrally-located spinal processes; deep lateral concavities on the centra; progressively dorsally-displaced costal fovea; cranial and caudal articular surfaces expanded, but not as much as in the previous posterior cervical vertebrae; centrally-located transverse processes with deep depressions on the cranial and caudal faces; narrow and centrally-placed ventral processes that decrease in size progressively in the series; and are fully heterocoelous. Overall, this is very similar to the thoracic vertebrae of *Hesperornis* and *Baptornis*. As noted earlier, the degree of excavation of the lateral concavities is highly variable within species and even individuals. Some specimens assigned to *Baptornis* have very faint lateral concavities on the centra of the thoracic vertebrae (AMNH 5101, SDSM 5893), while in others the concavities are developed to a similar degree as seen in hesperornithids (KUVP 2280, KUVP 2290). A single well-preserved anterior thoracic vertebra assigned to *Pasquiaornis* (RSM P2988.12) [65] has deeply excavated lateral concavities, as does *Canadaga*. The preserved 15th–18th vertebrae of *Canadaga* (NMC 41050, NUVF 284) show extreme excavation of the lateral concavities, almost to the extent of looking like large fossae.

The spinal processes of these vertebrae originate at the caudal end of the centrum and become progressively longer along the series. The spinal process of the 16th vertebrae occupies roughly half the length of the centrum, while that of the 23rd is almost twice as long. This is also the case in *Hesperornis*, however in *Baptornis* and *Pasquiaornis* the spinal processes occupy most of the dorsal surface of the centrum in the preserved thoracic vertebrae. Little can be said about the height of the spinal processes; however, that of the 23rd is nearly complete and extends very straight dorsally to a height roughly equivalent to that of the centrum (Figure 40). In *Hesperornis* the spinal process of the 23rd vertebrae is even taller, exceeding the height of the centrum.

The transverse processes are not well preserved in any of these vertebrae of *Parahesperornis*. They are located slightly toward the cranial end of the centrum and become narrower along the series, as in *Hesperornis*. The origination of the transverse process on the centrum bears deep triangular fossae on both the cranial and caudal sides. In *Hesperornis*, deep grooves are present in the same position but extending out along the transverse process. In *Baptornis,* these fossae are limited to the sides of the centrum, and do not extend onto the transverse processes. The preserved portions of the transverse processes are oriented directly laterally in *Parahesperornis*. This is similar in *Hesperornis* for the more anterior thoracics, but those of the 22nd and 23rd are oriented slightly cranially.

In *Parahesperornis*, the 16th is the first vertebra in the series with a well-developed, oval fossa for an articulation to a free rib. This fossa is located on the cranioventral margin of the lateral side of the centrum and is slightly angled to face cranially. This fossa is well-developed in the corresponding vertebra of both *Hesperornis* and *Baptornis*, however in both of those taxa it is oriented directly laterally. The costal foveae of the subsequent vertebrae in *Parahesperornis* are directed laterally, as in other hesperornithiforms. The placement of the costal foveae on the centra changes along the series, migrating dorsally such that in the 22nd vertebra it is nearly at the height of the transverse process. The shape also changes, being oval in most of the vertebrae but circular in the 19th to 21st. These trends are also seen in *Hesperornis.* In *Baptornis* the position of the costal foveae changes similarly, however they are more consistently circular in that taxon. The 23rd vertebra has only a faint oval face and not a well-developed costal fovea, as in *Hesperornis* and *Baptornis.*

While most of the ventral processes are not preserved in KUVP 2287, the scar along the ventral surface indicates they were very narrow and centrally located, unlike the previous ventral processes that were offset cranially and robust. Like in *Hesperornis*, the ventral processes took up most of the ventral surface of the centra in the more cranial thoracic vertebrae, but they become craniocaudally shorter along the series. In *Parahesperornis* the reduction of the ventral processes is greater than in the corresponding vertebrae of *Hesperornis.* This shortening of the ventral process is present only in the 22nd vertebra of *Baptornis*, while the 21st appears to have had the same width of process as the preceding thoracic vertebrae. Ventral processes appear to only be present on the more cranial thoracic vertebrae of *Pasquiaornis* [65]. However, definitive identification of the isolated specimens is not possible.

Only the complete ventral process of the 20th vertebra is known for *Parahesperornis.* It extends ventrally to a length approximately equal to the height of the centrum and has a forked end that has been somewhat crushed. *Hesperornis* UNSM 4-19-5-36 has an identical ventral process on the corresponding vertebra. It should be noted that the articulated vertebral column of KUVP 24090 may well preserve these processes, however without preparation these features cannot be studied. The ventral surfaces of these vertebrae continue the hourglass outline typical of hesperornithiforms and seen in the 14th and 15th vertebrae. This shape becomes more symmetrical along the series, with the fan-shaped expansion of the caudal end being almost as wide as that of the cranial end.

The 23rd vertebra is unique among the thoracics in not having a ventral process, as is also the condition in other hesperornithiforms. This vertebra has also been reported for *Enaliornis* (SMC B55277) [21]. While the identification as the 23rd is not definitive for an isolated specimen, it does lack a ventral process. It is also clearly heterocoelus [16,21], contra Tanaka et al. [12]

The postzygapophyses of these vertebrae are in general closely spaced, short, and oriented directly caudally. Those of the 16th are somewhat intermediate between the elongate postzygapophyses of the previous vertebrae and the reduced form seen in the latter. The caudal articular surfaces are robust and indented on all margins, being narrower on the dorsal margin than the ventral. This discrepancy in widths increases along the series. In cranial view the thoracics of *Parahesperornis* and very similar to those of *Hesperornis.*

**Figure 41:**
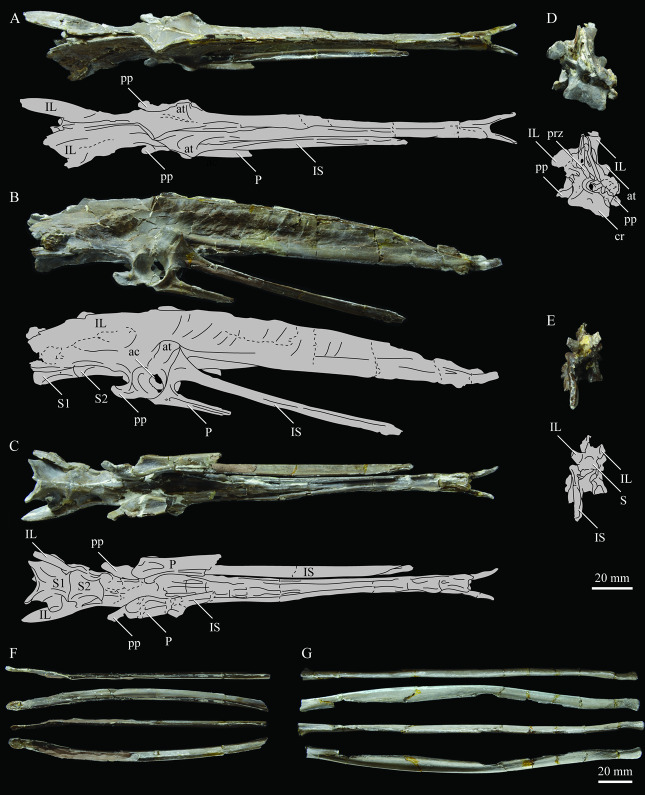
*Parahesperornis alexi* KUVP 2287, photographs (upper) and line drawings (lower) of synsacrum and pelvis in dorsal (**A**), left lateral (**B**), ventral (**C**), cranial (**D**), and caudal (**E**) views; fragments may represent portions of the pubis (**F**) or ischium (**G**). Abbreviations: ac—acetabulum, at—antitrochanter, cr—cranial articular surface, IL—ilium, IS—ischium, P—pubis, pp—pectineal process, prz—prezygapophysis, S1—first centrum in the synsacrum, S2—second centrum in the synsacrum, S—synsacrum.

**Synsacrum**. The synsacrum of *Parahesperornis* KUVP 2287 is preserved in articulation within the pelvis, and thus most anatomical features of this compound bone are obscured (Figure 41). It appears to contain eleven sutured or fused centra, however breakage makes this number difficult to confirm. The sacrum of KUVP 24090 is preserved primarily within the pelvis and encased in matrix. On one side of the slab, the first two sutured centra are exposed (Figure 37 and Figure 38). These are identified as belonging to the sacral vertebrae, and not to the caudal-most thoracic vertebrae, by the completely smooth ventral surfaces, lacking even the faint ventral crest visible on the 23rd thoracic vertebra of *Parahesperornis*. Marsh [7] described the synsacrum of *Hesperornis* YPM 1207 as having fourteen centra in the ankylosed section, more than in *Parahesperornis*, however the degree of fusion in YPM 1207 makes this assertion difficult to confirm. Lucas [13] and Martin and Tate [19] both reported the number of centra in the synsacrum of *Baptornis* (AMNH 5101) as ten; however, due to crushing this is also difficult to confirm. In other hesperornithiforms preserving the synsacrum, such as *Enaliornis* and *Brodavis varneri*, this compound bone is not complete.

The vertebrae incorporated into the synsacrum of *Parahesperornis* increase in the degree of joining along the series, with the first and second sutured and their margins clearly delineated, while further along the synsacrum the vertebrae are fully fused, with margins obscured. This is also seen in *Hesperornis* and *Pasquiaornis.* However, in *Baptornis,* the cranial centra are fully fused, with no discernable boundaries between them, while the caudal centra appear to be sutured and not fully fused. The first centrum in the synsacrum of *Parahesperornis* KUVP 2287 is crushed dorsally, obscuring the neural spine. The cranial articular surface is broad, shallowly excavated, and rectangular in outline. In dorsal view the centrum is similar to that of the 23rd vertebra. However, the caudal articular surface is much narrower than the cranial. This appears to also be the case in the second centrum. However, the states in the other centra are difficult to determine due to the degree of fusion. In *Baptornis*, the cranial articular end of the first centrum in the synsacrum is only slightly wider than the caudal end in dorsal view (as determined from the indentation in the sides of the centrum in dorsal view, as the exact caudal margin is fused and not visible).

A very short, somewhat stubby transverse process is preserved on the right side of the first centrum in KUVP 2287. A reduced transverse process is also present on the first centrum of the synsacrum in *Hesperornis.* Dorsally, the width of the synsacrum narrows dramatically from around the level of the acetabulum caudally, as in *Hesperornis,* while in *Baptornis* the caudal synsacrum does not become mediolaterally restricted until well past the level of the acetabulum. Overall, the portions of the synsacrum of *Parahesperornis* KUVP 2287 that are visible suggest a great similarity with this compound bone in *Hesperornis*.

**Figure 42:**
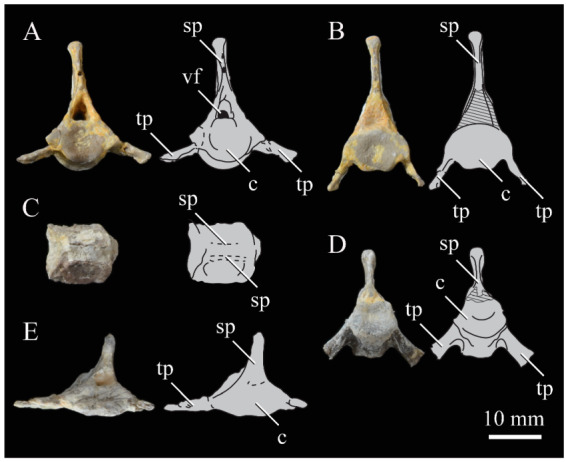
*Parahesperornis alexi* KUVP 2287, isolated free caudal vertebrae (**A**–**E**). Abbreviations: c—centrum, sp—spinal process, tp—transverse process, vf—vertebral foramen. Hatches indicate matrix.

**Figure 43:**
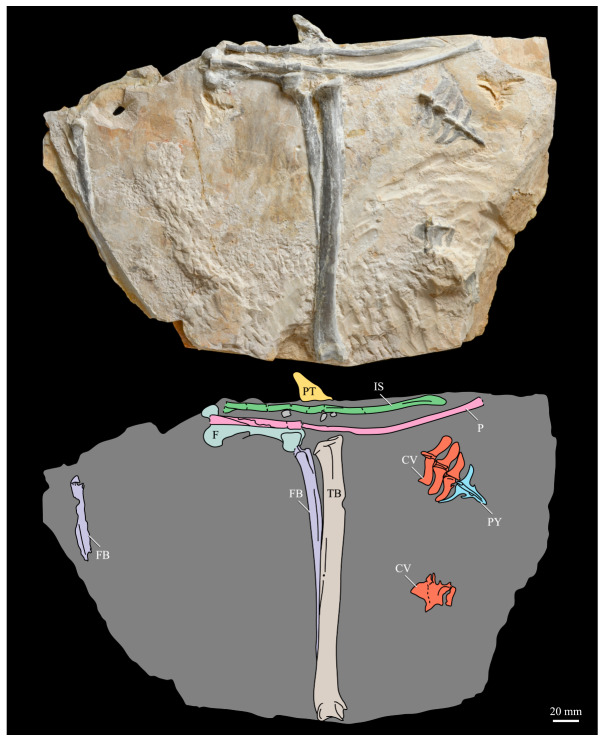
*Parahesperornis alexi* KUVP 24090, photographs (upper) and line drawing (lower) of slab containing ischium (green, IS), pubis (pink, P), patella (yellow, PT), femur (pale blue, F), fibulae (purple, FB), tibiotarsus (tan, TB), free caudal vertebrae (orange, CV), and pygostyle (blue, PY).

**Figure 44:**
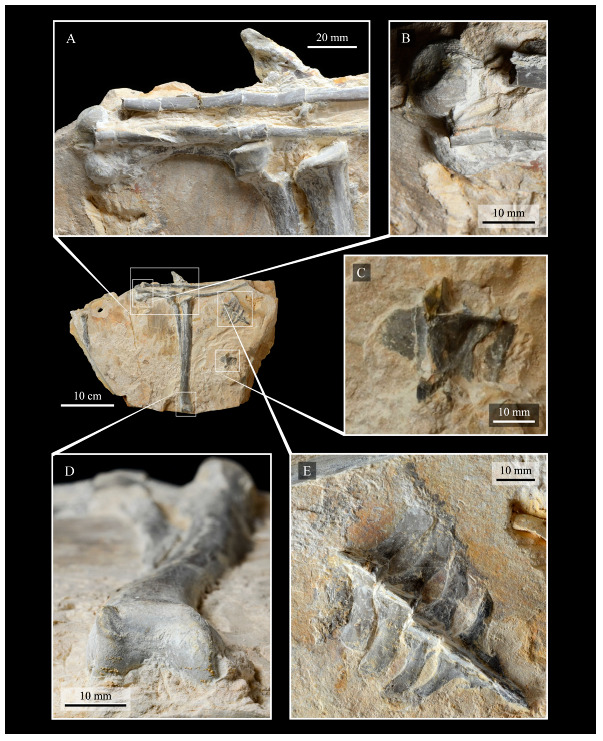
*Parahesperornis alexi* KUVP 24090, details of slab shown in Figure 43: femur underlying proximal ischium and pubis with proximal patella, fibula, and tibiotarsus (**A**), head of femur showing fovea for the capitol ligament (**B**), isolated caudal vertebra (**C**), distal tibiotarsus (**D**), and articulated caudal vertebrae and pygostyle (**E**).

**Caudal vertebrae.** *Parahesperornis* KUVP 2287 preserves four isolated free caudal vertebrae (Figure 42), while KUVP 24090 preserves one isolated and three articulated free caudal vertebrae (Figure 43 and Figure 44). For comparison, the caudal vertebrae of *Hesperornis* YPM 1200 were examined. The caudal vertebrae of *Parahesperornis* possess extremely wide transverse processes that curve slightly cranially in dorsal view. While the width is similar between *Parahesperornis* and *Hesperornis,* the transverse processes of *Hesperornis* do not curve, but instead extend straight but at an angle cranially from the centrum. The caudal-most vertebrae have thinner and progressively shorter transverse processes, despite having only slightly shorter centrum lengths.

None of the spinal processes are preserved in KUVP 24090, however some of the free caudal vertebrae of KUVP 2287 preserve very tall spinal processes. The most complete caudal series of *Hesperornis* consists of ten free caudal vertebrae preserved in YPM 1200; however, it cannot be determined if the caudal series of this specimen is complete, as the individual elements are disarticulated. Given the overall similarities of *Parahesperornis* caudal vertebrae with those of *Hesperornis*, it is reasonable to posit that *Parahesperornis* may have had at least ten caudal vertebrae, the same as in *Hesperornis*.

**Pygostyle.** The complete pygostyle is preserved in *Parahesperornis* KUVP 24090 in dorsal view in a slab containing part of the pelvis and hindlimb (Figure 43 and Figure 44) and is unknown for KUVP 2287. A partial pygostyle preserving two full centra and a partial third is known from *Hesperornis* (YPM 1200) (as described by Marsh [7] and contra Martin and Tate [19]), and another preserving three centra is known from *Fumicollis* (UNSM 20030). Martin and Tate [19] described the pygostyle of *Baptornis* on the basis of UNSM 20030, which has since been reassigned as the holotype of *Fumicollis* [20], and FNMH 395, which does not include a pygostyle today. It is therefore not possible to confirm Martin and Tate’s assertion [19] of five fused centra in the pygostgyle of *Baptornis*.

The pygostyle of *Parahesperornis* preserves three fused centra. The first centrum has moderate transverse processes, while those on the second are rudimentary and those on the third are absent (though possibly broken). A dorsal ridge is present along the center of the fused centra. The transverse processes of the first centrum of the pygostyle of *Hesperornis* appear to be proportionally of the same length as those of *Parahesperornis*, however much more robust, while those of the second centrum are only partially preserved. Similarities among these elements indicate that the pygostyle of more derived hesperornithiforms was small and formed by just a few vertebrae.

#### 3.2.3. Thoracic Girdle

**Figure 45:**
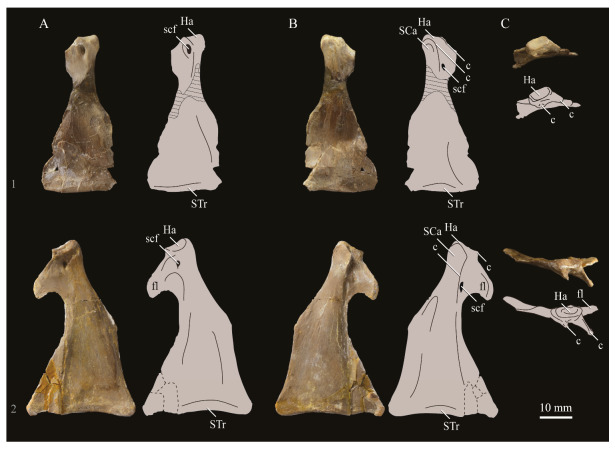
*Parahesperornis alexi* KUVP 2287 (1) and KUVP 24090 (2), photographs (left) and line drawings (right) of right coracoids in ventral (**A**), dorsal (**B**), and proximal (**C**) views. Hatchmarks on the line drawings indicate putty. It should be noted that a portion of the entire neck of KUVP 2287 is missing, causing difference in the orientation of the omal ends between the two coracoids. Abbreviations: c—crest, fl—flange, Ha—humeral articular facet, SCa—scapular articular facet, scf - supracoracoideous nerve foramen, STr—sternal ridge.

**Figure 46:**
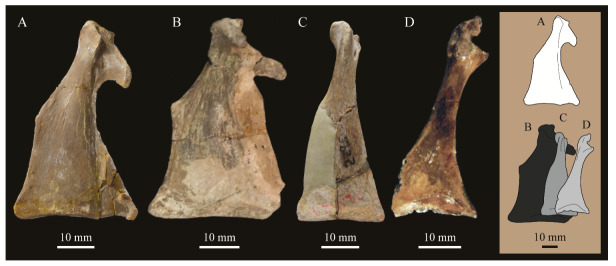
Comparison of the coracoids of *Parahesperornis* KUVP 24090 (**A**), *Hesperornis* FHSM VP- 2069 (**B**), *Baptornis* KUVP 2290 (**C**), and *Pasquiaornis* RSM P2988.9 [65] (**D**) in dorsal view. Elements are scaled to be of a similar length and aligned at the proximal ends. Inset shows silhouettes of the same elements to scale.

**Coracoid.** The right coracoid of *Parahesperornis* is preserved in both KUVP 2287 and KUVP 24090 (Figure 45). The coracoid of KUVP 24090 is completely preserved, but that of KUVP 2287 is missing part of the neck and has been reconstructed. The striking difference in the angle of the omal end of the coracoids of KUVP 2287 and KUVP 24090 is an artifact of the reconstruction of KUVP 2287, which was incorrectly reconstructed. For comparison, the coracoids of *Hesperornis* (YPM 1207, FHSM VP-2069), *Baptornis* (KUVP 2290), and *Pasquiaornis* (RSM P2988.9) [65] were examined (Figure 46).

The coracoid of *Parahesperornis* is a very thin, elongate bone shaped roughly like a triangle in dorsal or ventral view. In *Baptornis* and *Pasquiaornis* the coracoid is more elongate, with less expansion at the sternal end, while in *Hesperornis* it is more robust. Just over halfway along the length, the body narrows sharply into a neck that angles proximomedially before flaring out again at the omal extremity. The length of the neck of *Parahesperornis* is most similar to that of *Baptornis*, intermediate between the very long neck of *Pasquiaornis* and the incredibly reduced, almost absent, neck of *Hesperornis.* The neck of *Parahesperornis* is angled medially, with the omal end offset over the medial margin of the body. This is very different from *Hesperornis* and *Baptornis*, where the omal end is more centered over the body of the coracoid. However, in *Pasquiaornis* the omal end is also angled slightly medially.

The omal end is twisted slightly and set at an angle with respect to the main body of the coracoid, with a pair of ridges running from the slight acrocoracoid process to a large dorsomedially-projecting flange. This flange was proposed by Marsh [7] as supporting the clavicle, which is reasonable, however that articular relationship is not preserved in any specimen. While not preserved on any of the coracoids of *Baptornis,* this flange is also present in *Hesperornis*, where it appears to be proportionally larger. Below the omal end and adjacent to the large flange, a large foramen penetrates the ventral surface of the bone, probably synonymous with the supracoracoideous nerve foramen, and emerges distally on the dorsal neck of the coracoid between the crests defining the omal end. This foramen is notably larger in KUVP 2287 than in KUVP 24090 (Figure 45). While a similarly placed foramen is also seen in the other hesperornithiform coracoids, its size in *Parahesperornis* is unusual as compared to the smaller, more typical foramina in the other hesperornithiform taxa.

The articular facets on the omal extremity of the coracoid are incredibly faint and poorly developed in *Parahesperornis*, as in *Hesperornis* and *Baptornis*. *Pasquiaornis* has better-developed humeral and scapular articular facets. In this taxon, the humeral articular facet is rotated to face craniomedially, while that of the scapula is located below the humeral articular facet on the dorsal face of the omal extremity.

On the dorsal surface the body of the coracoid of *Parahesperornis* is smooth and slightly rounded, with a shallow depression along the lateral margin, while the ventral surface is nearly flat. The sternal margin of the coracoid is somewhat thickened and gently arched, as in *Hesperornis.* In *Baptornis* the sternal margin is only very slightly arched and shows a much fainter ridge than in hesperornithids. *Pasquiaornis* has a clear sternal articular facet developed on the dorsal face of the sternal margin of the coracoid.

**Figure 47:**
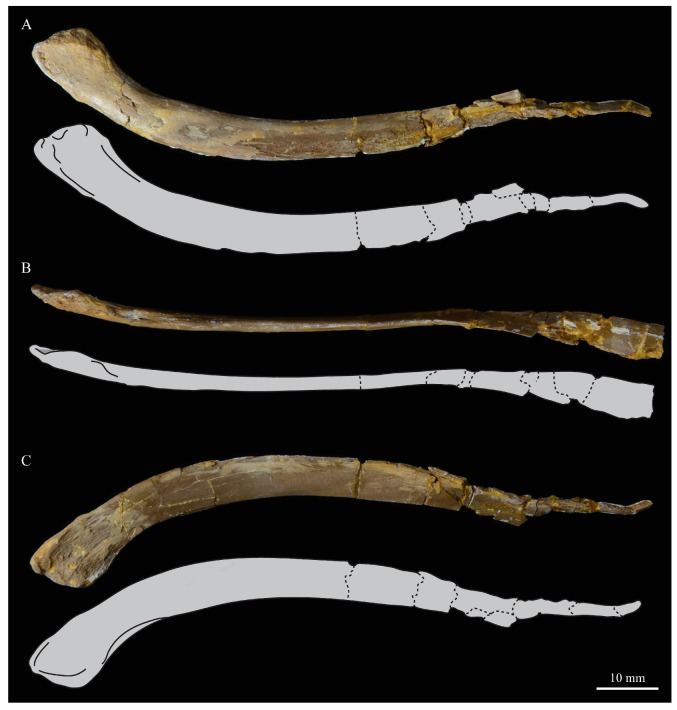
*Parahesperornis alexi* KUVP 2287, photographs (upper) and line drawings (lower) of the right clavicle in cranial (**A**), dorsal (**B**), and caudal (**C**) views.

**Clavicle.** The left clavicle of *Parahesperornis* is preserved in KUVP 2287 (Figure 47). The clavicles of *Parahesperornis* were unfused, as in *Hesperornis.* The clavicle narrows gradually along its length toward the omal end, which is not preserved. A pronounced curve is present along the shaft; this curvature is deepest near the symphyseal end, which is broad and flat. This bone is similar in morphology to the clavicle of *Hesperornis*, as figured by Marsh [7], however the clavicle was not available for examination from of the specimens with which it is preserved at the time of this study (YPM 1206, 1207, 1474). The clavicle is unknown for other species of hesperornithiforms.

**Figure 48:**
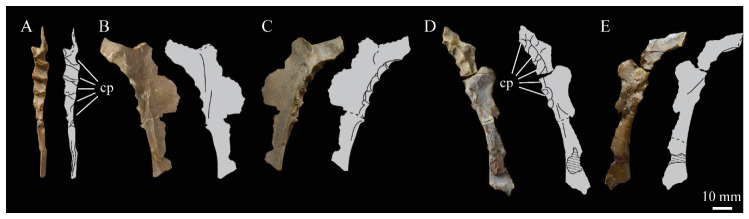
*Parahesperornis alexi* KUVP 2287, photographs (left) and line drawings (right) of two sternal fragments (**A**–**C** and **D**–**E**) in lateral (**A**), dorsal (**B**), ventral (**C**,**E**), and laterodorsal (**D**) views. Abbreviation: cp—costal processes.

**Figure 49:**
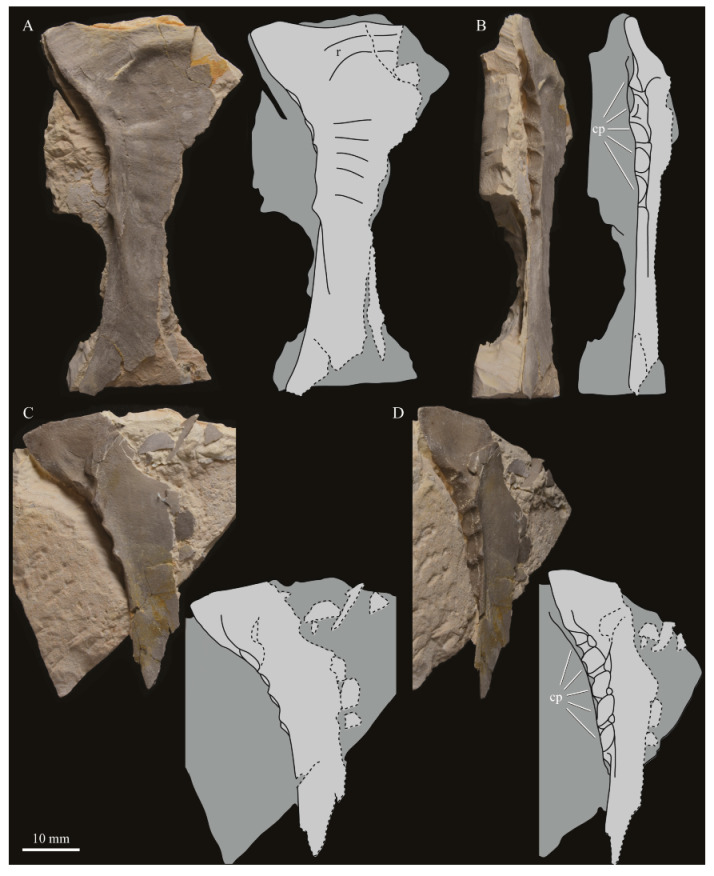
*Parahesperornis alexi* KUVP 24090, photographs (left) and line drawings (right) of left (**A**,**B**) and right (**C**,**D**) halves of sternum in ventral (**A**), dorsal (**C**), and lateral (**B**,**D**) views. Abbreviations: cp—costal processes, r—ridge.

**Sternum**. The sternum of *Parahesperornis* is preserved in KUVP 2287 as small portions of both sides, each from about the same level and preserving five costal processes (Figure 48). KUVP 24090 preserves two large portions of the sternum still embedded in chalk, almost the entire left half in ventral view and a large portion of the right half in dorsal view (Figure 49), as well as other less visible portions in a slab with the vertebrae and pelvis (Figure 35 and Figure 37). For comparison, sterna of *Hesperornis* (YPM PU 18589, YPM 1476, FHSM VP-2069) were examined. The best preserved *Hesperornis* sternum is that of the holotype of *H. crassipes* (YPM 1474). However, at the time of this study that specimen is on display and not accessible. While Martin and Tate [19] reported and figured a sternum for UNSM 20030, now the holotype of *Fumicollis hoffmani,* a sternum was not present with that specimen neither when this species was described [20] nor at the time of this study. The sternum is unknown for other species of hesperornithiforms.

The sternum of *Parahesperornis* is broad and flat, with a wide cranial margin, a narrow body, and a flared caudal end. Unfortunately, neither specimen preserves the midline of the sternum, making it impossible to assess if the left and right sides were fused or not, and to determine the size of the carina (if present). The cranial margin of the sternum had an undulating outline, with a subtle rostral projection near the middle, and a gentle dip that flared cranially at the outer margins. A large ridge is present on the ventral surface running parallel to the cranial margin, near the middle of the cranial margin. The preserved dorsal surface of the right half of the sternum is flat and featureless, with five costal processes along the outer margin. The sternum is thickest at the level of the costal processes and thins cranially and caudally.

Marsh [7] described *H. regalis* as having four costal processes and *H. crassipes* as having five. Both *H. crassipes* (YPM 1474) and the sternum of *H. regalis* described by Marsh (YPM 1206) are on display at the Yale Peabody Museum and were not available for detailed examination at the time of this study. However, the sternal fragments preserved with YPM PU 18589, another specimen of *Hesperornis*, have five costal processes, as in *Parahesperornis*. Marsh also described the sternum of *H. crassipes* (YPM 1474) as possessing deep grooves on the ventral surface near the cranial margin for articulation of the coracoids, but such grooves are not present in *Parahesperornis.*

**Figure 50:**
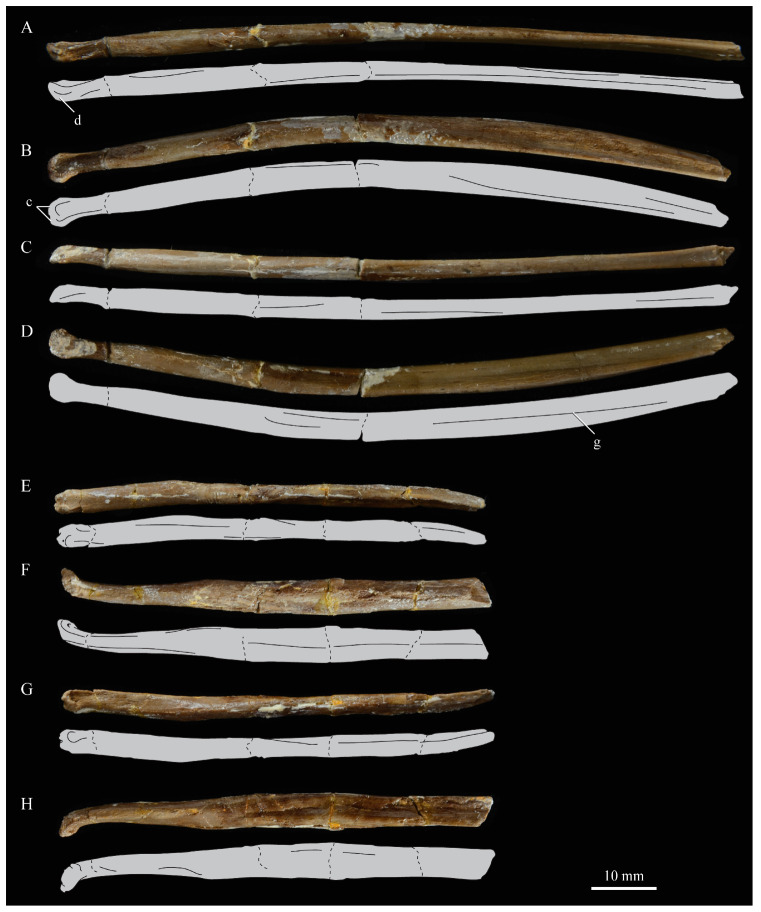
*Parahesperornis alexi* KUVP 2287, photographs (upper) and line drawings (lower) of right (**A**–**D**) and left (**E**–**H**) humeri in dorsal (**A**,**E**), cranial (**B**,**F**), ventral (**C**,**G**), and caudal (**D**,**H**) views. Abbreviations: c—condyles, d—deltopectoral crest, g—groove.

**Figure 51:**
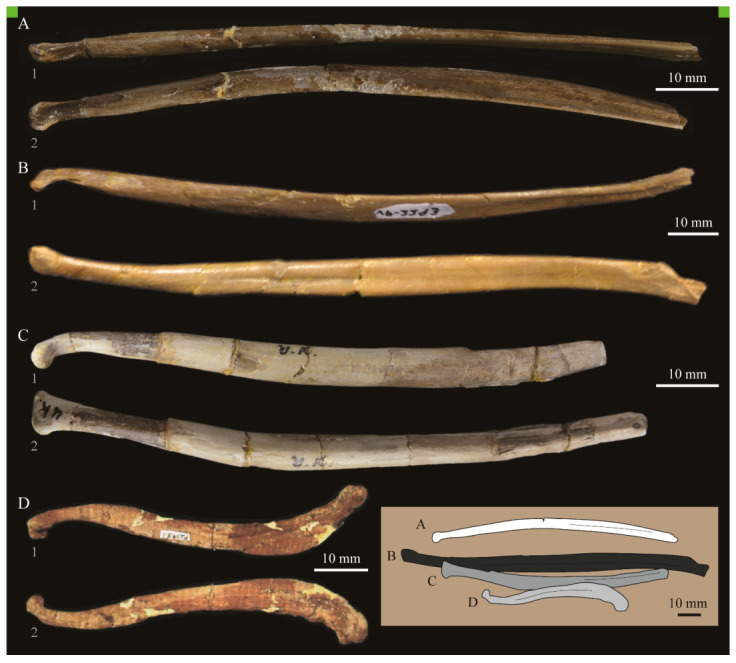
Comparison of the humeri of *Parahesperornis* KUVP 2287 (**A**), *Hesperornis* FHSM VP-2293 (**B**), *Baptornis* KUVP 2290 (**C**), and *Pasquiaornis* RSM P2995.1 [65] (**D**) in dorsal (1) and cranial (2) views (cranio-ventral for *Pasquiaornis*, D2). Elements are scaled to be of a similar width across the distal condyles and aligned at the distal ends. Inset shows silhouettes of the same elements to scale.

**Humerus.** *Parahesperornis* KUVP 2287 preserves the distal ends of both humeri (Figure 50). For comparison, the humeri of *Hesperornis* (FHSM VP- 2293), *Baptornis* (KUVP 2290), and *Pasquiaornis* (casts of RSM P2077.4 and RSM P2487.3 at the YPM) were examined (Figure 51). The humerus of *Parahesperornis* is a very slender bone, with a thin shaft and slightly wider, rounded distal end. The shaft is arched dorsally with a groove running along the caudal surface that is also present in *Hesperornis*. The left humerus is straighter than the right, possibly due to crushing along the shaft. The humeri of *Hesperornis* and *Baptornis* are also arched, although not to the degree seen in *Parahesperornis*. The distal articular end of the humerus is nearly smooth, with condyles only faintly suggested on the cranial surface of the right humerus, and not preserved at all on the left. *Hesperornis* also lacks well-developed humeral condyles. In *Baptornis,* faint lateral and medial condyles, as well as a brachial fossa, can be seen on the cranial surface of the distal humerus. In *Parahesperornis*, the ventral face of the articular end is slightly depressed. The articular end hooks caudally, as is also the case in *Hesperornis* and *Baptornis*.

Overall, the preserved morphology of the humerus of *Parahesperornis* is like that of *Hesperornis* and *Baptornis.* All three of these taxa show extreme adaptations consistent with the loss of flight, while *Pasquiaornis* humeri do not show this degree of reduction. The shaft of the humerus of *Pasquiaornis* is more robust overall and forms an s-curve with a slight deltopectoral crest developed proximally and a faint differentiation between the head and dorsal tubercle. While the humerus of *Pasquiaornis* is thus more similar to those of flighted birds in some regards, it still exhibits an overall reduction in form as compared to volant birds.

#### 3.2.4. Ribs

**Figure 52:**
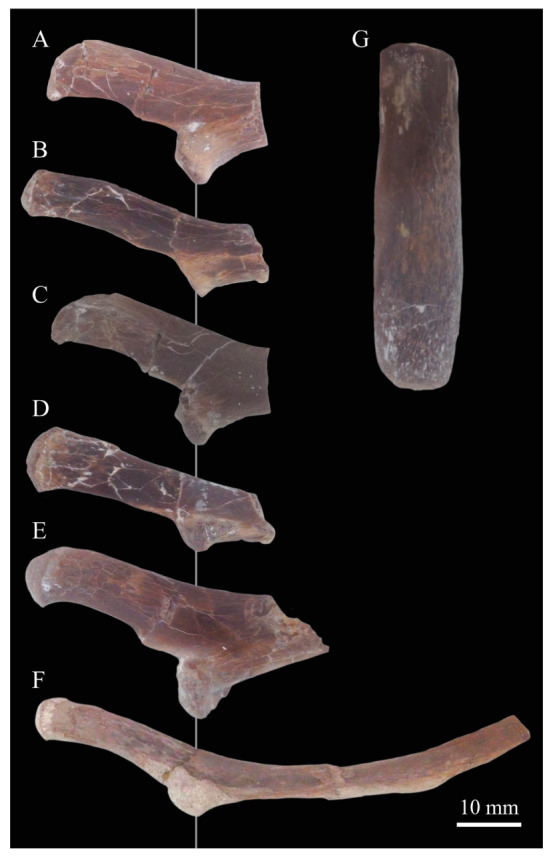
*Parahesperornis alexi* KUVP 2287, fragments of rib heads (**A**–**F**) and an uncinate process (**G**).

*Parahesperonis* KUVP 2287 preserves several rib fragments, although all are highly fragmentary (Figure 52). In KUVP 24090 many ribs are better preserved in the slab containing the vertebrae (Figure 37), however most are obscured by matrix. The ribs are very similar overall to those of *Hesperornis*, with a well-developed head and tubercle separated at the proximal end and forming a right angle. Several isolated uncinate processes are preserved with KUVP 2287 and in the slab containing the torso with KUVP 24090. They are rectangular and elongate, and overall similar to those described for *Hesperornis* [7]. Unfortunately, the ribs are too poorly preserved to accurately reconstruct any articulations between specific ribs and uncinate processes.

#### 3.2.5. Pelvic Girdle

**Figure 53:**
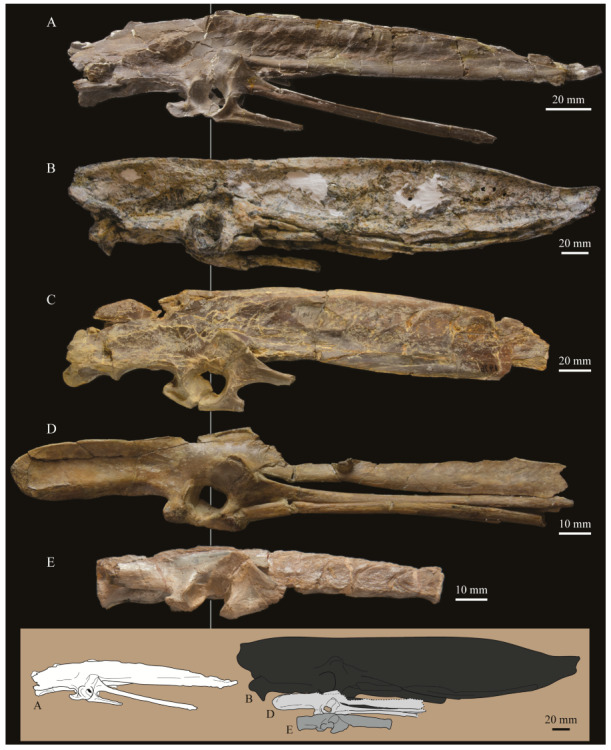
Comparison of the pelves of *Parahesperornis* KUVP 2287 (**A**), *Hesperornis* YPM 1476 (**B**) and SDSM 5312 (**C**), *Fumicollis* UNSM 20030 (**D**), and *Baptornis* AMNH 5101 (**E**) in left lateral view. Elements are scaled to be of a similar acetabular diameter and aligned at the acetabulum. Inset shows silhouettes of the same elements to scale.

The pelvis of *Parahesperornis* is preserved with both KUVP 2287, which preserves most of it except the right ischium (Figure 41), and KUVP 24090. In the latter specimen, the left ischium and pubis are preserved in a slab (together with the left hindlimb and pygostyle) (Figure 43 and Figure 44); and the ilia, and partial right ischium and pubis are preserved in the other slab (together with the ribs and majority of the vertebral column) (Figure 35, Figure 36, Figure 37 and Figure 38). Such excellent preservation of the pelves of both specimens is remarkable; across all known hesperornithiforms, there are fewer than five similarly well-preserved pelves. The description below relies on the following specimens for comparisons: *Baptornis* (AMNH 5101); *Brodavis varneri* (SDSM 68430); *Enaliornis* (BGS 87936, cast as BMNH A5310); *Fumicollis* (UNSM 20030); *Hesperornis* (BMNH A-720, SDSM 5312, SDSM 5860, UNSM 4-19-5-36, YPM 1476); and *Pasquiaornis* (RSM P2626.27, RSMP P2997.62) [65] (Figure 53). While *Hesperornis* specimens FHSM VP-2069 and YPM 1206 both have a well-preserved pelvis, they were on display and not available for close examination at the time of this study. However, both appear to be consistent with other pelves of *Hesperornis* in overall morphology.

**Ilium**. The ilium of *Parahesperornis* is flattened and highly elongate, with the widest point at the acetabulum. The ilium narrows only slightly toward the cranial end, where the cranial-most portions are broken off in KUVP 2287 and covered in matrix in KUVP 24090. It is likely that the proximal end resembled that of *Hesperornis*, which is smoothly rounded. In dorsal view, the preacetabular ilia are neither sutured to one another nor fused along their length; instead, they are joined very closely at the region of the acetabulum, spreading out at the cranial end to accommodate an expanded synsacrum. This is seen to a similar degree in *Hesperornis* and to a lesser degree in *Fumicollis*. The elongation of the preacetabular illium served to expand the area of attachment for the *Mm. iliotrochantericus caudalis* and *cranialis*, which pronates the femur, stabilizing it against supination caused by the retractor muscles of the femur [86]. The division between the cranial and caudal muscles of the *Mm. iliotrochanterici* complex is visible as a broad ridge running diagonally from the cranioventral corner of the preacetabular illium to the dorsal margin above the acetabulum. This is also seen in *Hesperornis* [86], however the ridge is much narrower.

The walls of the preacetabular ilium are nearly straight dorsoventrally in both *Parahesperornis* and *Hesperornis*, however in *Fumicollis* the dorsal margin curves outward, overhanging the wall of the preacetabular ilium. The postacetabular ilium narrows gradually to a very thin flange at the caudal-most end. In lateral view, the ventral margin is fairly flat while the dorsal margin is arched. In *Hesperornis,* the ventral margin arches slightly as well, giving the postacetabular wing of the ilium a more oval outline in lateral view than that of *Parahesperornis.*

The side of the postacetabular ilium has a wide, shallow groove running lengthwise along most of its length, shallowing caudally. Within and dorsal to this groove, the bone forms a wavy pattern of ridges, very similar to those observed in *Hesperornis* and *Fumicollis*. This area is occupied by the attachment of the *m. ischiofemoralis* cranially and the *m. iliiofemoralis* caudally [86]. These muscles are responsible for the rotation and retraction of the femur, respectively [86]. Ventral to this longitudinal groove, the postacetabular ilium extends laterally, with a strongly rounded ridge that originates behind the antitrochanter and extends caudally to merge into the lateral face of the ilium about one-third of the way to the caudal end, such that at the caudal end the ilium is completely flat. This ridge is also prominent in *Hesperornis* but is not present in *Fumicollis*. The caudal-most flattened face is where the *m. flexor cruris medialis* originates. This attachment area is larger in hesperornithids than in modern foot-propelled divers and controls the rotation and placement of the foot during the power stroke [86].

The caudal-most tip of the ilium of *Parahesperornis* curves dorsally. In dorsal view the shape of the postacetabulum echoes that of the preacetabulum, with the ilia joined very tightly along most of their length before separating and flaring laterally at the caudal end, a feature shared with *Hesperornis.* The ratio of the pre- to postacetabular ilium is 0.45 for the right ilium of KUVP 2287 and estimated at 0.31 for the right ilium of KUVP 24090. For comparison, the same ratio for *Hesperornis* specimens is 0.39 (YPM 1476), 0.36 (BMNH A-720), 0.35 (SDSM 5312), and 0.38 (YPM 1206) [7], while in *Fumicollis* this ratio is estimated to be around 0.32 (UNSM 20030). The ratio of the height of the acetabulum to the height of the ilium at the acetabulum is 0.52 for KUVP 2287 and 0.59 for KUVP 24090, while it is 0.57 (YPM 1476), 0.48 (BMNH A-720), and 0.66 (SDSM 5312) in *Hesperornis* specimens. The similarity of these ratios indicates the overall similarities of the pelves of hesperornithids. Other taxa do not have complete enough pelves to compare this ratio.

**Acetabulum**. The acetabulum of *Parahesperornis* is best preserved in KUVP 2287, however that of the right side is also visible in KUVP 24090 (Figure 43 and Figure 44). The acetabulum is formed from the fusion of the ilium (forming the cranial margin and half of the dorsal and ventral margins of the acetabulum), ischium (forming the dorsocaudal margin of the acetabulum), and pubis (forming the caudoventral margin of the acetabulum). The walls of the acetabulum are very steep, as in other hesperornithiforms.

Previous work has referred to the acetabulum of hesperornithiforms as partially closed, as compared to those of modern birds that are completely open [19,21,40]. While the diameter of the acetabulum of hesperornithids is reduced compared to modern birds, as noted by Marsh [7], it does not exhibit the primitive archosaurian state of being imperforate. It should be noted that in all hesperornithiform specimens examined for this study which, to the best of the authors knowledge, includes all of the well and moderately pelves known to date, none preserve a complete acetabulum. In all specimens the acetabulum is open with broken margins. In hesperornithiforms the reduction in size of the acetabulum is associated with the lateral splaying of the femur and, paired with the steep walls of the acetabulum, results in a reduction in the range of motion of the femoral head within the acetabulum and an overall strengthening of this region of the pelvis as compared to modern foot-propelled diving birds [86].

The acetabulum of *Parahesperornis* is very nearly circular, as is the case in most hesperornithiforms. In *Fumicollis*, however, the acetabulum is oval, being wider craniocaudally. The acetabulum faces primarily laterally, but is oriented slightly cranially, as in other hesperornithiforms. The antitrochanter of *Parahesperornis* is formed from the cranial-most end of the ischium and extends dorsally as a flat face from the acetabulum to end in a squared margin that extends laterally from the wall of the ilium.

The antitrochanter is rotated approximately 25-degrees caudally (as measured from the top of the acetabulum) on the acetabulum, as in other hesperornithiforms. The antitrochanters of *Hesperornis, Fumicollis*, and *Baptornis* extend further laterally than in *Parahesperornis*. The antitrochanter of *Pasquiaornis* is not as robust as in other hesperornithiforms, with less lateral extension than in *Parahesperornis*, but is in a similar position on the acetabulum. The acetabulum is the only portion of the pelvis known for *Enaliornis*. The antitrochanter in *Enaliornis* is minimally developed, with very little lateral expansion. The orientation of the acetabulum and antitrochanter on the pelvis indicates the femur of *Parahesperornis* was directed almost entirely laterally, as in most other hesperornithiforms, with the possible exception of *Enaliornis*.

The margins of the acetabulum form a very thin lip on the dorsocranial and ventrocaudal margins. A robust, hooked pectineal process is present on the ventrocranial margin of the acetabulum. The pectineal process of *Hesperornis* is equally robust but lacks the dramatic curve seen in *Parahesperornis*. In *Fumicollis* the pectineal process is large and bulbous, with much less cranial expansion, while in *Baptornis* it is reduced to a small bump on the margin of the acetabulum.

**Ischium and Pubis**. A large portion of the left ischium of KUVP 2287 is preserved in articulation, while other sections of the ischia of both KUVP 2287 and KUVP 24090 are preserved separately. The ischium is mediolaterally compressed, as in other hesperornithiforms, and extends ventrocaudally from the antitrochanter. The ischium is nearly straight along its length, bowing slightly laterally and maintaining a constant width to the broken end. The dorsal surface of the ischium is marked by a shallow groove. Only the cranial-most portion of the pubis is preserved in articulation on both KUVP 2287 and KUVP 24090. The pubis of *Parahesperornis* is straight and slightly thinner dorsoventrally than the ischium, with a groove along the lateral face. From the disarticulated section of pubis preserved with KUVP 2287 it appears that the pubis maintained a constant width along its length.

The ischium and pubis run parallel to each other and extend caudally from the acetabulum angled sharply ventrally. The ilium extends caudally at a much slighter angle, creating a triangular space between the ilium and ischium. The pubis passes beneath the acetabulum and emerges on the cranial side as the pectineal process, as in *Hesperornis* and *Fumicollis*.

While a number of specimens of hesperornithiforms preserve the ischium and pubis in very close contact up against the ilium, it appears that this is a taphonomic consequence of lateral compression. From the remnants preserved near the acetabula (BMNH A-720, YPM 1476), it appears that the orientation of these bones was very much the same in *Hesperornis*, as well as in *Brodavis varneri*. In *Baptornis,* AMNH 5101 the ischium is broken off directly behind the antitrochanter, however it does appear to have been oriented similarly.

#### 3.2.6. Hindlimb

**Figure 54:**
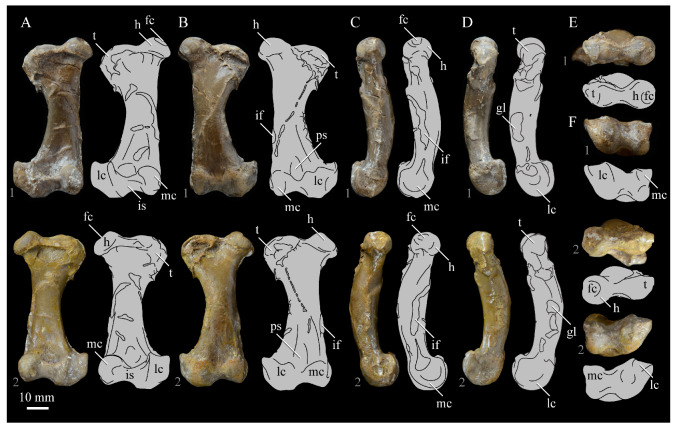
*Parahesperornis alexi* KUVP 2287, photographs and line drawings of left (1) and right (2) femora in caudal (**A**), cranial (**B**), medial (**C**), lateral (**D**), proximal (**E**), and distal (**F**) views. Abbreviations: fc—capital fossa, gl—tuberosity for the m. gastrocnemius lateralis, h—head, if—scar for the m. iliofemoralis internus, is—intercondylar sulcus, lc—lateral condyle, mc—medial condyle, ps—patellar sulcus, t—trochanter.

**Figure 55:**
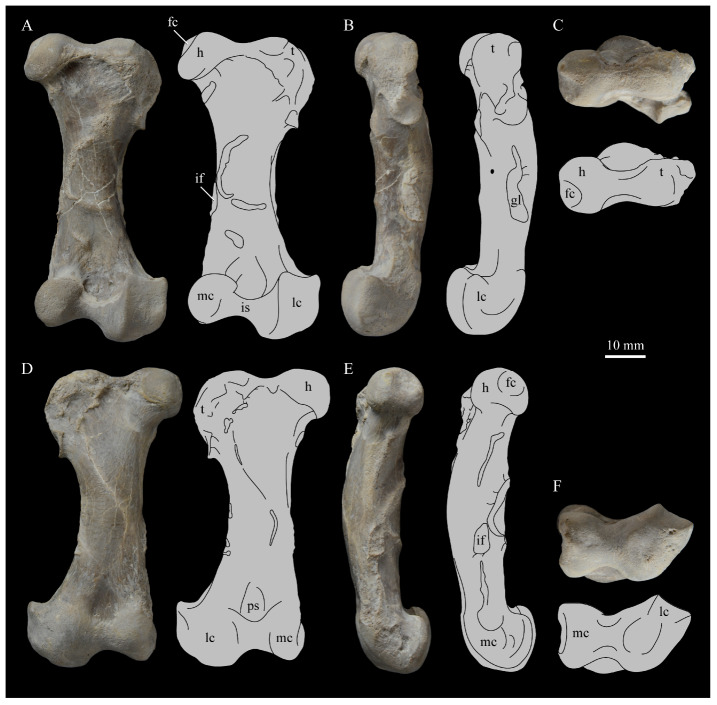
*Parahesperornis alexi* KUVP 24090, photographs and line drawings of isolated right femur in caudal (**A**), lateral (**B**), proximal (**C**), cranial (**D**), medial (**E**), and distal (**F**) views. Abbreviations: fc—capital fossa, gl—tuberosity for the m. gastrocnemius lateralis, h—head, if—scar for the m. iliofemoralis internus, is—intercondylar sulcus, lc—lateral condyle, mc—medial condyle, ps—patellar sulcus, t—trochanter.

**Figure 56:**
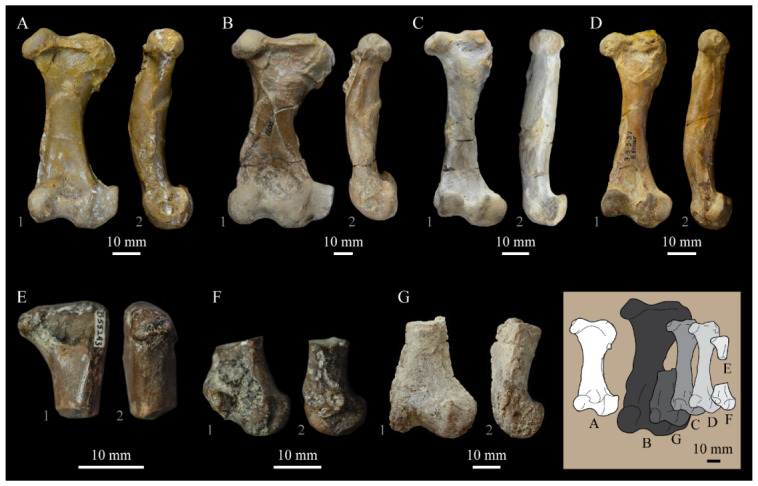
Comparison of the femora of *Parahesperornis* KUVP 2287 (**A**), *Hesperornis* YPM 1200 (**B**), *Baptornis* KUVP 2290 (**C**), *Fumicollis* UNSM 20030 (**D**), *Enaliornis* SMC B55243 (**E**) and SMC 55306 (**F**), and *Brodavis varneri* SDSM 64830 (G) in caudal (1) and medial (2) views. Elements are scaled to be of a similar length or proximal width.

**Femur.** The left and right femora of *Parahesperornis* are preserved with both KUVP 2287 (Figure 54) and KUVP 24090 (Figure 43 and Figure 55). A large number of hesperornithiform femora are known; it is one of the best represented elements for the group. The following specimens were used for comparison to *Parahesperornis*: *Baptornis* (KUVP 2290, FMNH 395); *Brodavis varneri* (SDSM 68430); *Enaliornis* (BMNH A163, BMNH A479, BMNH A5801, SMC B55289, SMC B55300, SMC B55287); *Fumicollis* (UNSM 20030); *Hesperornis* (YPM 1200, YPM 1207, YPM 1476, YPM 1477); and *Pasquiaornis* (RSM P2997.4) (Figure 56).

The femur of *Parahesperornis* displays a number of typical hesperornithiform traits, such as an expanded trochanter and fibular condyle. The proximal and distal ends are evenly expanded mediolaterally, with a narrowed shaft. Slightly proximal to the midpoint of the shaft is waisted, intermediate to the dramatic waist seen in *Hesperornis* and the straighter shaft of other hesperornithiforms.

In medial and lateral views, the femur is bowed, an appearance exaggerated by the high arch of the cranial surface. This condition is better developed in KUVP 2287 than in KUVP 24090. When compared to other hesperornithiforms, KUVP 24090 is most similar in this regard to *Hesperornis*, however the cranial arch is more dramatic in KUVP 2287. Other hesperornithiform femora vary widely in this feature, with *Pasquiaornis* and *Enaliornis* having strongly bowed shafts, *Fumicollis* only bowed at the caudal end, and a nearly straight shaft on *Baptornis*.

In proximal view, the articular surface of the femur of *Parahesperornis* is shaped like a figure eight, with the nearly circular head and the ovate trochanter separated by a narrow, pinched region. The depression for the capitol ligament on the head of the femur is directed proximally, such that it is visible in proximal view. The trochanter narrows to a point on the craniolateral margin of the proximal surface, with a flattened face on the lateral half. In proximal view the craniocaudal width of the head is greater than that of the trochanter.

In cranial or caudal view, the head is directed proximally and extends past the top of the trochanter, more so in KUVP 2287 than KUVP 24090. This condition differs from that of other hesperornithiforms where the head is either even with the trochanter in proximal extent (*Hesperornis, Baptornis*) or does not extend as far proximally (*Fumicollis*, *Pasquiaornis,* possibly *Enaliornis*). The head and trochanter are separated along the proximal margin by a deep indentation, as in other hesperornithiforms. Distal to this indentation on the caudal face of the proximal end, a pronounced ridge is present running mediolaterally across the cranial face, just below the articular surface, extending from the distal margin of the head to the trochanter. In KUVP 2287 this ridge is straight, while in KUVP 24090 it arches proximally, with the peak just below the indentation between the head and trochanter. This ridge is also seen in *Hesperornis*, where it is straight.

In medial view, the head of the femur forms a rounded ball with a slightly flattened distal margin set atop a much narrower neck, as in *Hesperornis* and *Fumicollis. Baptornis* also has a distinct neck below the femoral head, but the distal margin of the head is smoothly rounded. In both *Pasquiaornis* and *Enaliornis*, the neck appears to be less distinct, however the known femora of *Enaliornis* are also highly eroded, which could contribute to the appearance of lacking a neck.

In caudal view, the trochanter forms a broad ridge that wraps around the lateral margin of the femur and ends abruptly about one-fourth the way down the shaft. A small projection is present where the trochanter meets the shaft. Along both the cranial and caudal faces of the femur the margins of the trochanter are clearly delineated. This is also seen in *Hesperornis*. However, in other hesperornithiforms, the trochanter merges more smoothly into the shaft and is less laterally projected. The caudal face of the proximal femur of *Parahesperornis* forms a deep depression medial to the margin of the trochanter, whereas this area is flattened in *Hesperornis*. In lateral view the trochanter of *Parahesperornis* is highly rugose and irregular, as in *Hesperornis*. Due to the bowing of the shaft, the trochanter is angled slightly caudally at the proximal end.

The femoral shaft of *Parahesperornis* is marked by several rugose muscle scars, as in *Hesperornis*, to a somewhat lesser degree in *Fumicollis*, and to a much lesser degree in other hesperornithiforms. One of the most prominent muscle scars is a large tuberosity for the m. gastrocnemius lateralis, which extends the foot during the propulsive stroke [86], located toward the distal end of the lateral side of the shaft. Also prominent is a narrow ridge for the m. puboischiofemorales medialis and the area of attachment of the aponeuroses of the m. popliteus which runs diagonally across the proximal end of the caudal shaft to the distal end of the medial shaft. The m. puboischiofemorales medialis retracts the femur while the m. popliteus stabilizes the knee and inwardly rotates the tarsometatarsus [86]. Galton and Martin [21] describe the attachment for the m. femorotibialis externus as being level with that for the m. gastrocnemius lateralis in *Parahesperornis;* however, the scar for the m. femorotibialis externus is actually distal to that of the m. gastrocnemius lateralis, as has been described for *Hesperornis* [86]. Additionally, the scar for the m. iliofemoralis internus is slightly proximal to that of the m. iliotrochantericus cranialis in *Parahesperornis*, while in *Hesperornis* they are nearly even [86], also contra Galton and Martin [21].

The shaft of the femur narrows into a slight waist mediolaterally just above the midline of the shaft, with the lateral side narrowing more than the medial, which is nearly straight. In medial and lateral views, the shaft is thickest the point of maximum inflection of the cranial surface, at and proximal to midshaft, as in *Hesperornis.* In *Pasquiaornis*, *Enaliornis*, and *Baptornis,* the femoral shaft is only slightly thicker in the midshaft region. The distal lateral shaft of the femur of *Parahesperornis* narrows proximal to the lateral condyle, but to a lesser degree than in *Hesperornis* and *Fumicollis,* where the distal shaft of the femur narrows into a neck just above the lateral condyle.

In cranial and caudal views, the femur of *Parahesperornis* widens from the distal shaft primarily on the lateral side through the dramatic expansion of the lateral condyle common in hesperornithids. The lateral condyle is made up of the caudally directed fibular trochlea and a depression for the articulation of the fibular head, with the tibiofibular crest on the medial margin and a second crest on the lateral margin. The fibular trochlea is oriented to face caudolaterally, as is the case in other hesperornithiforms.

Overall, the lateral condyle is much larger than the medial, as in most hesperornithiforms, but to a greater degree in *Parahesperornis* and *Hesperornis*. In most hesperornithiforms the lateral condyle extends much further distally than the medial condyle, more so than in *Parahesperornis*. However, in *Hesperornis,* the medial and lateral condyles are of similar distal extent, making *Parahesperornis* intermediate to *Hesperornis* and other hesperornithiforms in this regard.

In lateral view, the lateral condyle is semicircular, with a depressed center and a sharp angle at the proximal edge of the caudal margin. The caudal surface of the lateral condyle is a smooth, rounded bulge, as in other hesperornithiforms. The distal surface of the lateral condyle is flattened and bulges slightly, as in *Hesperornis* and *Fumicollis;* in other hesperornithiforms the distal surface of the lateral condyle is smoothly rounded. In distal view the lateral orientation of the condyle is most obvious, being intermediate between *Hesperornis* and *Fumicollis*, which are angled more laterally, and *Baptornis* and *Brodavis*, which are slightly less angled.

The medial and lateral condyles of *Parahesperornis* are separated by a deep intercondylar sulcus, as in other hesperornithiforms. In cranial view the distal surface of the shaft above the intercondylar sulcus forms a depressed patellar sulcus that is bounded distally by a ridge, giving it a pocketed appearance as in *Hesperornis* and to a lesser degree in *Fumicollis;* this distinct morphology is absent in other hesperornithiforms. In caudal view, the distal shaft between the condyles forms a deep popliteal fossa that extends downward into a deep excavation behind the ridge of bone separating the medial and lateral condyles.

The medial condyle is ovate and somewhat kidney-shaped in caudal view and set at an angle to the long axis of the shaft, as in *Hesperornis* and *Fumicollis.* In medial view the medial condyle resembles the lateral but is distinctly smaller. The caudal margin of the medial condyle forms a lip that projects medially, and an elongate tuberosity for the medial collateral ligament is present above the face of the condyle. This condyle is more elongate in medial view in *Hesperornis,* with a rounder tuberosity. The distal shaft narrows into a slight neck just proximal to the medial condyle, as in *Hesperornis*. In distal view the long axis of the medial condyle is oriented perpendicular to the mediolateral axis of the distal end, as in other hesperornithiforms. The medially expanded lip on the cranial margin gives the medial margin of the condyle a c-shaped contour in distal view; this condition is similar to that of Hesperornis and more pronounced than the condition in other hesperornithiforms.

**Figure 57:**
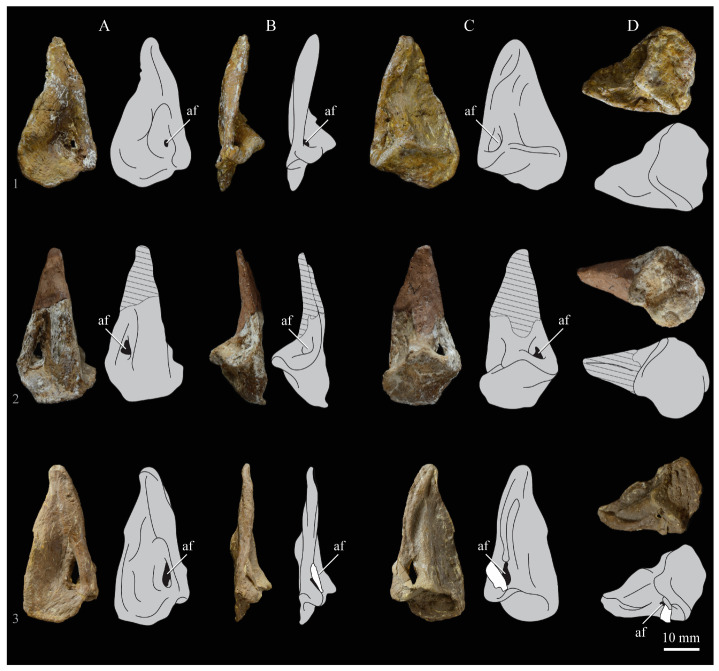
*Parahesperornis alexi* KUVP 2287 (1, 2) and KUVP 24090 (3), photographs (left) and line drawings (right) of patellae in cranial (**A**), lateral (**B**), caudal (**C**), and distal (**D**) views. Hatch marks indicate putty; white piece on 3 is a small flake of indeterminate bone. Abbreviations: af—ambiens foramen.

**Figure 58:**
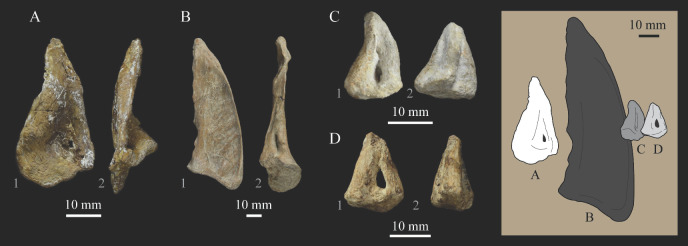
Comparison of the patellae of *Parahesperornis* KUVP 2287 (**A**), *Hesperornis* YPM 1200 (**B**), *Baptornis* KUVP 2290 (**C**), and *Fumicollis* UNSM 20030 (**D**) in cranial (1) and lateral (2) views. Elements are scaled to be of a similar distal width and aligned at the proximal ends. Inset shows silhouettes of the same elements to scale.

Patella. The patella of *Parahesperornis* is preserved with both KUVP 2287 and KUVP 24090 (Figure 57), the left of which is encased in chalk (Figure 43 and Figure 44). Comparisons were made with the following hesperornithiform specimens: *Baptornis* (KUVP 2290); *Fumicollis* (UNSM 20030); and *Hesperornis* (YPM 1200, YPM 1207) (Figure 58). Patellae are unknown for other hesperornithiform taxa.

In general, the patella of *Parahesperornis* is very similar to that of *Hesperornis.* Hesperornithid patellae differ dramatically from those of other hesperornithiforms in being extremely elongate, with a shape like an isosceles pyramid with elongate sides and a very short base, as opposed to the nearly equilateral pyramid seen in *Baptornis* and *Fumicollis,* where the sides and base are similar lengths. The patella of *Parahesperornis* is proportionally much less elongated than that of *Hesperornis*. The distal width is approximately 50% of the length in Parahesperornis, while in *Hesperornis* it is approximately 35%. In cranial view the medial margin is curved while the lateral margin is straight and the distal margin angles down to the medial margin, as in *Hesperornis*. The laterodistal corner of the cranial face is penetrated by a small foramen for the ambiens tendon, which emerges as a larger foramen on the lateral side of the patella. This foramen is a thinner slit in *Baptornis* and a broader teardrop shape in *Fumicollis*.

In lateral view the patella of *Parahesperornis* is extremely thin, like that of *Hesperornis*. The patellae of both *Baptornis* and *Fumicollis* are much wider in this view, comparable to the width in cranial view. The proximal portion of the patella is very thin craniocaudally, leading to a high degree of deformation in most specimens of both *Parahesperornis* and *Hesperornis.* The distal face of the patella is set at a high angle, such that it is visible in caudal and medial views, as in *Hesperornis*. The completely preserved patella of KUVP 2287 is also curved caudally along the proximal half, as is the patella of *Baptornis*, while those of KUVP 24090 and *Hesperornis* are straight. This difference does not seem to be the result of taphonomy.

**Figure 59:**
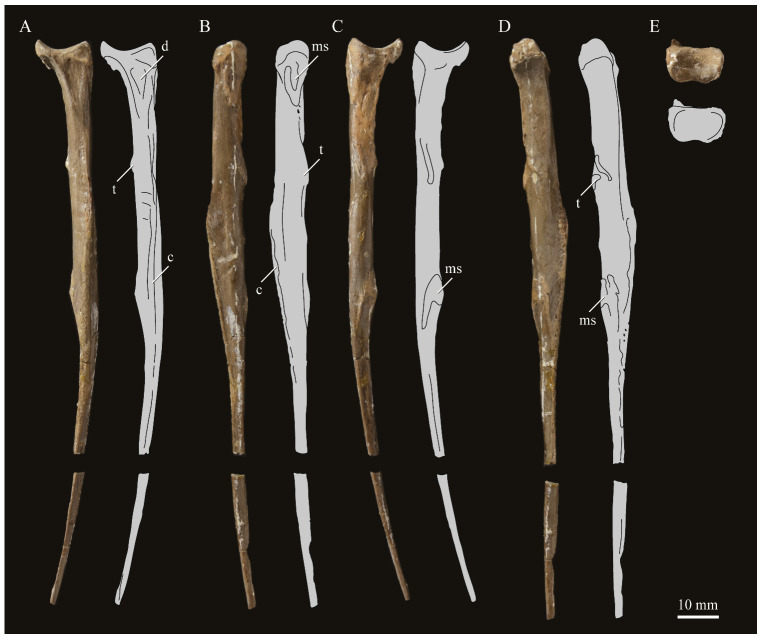
*Parahesperornis alexi* KUVP 2287, photographs (left) and line drawings (right) of right fibula in medial (**A**), cranial (**B**), lateral (**C**), caudal (**D**) and proximal (**E**) views. Abbreviations: c—crest, ms—muscle scar, t—tubercle for *M. iliofibularis.*

**Figure 60:**
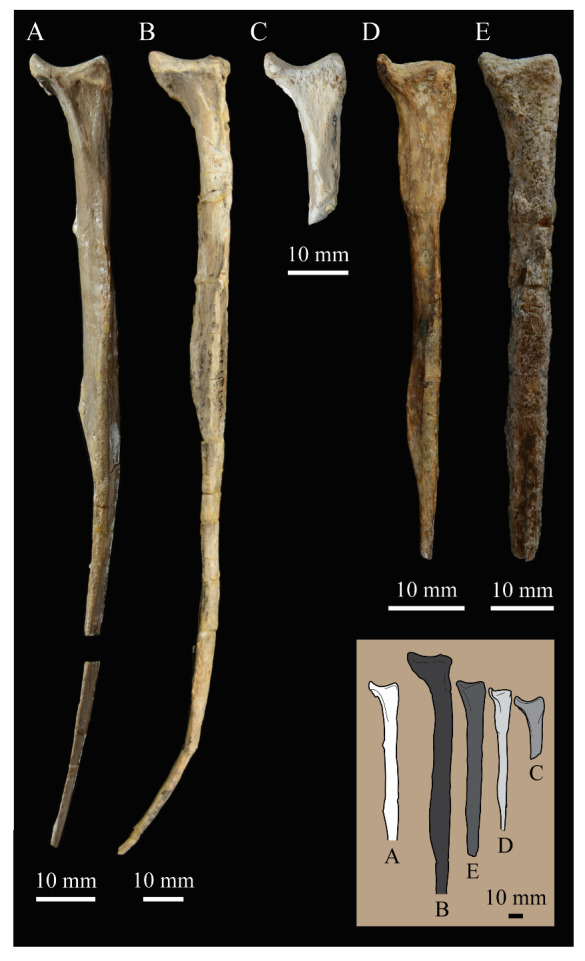
Comparison of the fibulae of *Parahesperornis* KUVP 2287 (**A**), *Hesperornis* YPM PU17193 (**B**), *Baptornis* KUVP 2290 (**C**), *Fumicollis* UNSM 20030 (**D**), and *Brodavis varneri* SDSM 64830 (**E**) in medial view. Elements are scaled to be of a similar proximal width and aligned at the proximal ends. Inset shows silhouettes of the same elements to scale, with the length of A and B truncated.

**Fibula.** The left fibula of *Parahesperornis* is preserved with KUVP 2287 (Figure 59) and both are preserved with KUVP 24090 (Figure 43 and Figure 44). The total length of KUVP 2287 is preserved, however the distal extent is broken as a separate piece that can be cleanly fitted back to the remainder of the shaft. The left fibula of KUVP 24090 is completely preserved and exposed in caudal view, in articulation with the tibiotarsus, encased in chalk. To the best of the authors’ knowledge, these are the best preserved hesperornithiform fibulae, as all other specimens examined or published preserve a lesser portion of the shaft or are broken and reconstructed. Comparisons were made with the following specimens: *Baptornis* (KUVP 2290); *Brodavis varneri* (SDSM 68430); *Fumicollis* (UNSM 20030); *Hesperornis* (AMNH 2181, YPM 1499, YPM 1200, YPM PU 17193) (Figure 60).

The fibula of *Parahesperornis* is crutch-shaped, with the proximal surface expanded on the cranial side. The shaft is roughly oval in cross section and tapers along its length to a thin sliver at the distal-most end, very similar to the fibula of other hesperornithiforms. The proximal surface forms a deep depression, which resembles the seat of a saddle in medial and lateral view. This is intermediate between *Hesperornis* and other hesperornithiforms, which is less deeply excavated, and *Baptornis*, which is more deeply excavated. The medial face of the fibula of *Parahesperornis* has a triangular depression, with a small foramen centered on the proximal margin. A similar depression is only faintly developed in *Hesperornis* and *Fumicollis*, while in *Baptornis* no depression is evident. In lateral view the face of the proximal end bulges outward on the cranial side, as in other hesperornithiforms. A large muscle scar is visible on the cranioproximal end of the fibula, as in *Hesperornis*, but which is not visible in other hesperornithiforms.

The shaft of the fibula of *Parahesperornis* is irregularly shaped. The tubercle for the *m. iliofibularis* is present almost one-fourth of the way down the shaft on the caudal margin (best seen in medial view). Distal to this tubercle, just below the midpoint of the shaft, the bone narrows dramatically, with its sides forming a sharp angle in medial and lateral view, as in *Hesperornis* and *Fumicollis*. A crest is present along the medial face of the shaft where the fibula abuts the tibiotarsal shaft. This crest is also present in *Hesperornis* but does not extend as far distally. This crest may serve as the attachment of the ligaments that connect the fibula to the tibiotarsus.

**Figure 61:**
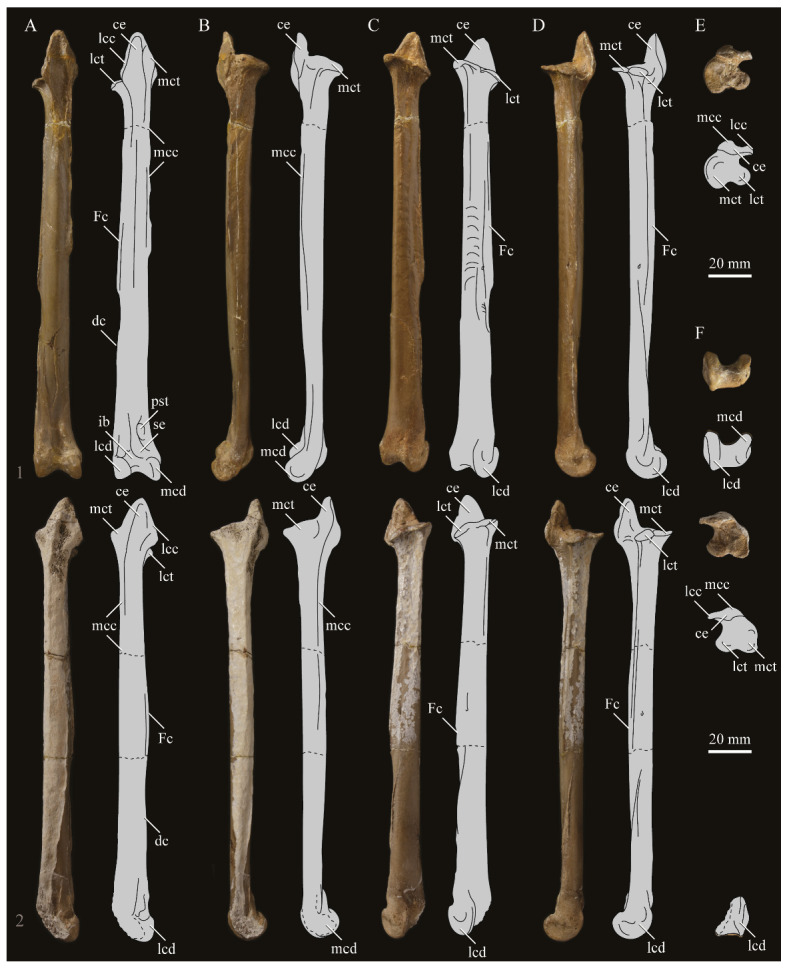
*Parahesperornis alexi* KUVP 2287, photographs (left) and line drawings (right) of left (1) and right (2) tibiotarsi in cranial (**A**), medial (**B**), caudal (**C**), lateral (**D**), proximal (**E**), and distal (**F**) views. Abbreviations: ce—cnemial expansion, dc—distal crest, Fc—fibular crest, ib—intercondylar bridge, lcc—lateral cnemial crest, lcd—lateral condyle, lct—lateral cotyla, mcc—medial cnemial crest, mcd—medial condyle, mct—medial cotyla, pst—tubercle, se—extensor sulcus.

**Figure 62:**
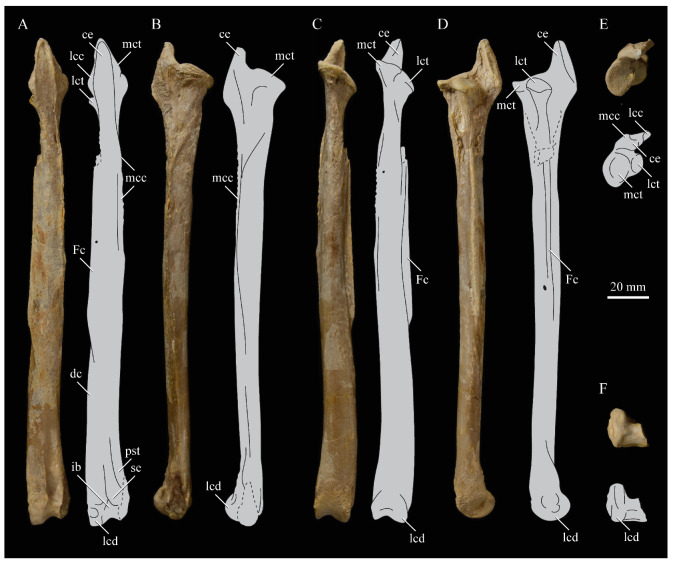
*Parahesperornis alexi* KUVP 24090, photographs (left) and line drawings (right) of right tibiotarsus in cranial (**A**), medial (**B**), caudal (**C**), lateral (**D**), proximal (E), and distal (**F**) views. Abbreviations as in Figure 61.

**Figure 63:**
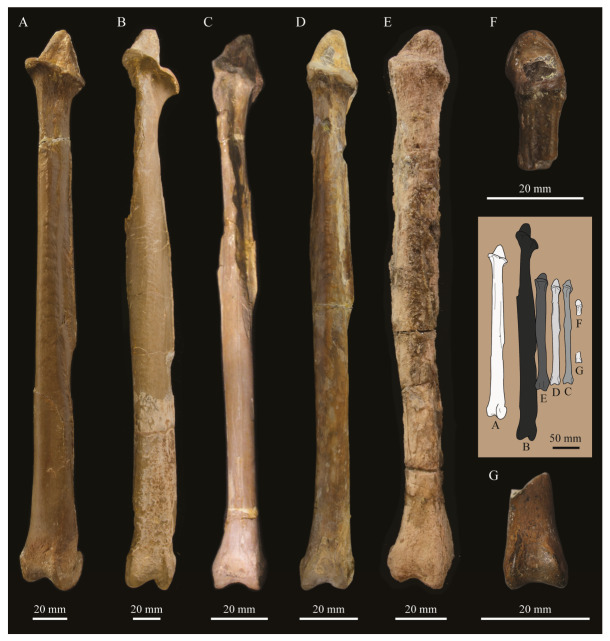
Comparison of the tibiotarsi of *Parahesperornis* KUVP 2287 (**A**), *Hesperornis* AMNH 2181 (**B**), *Baptornis* FMNH 395 (**C**), *Fumicollis* UNSM 20030 (**D**), *Brodavis varneri* SDSM 64830 (**E**), and *Enaliornis* SMC B55315 (**F**) and SMC B55314 (**G**) in caudal view. Elements are scaled to be of a similar length in the case of complete elements and articular width in the case of partial elements and aligned at the proximal or distal ends. Inset shows silhouettes of the same elements to scale.

**Tibiotarsus.** Both tibiotarsi of *Parahesperornis* are preserved with KUVP 2287 (Figure 61) and KUVP 24090. In KUVP 24090the right is incomplete (Figure 62) while the left is complete and encased in chalk in caudal view (Figure 43 and Figure 44). For comparison, the following specimens were used: *Baptornis* (AMNH 5101, KUVP 2290, FMNH 395); *Brodavis varneri* (SDSM 68430); *Enaliornis* (SMC B55315, SMC B55314); *Fumicollis* (UNSM 20030); *Hesperornis* (AMNH 2181, YPM 1200, YPM 1476); and *Pasquiaornis* (RSM P2957.21, RSM P2957.22) (Figure 63). The tibiotarsus of *Parahesperornis* has several typical hesperornithiform features, in particular an expanded proximal process for the cnemial crests, a gap in the fibular crest, and a medially deflected medial condyle.

The proximal portion of the tibiotarsus of *Parahesperornis* is very similar to that of *Hesperornis*. In proximal view, the medial cotyla is large and kidney shaped, separated from the cnemial expansion on the cranial margin by a groove. In medial view, the articular surface of the medial cotyla slopes caudally, with the margin of the cotyla thickened and rugose. The lateral cotyla is circular, much smaller than the medial, and displaced caudally, such that it has a similar caudal extent as that of the much larger medial cotyla. While the cotylae are similarly unevenly sized in *Hesperornis,* they are not levelled caudally, and the lateral cotyla is elongate and oval shaped. In *Fumicollis* the size discrepancy between the cotylae is not as exaggerated as in hesperornithids, a reflection of the smaller discrepancy between femoral condyles, as is also seen in other hesperornithiforms. Cranial to the lateral cotyla is a deep indentation between this cotyla and the cnemial expansion, for articulation with the proximal end of the fibula.

In cranial view, the cnemial expansion is triangular, with the medial corner higher proximally than the lateral. While the cnemial expansion of KUVP 24090 appears to be narrower than that of KUVP 2287, this is due to the crushing of the proximal end of KUVP 24090. The lateral cnemial crest is sharp and narrow, running along the lateral margin of the cnemial expansion. The lateral cnemial crest extends distally approximately one-fifth the length of the shaft, while the medial cnemial crest extends to about midshaft. On the cnemial expansion, the medial cnemial crest is broader than the lateral. The cnemial expansion of *Hesperornis* is more oval in cranial view, while that of *Fumicollis* and *Brodavis varneri* is also triangular. Across hesperornithiforms, the cnemial crests are equally well-developed, varying only in their shape. While the cnemial crests of *Enaliornis* and *Brodavis varneri* are rounded proximally, that is likely due to the poor preservation of the specimens, which are all heavily eroded. 

The proximal shaft of the tibiotarsus of *Parahesperornis* narrows very slightly below the medial cotyla, which forms a slight lip at its caudal-most extent. In caudal view, the articular surface is angled, with the medial side highest, and expanded mediolaterally past the margins of the shaft, as in *Hesperornis*. In *Fumicollis* the articular surface is also angled, but not as greatly expanded mediolaterally, while in *Baptornis* and *Brodavis* the articular surface is only slightly angled and expanded. A slight depression is present on the proximal face of the caudal shaft just below the overhanging articular surfaces. This depression is deeper in *Hesperornis* and faint in *Fumicollis* and *Baptornis.*

The tibiotarsal shaft of *Parahesperornis* is straight in caudal view and oval in cross-section. In both cranial and caudal views, the shaft of the tibiotarsus is narrowest proximally and very gradually expands distally, while in medial and lateral view the shaft is a constant width until just above the distal condyles, where it then narrows. The shaft is slightly twisted, such that when the distal end is in cranial view the proximal end is in craniomedial view. This is also seen in *Hesperornis* and *Fumicollis.* The shaft is slightly bowed along its length, such that in medial or lateral view the shaft is cranially convex just below midshaft. This bowing is more pronounced in KUVP 24090 than in KUVP 2287. In KUVP 24090 the shaft appears to be sinusoidal in cranial and caudal view, while that of KUVP 2287 is straight. The poor preservation of many of the tibiotarsi assigned to *Hesperornis* make observation of the straightness of the shaft difficult, as some specimens appear to be perfectly straight (e.g., YPM 1200), while others more closely resemble KUVP 24090 with a prominent bow and sinusoidal shape (e.g., YPM 1476). The tibiotarsus of *Fumicollis* is slightly bowed and sinusoidal.

In *Parahesperornis,* the fibular crest extends from the neck of the lateral cotyla distally to just below midshaft, as in *Hesperornis*. In *Fumicollis* and *Baptornis* the fibular crest ends at midshaft. At this point there is a gap in the fibular crest before the proximal end of the less prominent distal fibular crest that originates just above the lateral condyle, as is also the case for *Hesperornis* and *Fumicollis. Baptornis* lacks this distal part of the fibular crest. In cranial view, the fibular crest has minimal lateral projection, being about the same as that of the medial cnemial crest, however in caudal view the fibular crest is much wider, with a faint groove running along the medial margin separating it from the shaft of the tibiotarsus. This groove is more prominent in KUVP 24090 than KUVP 2287, where the fibular crest also appears to be less laterally projected than in KUVP 2287.

The distal end of the tibiotarsus expands slightly on the lateral side but flares widely on the medial side, such that the center of the medial condyle is in line with the medial margin of the shaft. This is like the condition in *Fumicollis* and *Baptornis,* and more exaggerated than in *Hesperornis.* In other hesperornithiforms the distal end is centered on the shaft and evenly expanded mediolaterally. In cranial view, the distal shaft has a deep central extensor groove that ends in a slight pocket behind the intercondylar bridge. The cranial face of the lateral shaft is broad and flat, extending over the extensor groove. A slight depression is present on the shaft just proximal to the lateral condyle, as in other hesperornithiforms. A large tuberosity is present on the medial side of the cranial shaft, extending into the extensor groove. The tuberosity is also seen in *Hesperornis* but not in other hesperornithiforms. No true supratendinal bridge is present in hesperornithiforms, however a ridge is present between the condyles that spans the extensor groove distally.

The medial and lateral condyles of *Parahesperornis* are of a similar size in cranial view, with a deep intercondylar groove between them and the medial condyle extending slightly further distally than the lateral, as in *Hesperornis*. In other hesperornithiforms the medial condyle extends past the lateral to a greater degree. In medial view, the medial condyle is much smaller than the lateral, such that the lateral condyle can be seen on either side of the medial. The caudal margin of the medial condyle forms a lip around the medial face of the condyle, which has a prominent tubercle for the attachment of the medial collateral intertarsal ligament [86] on its face. The entire condyle appears to be angled caudally in medial view, and overall appears very much like the medial condyle of *Hesperornis*. In *Fumicollis* and *Baptornis,* the medial condyle is in line with the shaft. However, it is not as reduced as in hesperornithids and expanded cranially.

In caudal view, both condyles form narrow epicondylar crests that wrap up onto the cranial surface of the shaft. In lateral view, the lateral condyle is somewhat rounded, being nearly circular along the caudal margin but flattened along the cranial margin. The cranial margin of the lateral condyle projects past the shaft. In contrast, the lateral condyle of *Hesperornis* is nearly circular in lateral view and centered on the distal shaft. In *Fumicollis* and *Baptornis,* the lateral condyle is further projected cranially than in *Parahesperornis*. The center of the lateral face of the condyle has a round tuberosity with a deep depression on its craniodistal border.

In distal view the condyles are aligned mediolaterally, with the lateral condyle much wider craniocaudally than the medial. This is also the case in *Hesperornis*, however in *Fumicollis* the condyles are more similarly sized. The craniocaudal axes of the condyles angle toward each other cranially. This feature may be highly subject to taphonomic distortion, as in some specimens of *Hesperornis* the orientation of the craniocaudal axes of the distal condyles varies dramatically between left and right elements of an individual (e.g., YPM 1476).

**Figure 64:**
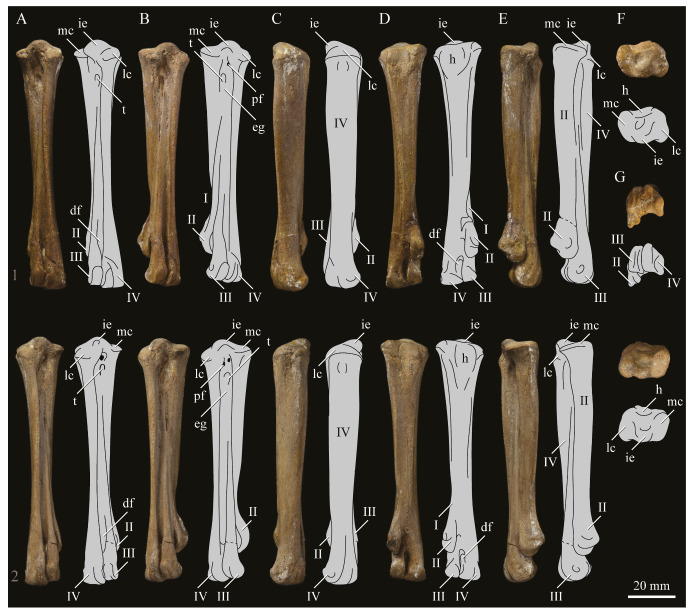
*Parahesperornis alexi* KUVP 2287, photographs (left) and line drawings (right) of left (1) and right (2) tarsometatarsi in dorsolateral (**A**), dorsomedial (**B**), lateral (**C**), plantar (**D**), medial (**E**), proximal (**F**), and distal (**G**) views. Abbreviations: df—distal foramen, eg—extensor groove, h—hypotarsal area, ie—intercotylar eminence, lc—lateral cotyla, mc—medial cotyla, pf—proximal foramina, t—tubercle for the *m. tibialis cranialis*, I—area of articulation of metatarsal I, II—metatarsal II, III—metatarsal III, IV—metatarsal IV.

**Figure 65:**
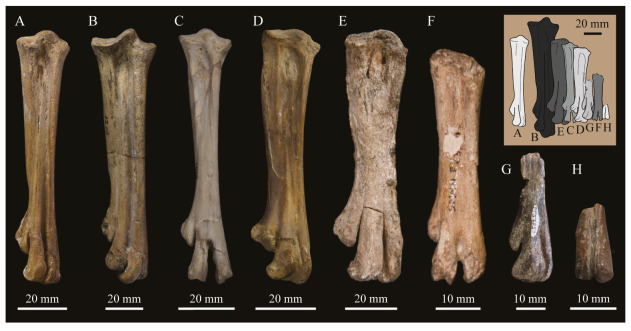
Comparison of the tarsometatarsi of *Parahesperornis* KUVP 2287 (**A**), *Hesperornis* YPM 1200 (**B**), *Baptornis* AMNH 5101 (**C**), *Fumicollis* UNSM 20030 (**D**), *Brodavis varneri* SDSM 64830 (**E**), *Brodavis americanus*, cast of RSM P2315.6 (**F**), *Pasquiaornis* RSM P2077.79 (**G**), and *Enaliornis* SMC B55321 (**H**) in dorsomedial view. Elements are scaled to be of a similar length in the case of complete elements and a similar distal width in the case on incomplete elements and aligned at the distal ends. Inset shows silhouettes of the same elements to scale.

**Tarsometatarsus.** The left and right tarsometatarsi of *Parahesperornis* are preserved with KUVP 2287 (Figure 64). KUVP 24090 does not preserve either tarsometatarsus. An additional tarsometatarsus, FHSM VP-17312, has previously been assigned to the genus *Parahesperornis* [26]. The following specimens were used for comparisons: *Baptornis* (AMNH 5101, FMNH 395, YPM 1465); *Brodavis baileyi* (UNSM 50665); *Brodavis varneri* (SDSM 68430); *Fumicollis* (UNSM 20030); *Hesperornis regalis* (YPM 1200, YPM 1207, YPM 1476); *Hesperornis gracilis* (YPM 1473, YPM 1478); and *Pasquiaornis* (RSM P2997.18, RSM P2997.81) (Figure 65). The tarsometatarsus of *Parahesperornis* exhibits the typical hesperornithiform features of a prominent intercotylar eminence, dorsal surface of the fourth metatarsal forming a sharp ridge, expanded trochlea on the fourth metatarsal, reduced and proximoplantarly displaced second metatarsal trochlea, and shingled or stacked arrangement of the metatarsals.

In proximal view, the tarsometatarsus of *Parahesperornis* is somewhat rectangular in outline, with a longer mediolateral width than craniocaudal depth, but with the medial and lateral margins angled such that the outline is rhombic overall. The proximal articular surfaces of both *Hesperornis* and *Fumicollis* are more rectangular, with a greater discrepancy between the craniocaudal and mediolateral dimensions, while that of *Baptornis* is more like *Parahesperornis*. Both cotylae are oval and craniocaudally expanded, with the medial slightly longer than the lateral, as in most other hesperornithiforms except for *Baptornis*, where the lateral cotyla is longer than the medial. The plantar margins of both cotylae are smoothly rounded and lack the flange that is prominent in *Hesperornis* and less developed in *Fumicollis* and *Baptornis*.

The intercotylar eminence is broad and rounded, as in all other hesperornithiforms except *Hesperornis,* where it is narrow and peaked. The intercotylar eminence is centrally located along the dorsal margin of the articular surface, as in other hesperornithiforms. In dorsal view, the intercotylar eminence projects proximally and is slightly angled laterally with a small depression on the medial margin, as in most other hesperornithiforms except *Baptornis*, where it is not angled. On the plantar slope of the intercotylar eminence, there is a slight depression that is found in all hesperornithiforms. In medial and lateral views, the intercotylar eminence of the isolated *Parahesperornis* tarsometatarsus FHSM VP-17312 is narrower and more pointed than in KUVP 2287.

In plantar view, the proximal articular surface of the tarsometatarsus of *Parahesperornis* is angled, with the medial side highest. This is typical in hesperornithiforms, to a similar degree in *Fumicollis* and *Baptornis* and to a greater degree in *Hesperornis*. In medial view, the proximal end of metatarsal II of *Parahesperornis* has a flattened surface that angles dorsoplantarly, with a rounded plantar margin and a sharper dorsal margin. Below this the shaft of metatarsal II narrows at a slight neck, less pronounced than that seen in *Hesperornis* and more like that of *Fumicollis*.

The plantar margin of the articular surface forms the proximal extent of a rudimentary hypotarsus. Most hesperornithiforms have a triangular, roughened patch of bone that covers the proximal plantar surface of the tarsometatarsus and wraps up onto the dorsal edge of the articular surface. In *Parahesperornis* this surface is delineated by grooves on the proximal articular surface, as well as on the medial and lateral margins of the plantar surface, as in *Hesperornis*. In other hesperornithiforms these edges are not well marked. The hypotarsus is more elongate in *Parahesperornis* than in *Hesperornis*. This area is only faintly developed in *Fumicollis* and *Baptornis.* In *Brodavis varneri* the hypotarsal area is not preserved, however two very deep grooves or slits are preserved that coincide with the margins of metatarsal III.

On the dorsal surface of the shaft, the proximal portions of metatarsals II and IV are developed as ridges, with metatarsal III deeply depressed between them to form the extensor groove, as in other hesperornithiforms. Within the proximal-most part of this depression are two oval foramina, the medial of which is slightly proximal to the lateral. These foramina do not penetrate to the plantar surface of the tarsometatarsus, as in modern birds, and they are found in all hesperornithiforms. In *Parahesperornis*, *Hesperornis*, *Fumicollis,* and *Brodavis varneri,* the proximal foramina are fairly large and either oval or teardrop shaped, while in *Baptornis* they are much smaller and circular.

Distal to the proximal foramina is an elongate depression or small groove with a tuberosity along the distal margin. This is similar to the condition in *Fumicollis*, while in *Hesperornis* the depression is reduced or absent and in *Baptornis* the tuberosity is reduced. This tuberosity is interpreted as the attachment of the *m. tibialis cranialis*. In lateral view the margin of metatarsal IV is evenly rounded with a depression on the proximolateral face, as in *Fumicollis*. In *Hesperornis* the corresponding area is flattened and tilted dorsally.

The shaft of the tarsometatarsus of *Parahesperornis* is straight and narrows gradually along its length in dorsal view. Also in this view, the metatarsals are separated by grooves along their length, with the dorsal surface of metatarsal IV forming a high, rounded ridge, as in *Hesperornis*. Distally, these grooves become much more deeply excavated, while in *Hesperornis* they are deeply excavated along the length of the shaft. The grooves extend the full length of the shaft but are less prominent in *Fumicollis*, while in *Baptornis* and *Brodavis* they only extend to the midshaft.

The shaft of the tarsometatarsus of *Parahesperornis* is twisted, such that when the proximal end is in dorsal view the distal end is in dorsomedial view. While all hesperornithiforms share this trait to some degree, the degree of rotation in *Parahesperornis* is most like that of *Fumicollis*, as *Hesperornis* is more twisted and *Baptornis* and *Brodavis* are less twisted. In plantar view the shaft of *Parahesperornis* forms a continuous flattened surface with a slight central depression bordered on the medial and lateral edges by thin ridges, as in *Hesperornis* and *Fumicollis*, while the depression is very faint in *Baptornis*. In medial view the shaft of metatarsal II is slightly sinusoidal in shape, curving proximally and distally, as in *Fumicollis*. *Hesperornis* has a similar, but less dramatic, shape, while that of *Brodavis* is more dramatically curved. In *Baptornis* the shaft of metatarsal II is bowed in medial view.

Distally, the tarsometatarsus of *Parahesperornis* is like those of *Hesperornis* and *Fumicollis*. Between the necks of trochlea III and IV, the deep groove between the metatarsals terminates in a distal foramen that penetrates through to the plantar surface. In plantar view, the distal foramen is oval and set within a larger tear-drop shaped hollow between the metatarsals, as in *Hesperornis* and perhaps *Fumicollis,* although in UNSM 20030 the area is obscured partially by sediment. While the distal foramen is seen in all hesperornithiforms, the spacing of the metatarsal trochlea distal to the foramen varies. In *Parahesperornis*, like in *Hesperornis* and *Fumicollis*, the trochleae are pressed tightly together, while in other hesperornithiforms the trochleae are slightly further apart, including the isolated tarsometatarsus assigned to *Parahesperornis* (FHSM VP-17312).

The metatarsal trochleae of hesperornithiforms are offset dorsoplantarly to varying degrees, giving the metatarsals a shingled or stacked appearance in medial view, with trochlea II displaced plantarly such that it is almost completely behind metatarsal III. The most extreme form of this morphology is seen in *Hesperornis*. In *Parahesperornis* and other hesperornithiforms trochlea II is less displaced plantarly and is more set on the medial side of metatarsal III. In *Parahesperornis* trochlea IV is larger than II or III, being slightly wider dorsoplantarly (best seen in medial view) and almost twice as wide mediolaterally (best seen in distal view). Trochlea IV also extends slightly further distally than III. This difference is seen to varying degrees in other hesperornithiforms. In *Hesperornis* the size discrepancy is greater with trochlea IV extending much further distally, while in *Pasquiaornis* it is less than in *Parahesperornis*. *Fumicollis* is the most like *Parahesperornis* in terms of the size discrepancy and distal extent of trochlea III and IV.

Trochlea II appears somewhat triangular in dorsal view, with the medial trochlear ridge displaced proximally, as in other hesperornithiforms. In medial view, the plantar margin of the medial face of the trochlea extends into a small flange, like that seen in *Hesperornis*. In *Baptornis* this flange is somewhat better developed and slightly medially directed, while in *Fumicollis* the margin is evenly rounded without a flange. The medial face of trochlea III and the lateral face of trochlea IV are marked with a circular depression for the attachment of the collateral ligaments [86]. In distal view, the size discrepancy in the medial and lateral trochlear ridges of trochlea IV is easily seen, with the medial ridge about 70% the length of the lateral. This is very similar to the condition in *Hesperornis* and much more exaggerated than in other hesperornithiforms. The plantar-most margin of the lateral trochlear ridge flips laterally, to a lesser degree than in *Hesperornis,* giving the trochlea a slight c-shape on the lateral margin. Trochlea III is triangular in shape in distal view with similarly sized medial and lateral trochlear ridges, as in other hesperornithiforms.

**Figure 66:**
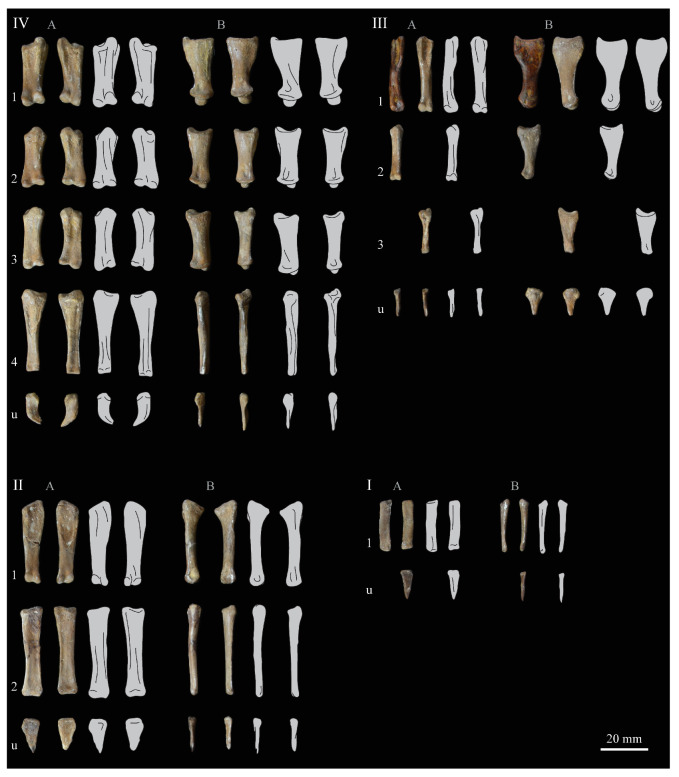
*Parahesperornis alexi* KUVP 2287, photographs and line drawings of left (left within columns) and right (right within columns) of pedal phalanges belonging to digits IV (upper left; 1–4 and u), III (upper right, 1–3 and u), II (lower left, 1–2 and u), and I (lower right, 1 and u) in plantar (**A**) and lateral (**B**) views. Abbreviation: u—ungual.

**Figure 67:**
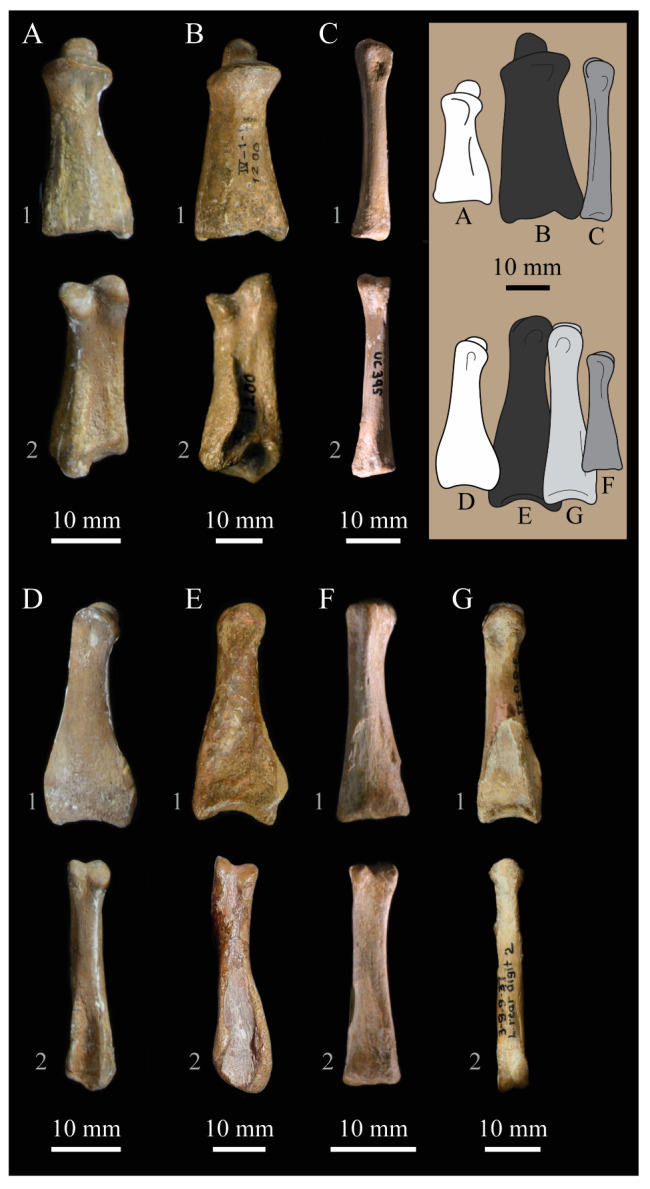
Comparison of the pedal phalanx 1 of digit IV (**A**–**C**) and pedal phalanx 1 of digit III (**D**–**G**) of *Parahesperornis* KUVP 2287 (**A**,**D**), *Hesperornis* YPM 1200 (**B**,**E**), *Baptornis* FMNH 395 (**C**,**F**), and *Fumicollis* UNSM 20030 (**G**) in lateral (1) and plantar (2) views. Elements are scaled to be of a similar length and aligned at the proximal ends. Inset shows silhouettes of the same elements to scale.

**Phalanges.** Nearly all the phalanges of *Parahesperornis* are preserved in KUVP 2287: complete digits III and IV, including the ungual phalanges, from both the left and right feet; the first phalanx and ungual phalanges are preserved from both the left and right side of digit II, while the second phalanx is only preserved from the left side and the third is only preserved from the right, and from digit I the terminal phalanges are preserved from the left and right side while only the right ungual phalanx is preserved (Figure 66). These phalanges are very similar in form to those of *Hesperornis*. For comparison the following specimens were used: *Baptornis* (FMNH 395); *Fumicollis* (UNSM 20030); and *Hesperornis* (FHSM VP- 2069, YPM 1200, YPM 1476) (Figure 67).

The phalanges of digit IV of *Parahesperornis* are overall very similar to those of *Hesperornis* and reflect the unique morphology of the enlarged metatarsal IV trochlea and the enlarged medial ridge of that trochlea in hesperornithids. Unlike *Fumicollis* and *Baptornis*, the phalanges of digit IV of hesperornithids are robust and cylindrical, with a triangular cross-section distally and a circular cross section proximally. The proximal articular surface has an enlarged, oval medial cotyla and a much smaller, circular lateral cotyla. At the plantar end of the medial cotyla the articular surface curves proximally into a high peak. This peak is the proximal-most extent of a dramatic flange that extends plantarly from the medial side of the phalanx. This flange is also present in *Hesperornis*, where it is proportionally larger. The plantar surface of digit IV has a flattened dorsal surface with a depression along its length. The medial and lateral sides of digit IV phalanges are smoothly rounded. Distally, the body of the phalanx narrows, with a slight neck just before the distal trochlea. The medial and lateral trochlea are unevenly sized, with the lateral a small peg projected much further distally than the broader, crescent-shaped medial condyle, as in *Hesperornis*.

This morphology is extremely different from that of *Baptornis*. The phalanges of the fourth toe of *Baptornis* are much more elongate with a circular cross section. Proximally, the articular surface is not divided into separate cotylae for the trochlear ridges of metatarsal IV, and the distal end is only weakly divided into trochleae. The plantar face is slightly flattened, however the groove along its surface is lacking.

The first toe of digit IV is longer than the second and third, which are similarly sized, while the fourth toe is longest and less robust than the preceding phalanges. The ungual phalanx, or claw, is small and triangular, with a hooked appearance in dorsal or plantar view not seen in the other unguals. In *Hesperornis* the phalanges of the fourth toe have a similar morphology, however the fourth phalanx is much thinner in *Parahesperornis*, being almost flattened dorsoplantarly. It is possible this is due to preservational factors. The ungual phalanx for the fourth toe is most likely not preserved in any of the *Hesperornis* specimens accessible for this study, as none display the prominent hook shape seen in *Parahesperornis*.

The size discrepancy between the articular surfaces of metatarsal trochlea IV and the phalanges of digit IV has implications for the movement of the toes. As digit IV is bent, at each joint the proximal bone will swivel around the smaller medial condyle on the longer, crescent-shaped lateral condyle. This, combined with the stacked orientation of the metatarsals within the tarsometatarsus, functions to streamline the foot and increase the range of motion of the longest toe. This has led to the conclusion that hesperornithiforms may have had lobed feet, as in modern grebes [19]. This conclusion is reasonable, as grebes share the size discrepancy in the articular surfaces of the toes and generate lift during the swimming stroke by separating and rotating their toes, thus using each lobe as a separate hydrofoil [87]. However, it should be noted that coots also have lobed toes but do not have unevenly sized condyles, and only hesperornithids show this size discrepancy in the condyles of the fourth toe.

The phalanges of digit III of *Parahesperornis* are very different from those of digit IV, reflecting the much narrower third metatarsal trochlea. In *Parahesperornis* these phalanges are very narrow mediolaterally and triangular in medial or lateral view, with a greatly expanded proximal end and a long neck, as in *Hesperornis*. The phalanges of digit III of *Fumicollis* are similarly shaped but more elongate. In *Baptornis* the proximal end is not as greatly expanded, and these phalanges are not as compressed mediolaterally. In *Parahesperornis,* a deep, triangular depression is present on the proximal end of the plantar surface, as in other hesperornithiforms. The proximal articular surface is oval shaped in proximal view, with the plantar end somewhat flattened and the dorsal end coming to a peak, with the surface deeply depressed, as in *Hesperornis* and *Fumicollis*. In *Baptornis,* the proximal end is more circular.

The distal ends of the digit III phalanges in *Parahesperornis* are divided into two similarly sized trochlea, the medial of which extends very slightly further distally than the lateral, as in *Hesperornis*. In *Baptornis* the trochleae are less clearly defined and have an equal distal extent. In distal view, the dorsoplantar axes of the trochleae intersect dorsally. The phalanges of digit III of *Parahesperornis* get progressively shorter away from the foot, as in other hesperornithiforms. The ungual phalanx, or claw, is small and triangular, flattened dorsoplantarly, and with a bulbous proximal end, as in *Hesperornis*.

The phalanges of digit II of *Parahesperornis* are highly elongate and fairly consistent along their length, widening slightly at the distal end in medial or lateral view and narrowing to a very slight neck just above the trochlea. A very faint groove runs along the dorsal surface. These phalanges are somewhat flattened dorsoplantarly, particularly the second phalanx. The proximal articular surface of the first phalanx is triangular and faintly divided into two cotylae for articulation with the uneven second trochlea of the tarsometatarsus. The distal end is divided into two subequal trochlea. The proximal surface of the second phalanx is narrower and oval in outline, with only faint margins of the cotylae. The distal trochleae of the second phalanx are only weakly developed. The ungual phalanx is much like that of the third toe, such that they seem interchangeable. The morphology of these phalanges is consistent with that of *Hesperornis* and *Fumicollis.*

The first phalanx of digit I of *Parahesperornis* is small and rectangular in dorsal or plantar view. It is highly flattened dorsoplantarly and slightly arched in medial or lateral view. Both the proximal and distal ends are weakly developed, with little development of the articular surfaces. The morphology of the first phalanx is very similar to that of *Fumicollis.* The ungual phalanx of the first toe is triangular and flattened dorsoplantarly and is slightly more elongate than the other unguals.

#### 3.2.7. Integument

**Figure 68:**
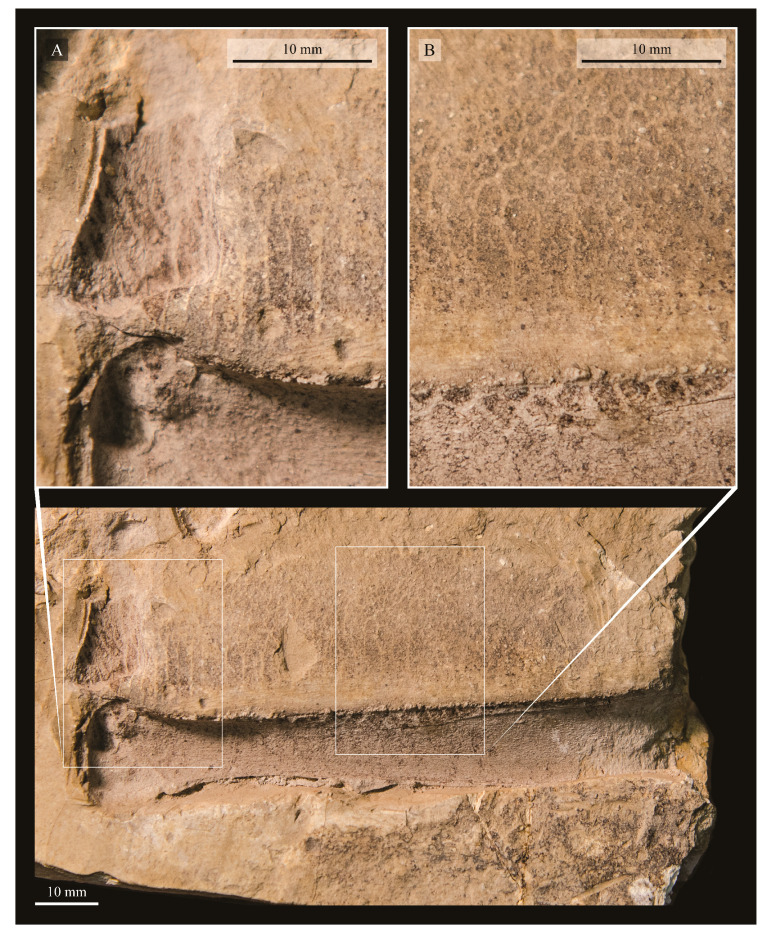
*Parahesperornis alexi* KUVP 2287, part of the slab preserving the impressions of the tarsometatarsi and a number of pedal phalanges with integument impressions, with details of scutes and scutellae (**A**,**B**).

**Figure 69:**
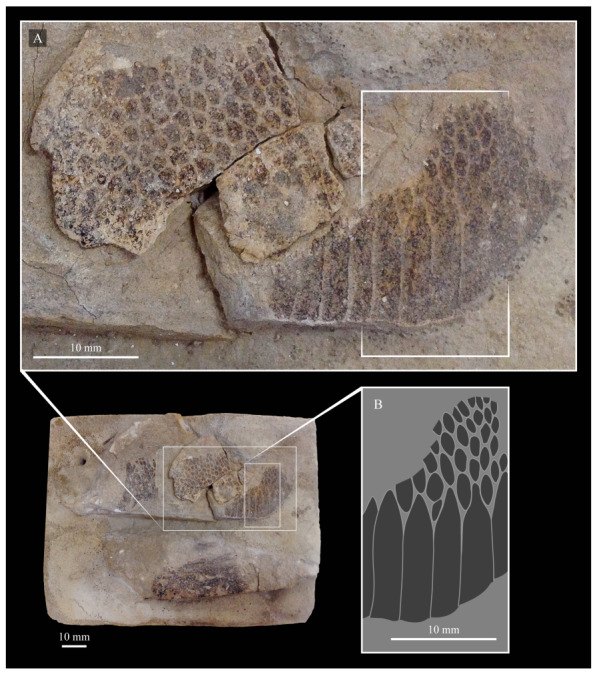
*Parahesperornis alexi* KUVP 2287, slab preserving integument impressions associated with the specimen, with details (**A**) and sketch (**B**) of scutes and scutellae. This is the same slab figured by Williston [53], and originally preserved the right tarsometatarsus.

**Figure 70:**
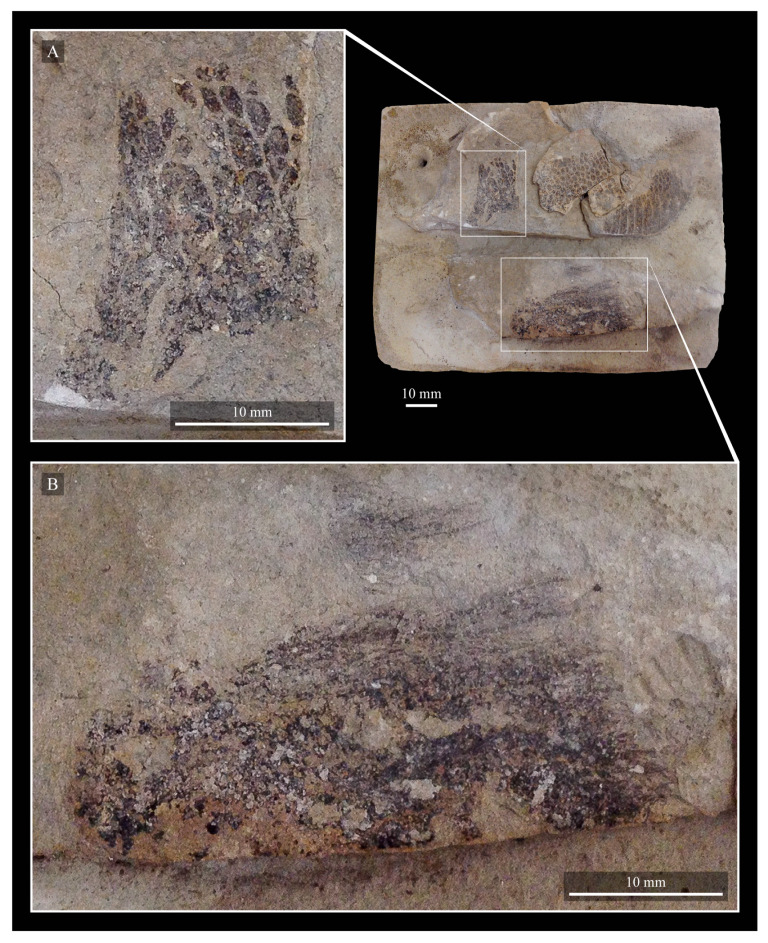
*Parahesperornis alexi* KUVP 2287, additional details from the slab in Figure 69 showing scutes and scutellae (**A**) as well as an indeterminate impression (**B**) interpreted by Williston [53] as feather impressions.

While soft tissue preservation of feathers and skin is common among avian specimens from the Early Cretaceous Jehol biota [88] and other Lagerstätten (e.g., Messel [89], Green River [90]), it is incredibly rare among hesperornithiforms. The holotype of *Parahesperornis alexi*, KUVP 2287, is the only hesperornithiform specimen for which skin impressions are known (Figure 68, Figure 69 and Figure 70). These impressions were first described in 1896 by Williston [53] and occur on two sections of matrix that were removed from the region of the feet during initial preparation of the specimen, one of which was figured by Williston [53] (Figure 69 and Figure 70).

The largest impressions occur as a rounded patch roughly 24 cm^2^ that overlaps a portion of the impression of the left tarsometatarsus and one of the pedal phalanges (Figure 68). The second set of impressions, while smaller in area, are clearer in outline and were removed from the area over the right tarsometatrsus [53] (Figure 69 and Figure 70). Most of these impressions appear to represent sections of skin, most likely from the foot, which sloughed off during decomposition and ended up preserved next to the tarsometatarsi. The impressions show a series of aligned and interlocking scutes of two types: small, equilateral scutellae and larger elongate scutes. The small equilateral scutellae are variable in their exact shape, ranging from nearly round to diamonds. The elongate scutes are more standard in their shape, forming highly elongate pentagons aligned parallel to each other. There does not appear to be much gradation between the smaller scutellae and the elongate scutes, and none of these overlap. The elongate scutes appear to form a single row with the equilateral scutellae stacked in off the triangular edge. This is typical of the pattern of scutes on modern bird feet, with the exception of the short, flat side visible in the elongate scutes. In modern birds these elongate scutes run mediolaterally across the dorsal surface of the tarsometatarsus and toes and are pointed on both ends. It is likely that the flat sides observed in these impressions is an effect of breakage and wear to the matrix.

The second impression of interest is found on the opposite side as the scutes on the matrix removed from over the right tarsometatarsus (Figure 70B). This impression was identified as feathers by Williston [53], who described them as, “clearly semiplumulaceous in character, the pennaceous shaft of considerable size, the vanes long and wavy.” It is likely it was this description that Marsh [6] later cited when he referred to a *Hesperornis* specimen with feathers. Williston [53] also indicated that impressions of “wavy vanes” were also present in matrix near the skull and elsewhere in KUVP 2287; however, those sections of matrix are not found with the specimen today. Despite Williston’s description, it is not possible to confirm his interpretation of feather impressions on this slab. This particular impression is indistinct, and actually appears to preserve a very small section of the equilateral scutellae (perhaps six of them) to the left of center; it is possible that the impressions may have degraded in the ensuing years since his original description.

## 4. Discussion

### 4.1. Comparative Morphology

As two of the best-preserved hesperornithiform specimens known, KUVP 2287 and KUVP 24090 provide a rare opportunity to observe the entire skeletal morphology of a derived, extinct diving bird, *Parahesperornis alexi*. The only other hesperornithiform with a similarly well-known skeleton is *Hesperornis regalis*, however in that case the completeness of the skeleton is the result of a composite of multiple specimens. The anatomical study undertaken in this paper shows that *Parahesperornis* is remarkably similar to *Hesperornis* in many ways, and yet retains some primitive features that indicate a less specialized morphology. This study therefore expands on the conclusions of the most comprehensive phylogenetic study of these birds, which found that *Parahesperornis* and *Hesperornis* formed the monophyletic Hesperornithidae [16]. This clade had the strongest support of any clade recovered, with its monophyly supported by 28 unambiguous synapomorphies [16]. These synapomorphies included the extreme curvature of the lateral margin of the femur, robust intramuscular scars on the femur, bulbous trochanter of the femur, pocketed patellar groove, elongate patella, groove across the distal fibular crest of the tibia, twisted tibiotarsal shaft, fourth metatarsal trochlea nearly twice as wide as the second and third trochleae, and a robust first phalanx of pedal digit IV with a deep plantar groove [16].

Many of these features have been associated with direct functional benefits for foot-propelled diving and are often convergently evolved in various clades of modern foot-propelled diving birds [18]. For example, the robust muscle scarring along the femur and hyperdevelopment of the femoral trochanter indicate strongly developed hindlimb musculature. The extreme splay of the femur as well as the rotated fibular condyle common in hesperornithiforms is also seen in modern loons and grebes. The extremely elongate patella, similar to that of modern grebes and some cormorants, provides an expanded area of attachment for the m. femorotibialis medialis, the m. extensor digitorum longus, and the m. tibialis cranialis, among others. These muscles function to stabilize the knee (m. femorotibialis medialis), extend the toes (m. extensor digitorum longus), and rotate the lower leg (m. tibialis cranialis), all motions that are crucial to foot-propelled diving [86]. The cnemial expansion of the tibiotarsus, also present in varying degrees in loons, grebes, and cormorants, is a functionally significant structure that serves as the origin of muscles that control foot motion [18] and also allows for the rotation of the tibiotarsus [91]. The similarities of the cnemial expansion across hesperornithiforms may indicate stabilizing selection due to the significance of this structure in foot-propelled diving. Similarly, the twisting of the shafts of the tibiotarsus and tarsometatarsus described here for hesperornithids would have further contributed to the range of motion of the feet achieved by the rotation of the lower legs. This is a feature not seen among modern foot-propelled diving birds.

However, despite the strongly supported phylogentic relationship between *Parahesperornis* and *Hesperonis*, *Parahesperornis* retains a number of primitive features that indicate it was not as highly derived as *Hesperornis*. Some of these features include the greater distal extent of the lateral condyle of the femur (as compared to the medial condyle), the lack of expansion of the lateral condyle of the tibiotarsus, the low relief of the intercotylar eminence of the tarsometatarsus, and the only slightly more distally extended trochlea IV of the tarsometatarsus (as compared to trochlea III). Some of these features also have direct correlates with foot-propelled diving capabilities. For example, the smaller lateral condyle of the tibiotarsus would have reduced the available attachment area for the ligament collaterale laterale intertarsi, which helps stabilize the intertarsal joint [86]. Additionally, almost the entire surface of the intercotylar eminence is taken up by the ligamenta anticum, which functions to stabilize the intertarsal joint during rotation of the tarsometatarsus [86]. By lacking the stronger development of these features displayed by *Hesperornis*, *Parahesperornis* may not have been as advanced a foot-propelled diver.

These features are independent of body size, indicating that the smaller species of *Hesperornis*, which were similarly sized or even smaller than *Parahesperornis*, still possessed more advanced diving morphologies. For example, *Hesperornis macdonaldi*, the smallest species of *Hesperornis*, is known from the holotype, an isolated femur from the Pierre Shale of South Dakota (holotype, LACM 9728) [33], as well as five assigned femora from the Pierre Shale of Manitoba, Canada [32] and an additional unreported femur in the collections of the LACM also from South Dakota (LACM 9727). The morphology of these elements is consistent with that of fully-grown individuals; however, this ontogenetic interpretation has not been confirmed with histologic data.

Despite its small size, several morphological features of the femur of *H. macdonaldi* are consistent with more advanced diving adaptations, such as similar distal extents of the lateral and medial condyles. This is a synapomorphy of the species of *Hesperornis*. Regarding this feature, *Parahesperornis* is intermediate between the even condyles of *Hesperornis* and the primitive condition in other hesperornithiforms such as *Baptornis* and *Enaliornis,* where the lateral condyle extends markedly beyond the medial condyle. Functionally, the uneven condyles of the more basal hesperornithiform taxa affect the orientation of the tibiotarsus and therefore, the placement of the foot behind the body. The even condyles of *Hesperornis* allow the tibiotarsus to extend in a straight line behind the femur and the body (i.e., directly caudally), while in the more basal taxa the tibiotarsus would have angled down ventrally below the body. Thus, *Hesperornis* was able to position its feet directly behind the body, increasing efficiency and decreasing drag.

Another example of the decoupling of size and diving function is found in *Hesperornis mengeli*, another small species of *Hesperornis* known from an isolated tarsometatarsus from the Pierre Shale of Manitoba, Canada [33]. *H. mengeli* (CFDC B.78.01.08 [32], contra Martin and Lim [33]) is 85% the length of *Parahesperornis* (based on tarsometatarsal lengths), yet has a well-defined, sharp intercotylar eminence like that of other species of *Hesperornis* and very different from the reduced, rounded shape found in *Parahesperornis*. The same is true of *H. lumgairi*, also known from a tarsometatarsus from the Pierre Shale of Manitoba [32], which is only slightly larger than *Parahesperornis* but has a dramatic, peaked intercotylar eminence.

While the exact role of the intercotylar eminence in diving performance has not been clearly established, in *Hesperornis*, as in modern loons, the intertarsal joint is stabilized by the anticum ligament, which attaches along the cranial edge of the intercotylar eminence [86]. By elongating the intercotylar eminence, the attachment area for this ligament may be increased, thus better stabilizing the joint for force production while swimming.

One interesting difference between *Hesperornis* and *Parahesperornis* that may further support stronger diving capabilities in *Hesperornis* is the extreme difference in the overall shape of the skull. *Parahesperornis* has a short, robust skull as compared to *Hesperornis*, which has a much more elongate skull. This is seen in three regions of the skull: the parietals and temporal fenestrae, the frontals, and the pre-nares portion of the rostrum. While the parietals are poorly preserved in *Parahesperornis* KUVP 2287, the length of the temporal fenestra of both *Parahesperornis* and *Hesperornis* is markedly longer than in *Enaliornis* (Figure 9), thus demonstrating the elongation of hesperornithids compared to the basal *Enaliornis.* However, the temporal fenestra of *Hesperornis* is proportionally longer than that of *Parahesperornis* (Figure 9D). Additionally, the frontals are far more robust and less expanded anteriorly in *Parahesperornis* than in *Hesperornis*. In *Parahesperornis* the width of the frontals at the widest point behind the orbit is roughly 75% the length of the frontals, while in *Hesperornis* the width is 58% the length, with both specimens appearing to preserve similar portions of this bone. The premaxilla is also proportionally shorter in *Parahesperornis*, where the mediolateral width at the rostral extent of the nares is roughly 35% the rostrocaudal length of the premaxillae from the nares to the tip of the bone, while in *Hesperornis*, it is 20%. Also in *Parahesperornis*, the frontals and rostral premaxillae (from the rostral extent of the nares to the tip) are similar lengths, while in *Hesperornis* the frontals are only about 80% this length. These differences in proportion indicate a much boxier, more robust skull in *Parahesperornis*, a condition that likely reflects functional differences (e.g., trophic, hydrodynamic, etc.), and may have played a role in the overall streamlining of the body while diving.

Here we describe a difference in the construction of the neck between *Parahesperornis* and *Hesperornis,* with the transition to thoracic-style vertebrae occurring at the 16th vertebra in *Parahesperornis* and at the 17th in *Hesperornis*. The diversity of vertebral morphology among modern birds is striking and very different from the conserved pattern seen in mammals. Among modern birds the number of cervical vertebrae, for example, ranges from ten (parrots) to 26 (swans), with a median of 13 [92]; hesperornithiforms, with 16–17 cervical vertebra, thus fall to the higher end of this spectrum. To further complicate this, neck elongation in modern birds is sometimes achieved through elongation of a few individual vertebrae (i.e., flamingos) or through the addition of several short vertebrae (i.e., swans). This is also very different from mammals, most of which have seven cervical vertebrae regardless of neck length (e.g., giraffes and humans). Cervical count alone, therefore, is not sufficient to characterize the functional capabilities of a bird’s neck [92].

Therefore, the evolution and development of the neck of hesperornithiforms may play a significant role in better understanding the transition to aquatic life undertaken by this lineage. Variations in the ontogenetic development leading to the diversity of neck morphologies in modern birds have not been well studied. Somitogenesis is responsible for the formation of the individual somites that develop into vertebrae, with rate of development varying across modern avian taxa [93]. Heterochrony may therefore account for the homeotic shift between *Parahesperornis* and *Hesperornis,* whereby the transition to thoracic-style vertebra happened one vertebra later in *Hesperornis* than in *Parahesperornis*, perhaps indicating an increased duration of somitogenesis.

An interesting similarity between *Hesperornis*, *Parahesperornis*, and *Baptornis* is the shared reduction of the forelimb, with these birds showing similar reductions in humerus and shoulder development, as compared to *Pasquiaornis*, a more basal hesperornithiform in which the morphology and proportions of the humerus and the shoulder are not nearly as reduced, despite *Pasquiaornis* showing some clear diving adaptations in the hindlimb. While *Hesperornis*, *Parahesperornis*, and *Baptornis* differ in inferred diving capabilities (see Section 4.2 below), these birds exhibit similar extreme forelimb reduction associated with the loss of flight.

### 4.2. Ecology of Parahesperornis

Foot-propelled diving has evolved numerous times in both modern and extinct birds. Among modern birds, there are five main groups of foot-propelled divers: loons (Gaviidae), grebes (Podicipedidae), cormorants (Phalacrocoracidae), some ducks (Anseridae), and coots (Rallidae). Molecular phylogenies of modern birds consistently identify these groups as not closely related [94,95,96], indicating foot-propelled diving evolved convergently among these groups. This is further supported by differences in their skeletal morphology and foraging behavior.

Bell et al.’s [18] recent quantitative morphometric comparison of hesperornithiform taxa with modern foot-propelled divers has highlighted key differences in morphological adaptations that indicate the various strategies modern and extinct lineages employed as they convergently evolved foot-propelled diving [18]. Among modern foot-propelled divers, key differences are the enlargement or reduction of the patella and the cnemial expansion of the tibiotarsus, robustness versus elongation of the femur, and the ratio of femur length to both tibiotarsus and tarsometatarsus length [18]. Bell et al.’s [18] study has shown that while some adaptations are common across all foot-propelled divers, in many of these features hesperornithiforms are more similar to cormorants and diving ducks than to loons or grebes, thus challenging the decades-old conventional notion that hesperornithiforms were most similar to the latter two groups of modern birds [7,13,15,97].

*Parahesperornis* possesses a variety of skeletal features present in these different groups of modern divers. The elongate patella of *Parahesperornis* is most like that of grebes [18], whereas other divers like loons have extreme reduction of the patella, such that it consists of a small flake of bone embedded in the patellar tendon [98]. Alternatively, the intercotylar eminence of the tarsometatarsus of *Parahesperornis* most closely resembles that of loons, which is lower and more rounded than that of grebes [18]. Most of these modern diving families have members that have specialized to the point of flightlessness [99], as did *Parahesperornis* and the other hesperornithiforms. Flightlessness in diving birds is likely the result of the conflicting physiological demands of underwater diving and aerial flight; whereas underwater diving benefits from increased blood volume and decreased respiratory volume in order to counter buoyancy, the opposite is true of aerial flight [100].

The extreme reduction of the forelimb shared between *Hesperornis*, *Parahesperornis*, and *Baptornis*, may indicate that loss of flight occurred early in the hesperornithiform lineage, followed by diversification in hindlimb morphology, which may have been associated with niche partitioning or other ecological specialization. This is supported by the morphology of *Pasquiaornis*, a basal hesperornithiform, that has a less reduced forelimb as well as fewer adaptations for foot-propelled diving. For example, *Pasquiaornis* has an elongate femur only slightly expanded proximally and distally expansions typical in more derived.

The avian neck is fascinating in its diversity, which is analogous to the mammal arm in terms of both diversity of form and function. The neck of birds plays an important role in specialized ecological tasks, such as feeding, head stabilization during locomotion, object manipulation and nest building, preening, sexual displays, and aggressive behaviors [92]. While neck length has shown to be of limited utility in predicting ecological specializations in modern birds, one exception would be the general pattern that longer-necked birds tend to have some variety of aquatic lifestyle (waders, dabblers, divers, etc.) [92]. Modern foot-propelled diving birds use their elongate necks to increase maneuverability underwater. For example, cormorants have been documented to move their head and neck independently of the body during pursuit diving, thus avoiding limitations imposed by the limited turning radius of the entire body [101]. Foot-propelled diving birds that swim with their long necks extended have lower drag coefficients than wing-propelled divers that swim with their necks retracted [91]. Expanding on this, studies have shown that cormorants swimming with outstretched necks have low drag coefficients at high speeds but higher drag coefficients at low speeds, while the opposite is true of penguins, which have short necks and swim with them retracted [102]. The earlier transition to the thoracic vertebral morphology in the 16th cervical of *Parahesperornis* may indicate a functionally shorter neck and imply higher maneuverability in *Hesperornis.*

It is interesting to note that among modern diving birds, foot-propelled divers such as loons, grebes, cormorants, and diving ducks are generally found in shallow, primarily freshwater or estuarine environments, while pelagic marine divers are primarily wing-propelled birds like the alcids, diving petrels, and penguins [100]. This represents a dichotomy between shallow and deep-water diving, with wing-propelled divers typically engaging in much deeper dives than foot-propelled divers. There are exceptions to this trend, with some members of the Alcidae specializing on shallow mussel beds while some cormorant species practice deep dives of up to 100 meters [100]. The exceptions to this general observation break down along the lines of ecology among foot propelled divers, with pursuit divers such as the great cormorant (*Phalcrocorax carbo*) and the common loon (*Gavia immer*) diving to much greater depths than benthivorous divers such as pochard and scaup ducks (i.e., *Aythyinae*) [100].

The fossil record of hesperornithiforms includes a variety of depositional environments, from continental freshwater to marine, as previously discussed [18]. While the environment of deposition is not always the same as the environment in which the organism lived, taphonomy tends to result in the deposition of remains in deeper water, not shallower water, than that in which the organism lived. Therefore, the absence of *Parahesperornis* and of most other large-bodied hesperornithiforms in continental deposits may indicate *Parahesperornis* did not occupy these environments and was instead a marine diver [18].

Body size also plays an important role in diving behavior, as larger bodied birds tend to be deeper divers, and face a different set of biomechanical and physiological trade-offs than do shallow divers [100]. As compared to modern divers, *Parahesperornis* (and most species of *Hesperornis*) were massive. Body size has a very strong effect on drag, which is the primary mechanical cost of steady swimming at high depths [102]. In general, larger body size correlates with a lower mass-specific metabolic rate and greater oxygen stores, all of which enhance underwater diving capabilities [103]. Furthermore, the overall shape of a bird’s body can influence drag. Our understanding of the skeletal morphology of *Parahesperornis* indicates that the overall body shape of this bird would have closely resembled that of a cormorant, but larger, and so it seems ideally adapted for high speed pursuit diving at great depths. This relates to the variation in skull shape between *Hesperornis* and *Parahesperornis*, with the elongate skull of *Hesperornis* reducing drag more than the squat skull of *Parahesperornis*.

The effect of feather type on the drag coefficient can also be significant, with penguin feathers having the lowest drag coefficient of modern feathers [102]. Unfortunately, the lack of evidence for feather type in *Parahesperornis* (and all other hesperornithiforms) makes this aspect of diving impossible to evaluate. However, many of the features of feathers that make them poorly suited for reducing drag (for example, long flight feathers) are necessary for a flighted bird [100], but not in a flightless bird such as *Parahesperornis*. Therefore, it is possible that feather adaptations, such as those seen in penguins, could have further increased the diving capabilities of hesperornithiforms.

### 4.3. Other Specimens of Parahesperornis

In addition to the specimens assigned to *Parahesperornis* discussed here, previous work has proposed other specimens as possibly belonging to the genus. Kurochkin [28] referenced and illustrated a distal tibiotarsus belonging to a small hesperornithiform collected from the Nemegt Formation of Tsagaan Khushuu, Omnogov’ Aimag, Mongolia. While initially described as belonging to the genus *Baptornis* [28], Kurochkin noted the following features as indicative of a bird more similar to *Parahesperornis*: (1) similarity of the lateral and medial condyles; (2) no distal projection of the medial condyle; and (3) medial position of the extensor groove. The distal transverse width of the specimen was listed as 11.7 mm [28], while that of the holotype KUVP 2287 is 23.38 mm and KUVP 24090 is 24.59 mm, and so on the basis of size alone the Mongolian specimen, assuming it is an adult, does not appear consistent with *Parahesperornis alexi*.

In terms of the morphological features of the Mongolian tibiotarsus, the similarity of the lateral and medial condyles Kurochkin [28] notes may be more indicative of *Baptornis*, as in both *Hesperornis* and *Parahesperornis* the lateral and medial condyles of the tibiotarsus are very differently shaped when viewed from the sides. The lack of distal projection of the medial condyle is also seen in *Baptornis* and *Hesperornis* (Figure 63), therefore this condition is not diagnostic of *Parahesperornis*. The extensor groove is shifted medially in *Parahesperornis*, as Kurochkin [28] described for the Mongolian specimen. This, and the medial expansion of the medial condyle of the tibiotarsus in cranial view, appears to be the strongest support for identification of the Mongolian specimen as *Parahesperornis*. However, other features, such as the deeper groove between the epicondyles on the caudal surface of the shaft, indicate differences between the Mongolian specimen and *Parahesperornis alexi*. Kurochkin [28] did not indicate where the specimen is held, or provide a specimen number, so further work may prove difficult.

More recently, Zelenkov et al. [44] proposed that *Asiahesperornis* may, in fact, be a junior synonym of *Parahesperornis*. This was based on the similarity of the proximal tarsometatarsi, both of which have a mediolaterally compressed medial cotyla, such that the overall shape of the proximal surface is rhombic rather than rectangular in proximal view [44]. Zelenkov et al. [44] noted this shape in *H. crassipes* and *H. chowi* as well and indicated that perhaps all four taxa should be synonymized with *Parahesperornis*.

The use of this single trait as a means of combining four current taxa appears unwarranted at this time, particularly in light of the presence of a mosaic of traits in *Parahesperornis* which individually are found in other hesperornithiform taxa. In addition to being slightly larger than *Parahesperornis, Asiahesperornis* is diagnosed by the presence of distinct grooves on the dorsal surface of the tarsometatarsus shaft [80], a trait also seen in *Hesperornis*. While present in *Parahesperornis*, these grooves are faint proximally, much more so than in *Asiahesperornis*, which is potentially even more deeply grooved than in *Hesperornis*. Furthermore, both *H. crassipes* and *H. chowi* show a number of typical *Hesperornis* features, such as the sharp and expanded intercotylar eminence, greatly expanded trochlea IV, and pronounced groove between metatarsals III and IV. Despite the similarity of the proximal outline of the tarsometatarsus, these genera should not be considered synonymous with *Parahesperornis*.

## 5. Conclusions

The detailed anatomical study presented here fully illustrates the transitional skeletal morphology of *Parahesperornis alexi* that combines characters found in more basal taxa with others characteristic of the highly specialized *Hesperornis*. While strongly resolved as the sister-taxon to the well-known *Hesperornis* [16], *Parahesperornis* retains a number of traits that indicate it may have lagged behind *Hesperornis* in diving capabilities such as maneuverability, speed, and depth. In addition to the better-known differences of the hindlimb between *Parahesperornis* and *Hesperornis*, our study highlights important cranial differences that may have also played a role in their differential hydrodynamic properties, and possibly, their ecology. The unique skeletal anatomy clearly indicates that no modern diving bird can be fully regarded as a functional analog of the hesperornithiforms, thus underscoring the diversity of diving strategies among modern and extinct foot-propelled divers. That said, given the much larger body size and more specialized structure of the hindlimb, it is likely that *Parahesperornis* would have outperformed modern foot-propelled divers underwater.

The relevance of the Hesperornithiformes to understanding the origin and evolution of modern birds has all too often been overlooked in favor of the story of their loss of flight and transition to a fully aquatic lifestyle. While this evolutionary progression is fascinating, the potential contribution of hesperornithiforms to our understanding of the evolution of modern birds has yet to be fully realized. This study clarifies a number of morphological features of hesperornithiforms that are significant for phylogenetic analysis, in particular to understanding the origin of modern birds, given the phylogenetic position of hesperornithiforms as the sister clade of modern birds.

The present study, for the first time, fully illustrates numerous morphological features of hesperornithiforms, allowing full comparisons across a variety of genera. As one of the best-preserved hesperornithiforms, *Parahesperornis* contributes a great deal to our understanding of the Hesperornithiformes as a whole. By comparing and fully illustrating the only two species of hesperornithiforms, *Parahesperornis alexi* and *Hesperornis regalis*, for which nearly all skeletal elements are known, this study allows broad evolutionary patterns as well as interclade comparisons to be made. From this, we see the story of the Hesperornithiformes to be one of not only the loss of flight during the transition to a highly derived aquatic lifestyle, but also the story of the very last stages of pre-modern bird evolution.

## Supplementary Materials

The following are available online at https://www.mdpi.com/2075-1729/10/5/62/s1, Table S1: Collected measurements of *Parhesperornis alexi* and other hesperornithiform birds. Measurements were collected from the specimens noted using digital calipers, unless otherwise noted. Table S2: Hesperornithiform specimens used for comparison in this study.

## References

[1] Gauthier J. (1986). Saurischian Monophyly and the Origin of Birds. Mem. Calif. Acad. Sci..

[2] Clarke J.A. (2004). Morphology, Phylogenetic Taxonomy, and Systematics of *Ichthyornis* and *Apatornis* (Avialae: Ornithurae). Bull. Am. Mus. Nat. Hist..

[3] Seeley H.G. (1876). On the British Fossil Cretaceous Birds. Q. J. Geol. Soc..

[4] Marsh O. (1872). XXXII.—Description of *Hesperornis regalis*, with notices of four other new species of Cretaceous birds. J. Nat. Hist..

[5] Marsh O.C. (1876). Notice of new Odontornithes. Am. J. Sci..

[6] Marsh O.C. (1877). Characters of the Odontornithes, with notice of a new allied genus. Am. J. Sci..

[7] Marsh O.C. (1880). Odontornithes: A Monograph on the Extinct Toothed Birds of North America.

[8] Shufeldt R.W. (1915). The Fossil Remains of a Species of *Hesperornis* Found in Montana. Auk.

[9] Martin L., Tate J.A. (1967). A *Hesperornis* from the Pierre Shale. Neb. Acad. Sci. Proc..

[10] Fox R.C. (1974). A Middle Campanian, Nonmarine Occurrence of the Cretaceous Toothed Bird *Hesperornis* Marsh. Can. J. Earth Sci..

[11] Cumbaa S.L., Schröder-Adams C., Day R.G., Phillips A.J., Lucas S., Sullivan R. (2006). Cenomanian Bonebed Faunas from the Northeastern Margin, Western Interior Seaway, Canada. Late Cretaceous Vertebrates from the Western Interior. New Mexico Museum of Natural History and Science Bulletin 35.

[12] Tanaka T., Kobayashi Y., Kurihara K., Fiorillo A.R., Kano M. (2017). The oldest Asian hesperornithiform from the Upper Cretaceous of Japan, and the phylogenetic reassessment of Hesperornithiformes. J. Syst. Palaeontol..

[13] Lucas F.A. (1903). Notes on the osteology and relationship of the fossil birds of the genera *Hesperornis, Hargeria*, *Baptornis*, and *Diatryma*. Proc. U. S. Natl. Mus..

[14] Storer R.W. Evolution in the Diving Birds. Proceedings of the XII International Ornithological Congress Helsinki: Tilgmannin Kirjapaino.

[15] Cracraft J. (1982). Phylogenetic Relationships and Monophyly of Loons, Grebes, and Hesperornithiform Birds, with Comments on the Early History of Birds. Syst. Zool..

[16] Bell A., Chiappe L. (2015). A species-level phylogeny of the Cretaceous Hesperornithiformes (Aves: Ornithuromorpha): Implications for body size evolution amongst the earliest diving birds. J. Syst. Palaeontol..

[17] Wilson L.E., Chin K. (2014). Comparative osteohistology of *Hesperornis* with reference to pygoscelid penguins: The effects of climate and behaviour on avian bone microstructure. R. Soc. Open Sci..

[18] Bell A., Wu Y.-H., Chiappe L. (2019). Morphometric comparison of the Hesperornithiformes and modern diving birds. Palaeogeogr. Palaeoclim. Palaeoecol..

[19] Martin L.D., Tate J. (1976). The Skeleton of *Baptornis advenus* (Aves: Hesperornithiformes). Smithson. Contrib. Paleobiol..

[20] Bell A., Chiappe L. (2015). Identification of a New Hesperornithiform from the Cretaceous Niobrara Chalk and Implications for Ecologic Diversity among Early Diving Birds. PLoS ONE.

[21] Galton P.M., Martin L.D. (2002). Postcranial Anatomy and Systematics of *Enaliornis* SEELEY, 1876, a Foot-Propelled Diving Bird (Aves: Ornithurae: Hesperornithiformes) from the Early Cretaceous of England. Rev Paleobiol..

[22] Tokaryk T.T., Harington C.R. (1992). *Baptornis* sp. (Aves: Hesperornithiformes) from the Judith River Formation (Campanian) of Saskatchewan, Canada. J. Paléontol..

[23] Rees J., Lindgren J. (2005). Aquatic Birds from the Upper Cretaceous (Lower Campanian) of Sweden and the Biology and Distribution of Hesperornithiforms: Cretaceous Aquatic Birds. Palaeontology.

[24] Eberth D., Brinkman D.B. (1997). Paleoecology of an Estuarine, Incised-Valley Fill in the Dinosaur Park Formation (Judith River Group, Upper Cretaceous) of Southern Alberta, Canada. PALAIOS.

[25] Nessov L.A., Borkin J. (1983). New Records of Bird Bones from the Cretaceous of Mongolia and Soviet Middle Asia. USSR Acad. Sci. Proc. Zool. Inst..

[26] Bell A., Everhart M.J. (2009). A New Specimen of *Parahesperornis* (Aves: Hesperornithiformes) from the Smoky Hill Chalk (Early Campanian) of Western Kansas. Trans. Kans. Acad. Sci..

[27] Everhart M.J., Bell A. (2009). A hesperornithiform limb bone from the Basal Greenhorn Formation (Late Cretaceous; Middle Cenomanian) of North Central Kansas. J. Vertebr. Paléontol..

[28] Kurochkin E.N. (2000). Mesozoic Birds of Mongolia and the Former USSR. Age Dinosaurs Russ. Mong..

[29] Tokaryk T.T., Cumbaa S.L., Storer J.E. (1997). Early Late Cretaceous birds from Saskatchewan, Canada: The oldest diverse avifauna known from North America. J. Vertebr. Paléontol..

[30] Martin L.D., Kurochkin E.N., Tokaryk T.T. (2012). A new evolutionary lineage of diving birds from the Late Cretaceous of North America and Asia. Palaeoworld.

[31] Martin J.E., Cordes-Person A. (2007). A new species of the diving bird *Baptornis* (Ornithurae: Hesperornithiformes) from the lower Pierre Shale Group (Upper Cretaceous) of southwestern South Dakota. Geol. Paleontol. Late Cretac. Mar. Depos. Dakotas.

[32] Aotsuka K., Sato T. (2016). Hesperornithiformes (Aves: Ornithurae) from the Upper Cretaceous Pierre Shale, Southern Manitoba, Canada. Cretac. Res..

[33] Martin L.D., Lim J.-D. (2002). New Information on the Hesperornithiform Radiation. Proceedings of the 5th Symposium of the Society of Avian Paleontology and Evolution.

[34] MacDonald J. (1951). The Fossil Vertebrata of South Dakota. Guidebook, Fifth Field Conference of the Society of Vertebrate Paleontology, West South Dakota.

[35] Bardack D. (1968). Fossil vertebrates from the marine Cretaceous of Manitoba. Can. J. Earth Sci..

[36] Bryant L.J. (1983). *Hesperornis* in Alaska. PaleoBios.

[37] Hills L.Y., Nicholls E.L., Nunez-Betelu M., Mcintyre D.J. (1999). *Hesperornis* (Aves) from Ellesmere Island and Palynological Correlation of Known Canadian Localities. Earth Sci..

[38] Case G. (1978). News from Members, Eastern Region, Jersey City. Soc. Vertebr. Paleontol. News Bull..

[39] Bell A., Irwin K.J., Davis L.C. (2015). Hesperornithiform Birds from the Late Cretaceous (Campanian) of Arkansas, USA. Trans. Kans. Acad. Sci..

[40] Martin L.D. (1984). A New Hesperornithid and the Relationships of the Mesozoic Birds. Trans. Kans. Acad. Sci..

[41] Marsh O.C. (1893). A new Cretaceous bird allied to *Hesperornis*. Am. J. Sci..

[42] Martin J.E., Varner D.W. (1992). The Occurrence of *Hesperornis* in the Late Cretaceous Niobrara Formation of South Dakota. Proc. SD Acad. Sci..

[43] Hilton R. (2003). Dinosaurs and Other Mesozoic Reptiles of California.

[44] Zelenkov N.V., Panteleyev A.V., Yarkov A.A. (2017). New finds of hesperornithids in the European Russia, with comments on the systematics of Eurasian Hesperornithidae. Paléontol. J..

[45] Tokaryk T.T. (1999). A Toothed Bird *Hesperornis* sp. (Hesperornithiformes) from the Pierre Shale (Late Cretaceous) of Saskatchewan. Can. Field Nat..

[46] Nessov L.A., Prizemlin B. (1991). A Large Advanced Flightless Marine Bird of the Order Hesperornithiformes of the Late Senonian of Turgai Strait-the First Finding of the Group in the USSR. Tr. Zool. Instituta SSSR.

[47] Hou L.-I. (1999). New Hesperornithid (Aves) from the Canadian Arctic. Vertebr. PalAsiat..

[48] Wilson L.E., Chin K., Cumbaa S., Dyke G. (2011). A high latitude hesperornithiform (Aves) from Devon Island: Palaeobiogeography and size distribution of North American hesperornithiforms. J. Syst. Palaeontol..

[49] Elzanowski A., Paul G.S., Stidham T.A. (2001). An avian quadrate from the Late Cretaceous Lance Formation of Wyoming. J. Vertebr. Paléontol..

[50] Pearson D.A., Schaefer T., Johnson K.R., Nichols D.J., Hunter J.P. (2002). Vertebrate biostratigraphy of the Hell Creek Formation in southwestern North Dakota and northwestern South Dakota. The Hell Creek Formation and the Cretaceous-Tertiary Boundary in the Northern Great Plains: An Integrated Continental Record of the End of the Cretaceous.

[51] Tanaka T., Kobayashi Y., Ikuno K., Ikeda T., Saegusa H. (2020). A Marine Hesperornithiform (Avialae: Ornithuromorpha) from the Maastrichtian of Japan: Implications for the Paleoecological Diversity of the Earliest Diving Birds in the End of the Cretaceous. Cretac. Res..

[52] Scotese C.R. (2001). Atlas of Earth History.

[53] Williston S.W. (1896). On the Dermal Covering of Hesperornis. Kans. Univ. Q..

[54] Williston S.W. (1898). Birds. Univ. Geol. Surv. Kans..

[55] ICZN (1999). International Commission on Zoological Nomenclature.

[56] Elzanowski A. (1991). New Observations on the Skull of *Hesperornis* with Reconstructions of the Bony Palate and Otic Region. Postila.

[57] Bühler P., Martin L.D., Witmer L.M. (1988). Cranial Kinesis in the Late Cretaceous Birds *Hesperornis* and *Parahesperornis*. Auk.

[58] Gingerich P.D. (1973). Skull of *Hesperornis* and Early Evolution of Birds. Nature.

[59] Gregory J.T. (1952). The Jaws of the Cretaceous Toothed Birds, *Ichthyornis* and *Hesperornis*. Condor.

[60] O’Connor J., Chiappe L., Bell A. (2011). Pre-Modern Birds: Avian Divergences in the Mesozoic. Living Dinosaurs: The Evolutionary History of Modern Birds.

[61] Field D.J., Hanson M., Burnham D., Wilson L.E., Super K., Ehret D., Ebersole J.A., Bhullar B.S. (2018). Complete *Ichthyornis* skull illuminates mosaic assembly of the avian head. Nature.

[62] Chiappe L., Qingjin M., Serrano F.J., Sigurdsen T., Wang M., Bell A., Di L. (2019). New *Bohaiornis*-like bird from the Early Cretaceous of China: Enantiornithine interrelationships and flight performance. PeerJ.

[63] Wang Y.-M., O’Connor J.K., Li D.-Q., You H.-L. (2013). Previously Unrecognized Ornithuromorph Bird Diversity in the Early Cretaceous Changma Basin, Gansu Province, Northwestern China. PLoS ONE.

[64] Wang Y.-M., O’Connor J., Li D.-Q., You H.-L. (2015). New information on postcranial skeleton of the Early Cretaceous *Gansus yumenensis* (Aves: Ornithuromorpha). Hist. Biol..

[65] Sanchez J. (2018). Late Cretaceous (Cenomanian) Hesperornithiformes from the Pasquia Hills, Saskatchewan, Canada. Master’s Thesis.

[66] Bell A.K. (2013). Evolution and Ecology of Mesozoic Birds: A Case Study of the Derived Hesperornithiformes and the Use of Morphometric Data in Quantifying Avian Paleoecology. Master’s Thesis.

[67] Baumel J.J. (1993). Handbook of Avian Anatomy: Nomina Anatomica Avium.

[68] Everhart M.J. (2011). Rediscovery of the *Hesperornis regalis* Marsh 1871 Holotype Locality Indicates an Earlier Stratigraphic Occurrence. Trans. Kans. Acad. Sci..

[69] Gingerich P.D. (1976). Evolutionary Significance of the Mesozoic Toothed Birds. Smithson. Contrib. Paleobiol..

[70] Witmer L.M., Martin L.D. (1987). The Primitive Features of the Avian Palate, with Special Reference to Mesozoic Birds. Doc. Lab. Géologie Lyon.

[71] Elzanowski A. (1999). A Comparison of the Jaw Skeleton in Theropods and Birds, with a Description of the Palate in the Oviraptoridae. Smithson. Contrib. Paleobiol..

[72] Elzanowski A., Galton P.M. (1991). Braincase of *Enaliornis*, an Early Cretaceous bird from England. J. Vertebr. Paléontol..

[73] Martin L.D., Naples V.L. (2008). Mandibular Kinesis in *Hesperornis*. Oryctos.

[74] Smith N.A. (2011). Taxonomic revision and phylogenetic analysis of the flightless Mancallinae (Aves, Pan-Alcidae). ZooKeys.

[75] Witmer L.M. (1990). The craniofacial air sac system of Mesozoic birds (Aves). Zool. J. Linn. Soc..

[76] O’Connor J.K., Wang M., Hu H. (2016). A new ornithuromorph (Aves) with an elongate rostrum from the Jehol Biota, and the early evolution of rostralization in birds. J. Syst. Palaeontol..

[77] Rauhut O.W.M., Foth C., Tischlinger H. (2018). The oldest *Archaeopteryx* (Theropoda: Avialiae): A new specimen from the Kimmeridgian/Tithonian boundary of Schamhaupten, Bavaria. PeerJ.

[78] Donatelli R. (2012). Jaw Musculature of the *Picini* (Aves: Piciformes: Picidae). Int. J. Zool..

[79] Huang J., Wang X., Hu Y., Liu J., Peteya J.A., Clarke J.A. (2016). A new ornithurine from the Early Cretaceous of China sheds light on the evolution of early ecological and cranial diversity in birds. PeerJ.

[80] Dyke G., Malakhov D., Chiappe L. (2006). A re-analysis of the marine bird *Asiahesperornis* from northern Kazakhstan. Cretac. Res..

[81] Dumont M., Tafforeau P., Bertin T., Bhullar B.-A., Field D.J., Schulp A.S., Strilisky B., Thivichon-Prince B., Viriot L., Louchart A. (2016). Synchrotron imaging of dentition provides insights into the biology of *Hesperornis* and *Ichthyornis*, the "last" toothed birds. BMC Evol. Biol..

[82] Jenkins K.M., Jones M.E.H., Zikmund T., Boyde A., Daza J.D. (2017). A Review of Tooth Implantation Among Rhynchocephalians (Lepidosauria). S. Am. J. Herpetol..

[83] Martin L.D. (1987). The Beginning of the Modern Avian Radiation. Doc. Lab. Géologie Lyon.

[84] Zhou Z., Martin L.D. (2011). Distribution of the predentary bone in Mesozoic ornithurine birds. J. Syst. Palaeontol..

[85] Bailleul A.M., Li Z., O’Connor J., Zhou Z. (2019). Origin of the avian predentary and evidence of a unique form of cranial kinesis in Cretaceous ornithuromorphs. Proc. Natl. Acad. Sci. USA.

[86] Zinoviev A. (2011). Notes on the hindlimb myology and syndesmology of the Mesozoic toothed bird *Hesperornis regalis* (Aves: Hesperornithiformes). J. Syst. Palaeontol..

[87] Johansson L.C., Norberg U.M.L. (2001). Lift-Based Paddling Diving Grebe. J. Exp. Bio..

[88] Carroll N.R., Chiappe L.M., Bottjer D.J. (2019). Mid-Cretaceous amber inclusions reveal morphogenesis of extinct rachis-dominated feathers. Sci. Rep..

[89] Mayr G. (2016). The early Eocene birds of the Messel fossil site: A 48 million-year-old bird community adds a temporal perspective to the evolution of tropical avifaunas. Biol. Rev..

[90] Davis P.G., Briggs D.E.G. (1998). The Impact of Decay and Disarticulation on the Preservation of Fossil Birds. PALAIOS.

[91] Lovvorn J.R., Liggins G.A. (2002). Interactions of body shape, body size and stroke-acceleration patterns in costs of underwater swimming by birds. Funct. Ecol..

[92] Böhmer C., Plateau O., Cornette R., Abourachid A. (2019). Correlated evolution of neck length and leg length in birds. R. Soc. Open Sci..

[93] Nagai H., Mak S.-S., Weng W., Nakaya Y., Ladher R., Sheng G. (2011). Embryonic development of the emu, *Dromaius novaehollandiae*. Dev. Dyn..

[94] Jarvis E., Mirarab S., Aberer A.J., Li B., Houde P., Li C., Ho S.Y.W., Faircloth B.C., Nabholz B., Howard J.T. (2014). Whole-genome analyses resolve early branches in the tree of life of modern birds. Science.

[95] Jetz W., Thomas G.H., Joy J.B., Hartmann K., Mooers A.O. (2012). The global diversity of birds in space and time. Nature.

[96] Prum R.O., Berv J.S., Dornburg A., Field D.J., Townsend J.P., Lemmon E.M., Lemmon A.R. (2015). A comprehensive phylogeny of birds (Aves) using targeted next-generation DNA sequencing. Nature.

[97] d’Arcy W.T. (1890). On the Systematic Position of *Hesperornis*. Univ. Coll. Dundee Stud. Mus. Zool..

[98] Wilcox H.H. (1952). The Pelvic Musculature of the Loon, *Gavia immer*. Am. Midl. Nat..

[99] Mlíkovskỳ J. (1982). Evolution of Flightlessness in Birds: An Ecological Approach. Evol. Environ..

[100] Lovvorn J.R., Jones D.R. (1994). Biomechanical Conflicts between Adaptations for Diving and Aerial Flight in Estuarine Birds. Estuaries.

[101] Ribak G., Weihs D., Arad Z. (2008). Consequences of buoyancy to the maneuvering capabilities of a foot-propelled aquatic predator, the great cormorant (*Phalcrocorax carbo sinensis*). J. Exp. Biol..

[102] Lovvorn J.R. (1991). Mechanics of Underwater Swimming in Foot-Propelled Diving Birds. Proc. Int. Ornithol. Congr..

[103] Cook T.R., Lescroël A., Cherel Y., Kato A., Bost C.-A. (2013). Can Foraging Ecology Drive the Evolution of Body Size in a Diving Endotherm?. PLoS ONE.

