# Supplementary material for: Anatomy of Parahesperornis: Evolutionary Mosaicism in the Cretaceous Hesperornithiformes (Aves)
Source: Life (Basel). 2020 May 14;10(5):62. doi: 10.3390/life10050062 (PMC7281208; doi:10.3390/life10050062)
Supplement: Supplementary file 1 [file life-10-00062-s001.pdf]

Supplementary Materials

Table S1. Collected measurements of *Parahesperornis alexi* and other hesperornithiform birds. Measurements were collected from the specimens noted using digital calipers, unless otherwise noted.

	Parahesperornis alexi	*Parahesperornis alexi*	*Hesperornis regalis*	*Hesperornis gracilis*	*Baptornis advenus*	*Fumicollis hoffmani*	*Brodavis varneri*	*Pasquiaornis tankei*	*Enaliornis barretti*
Frontal	KUVP 2287	KUVP 24090	KUVP 71012	-	-	UNSM 20030	SDSM 68430	RSM P2995.4†	-
Maximum mediolateral width	40.68	-	39.74*	-	-	-	-	28.24*	-
Anterior-posterior length	53.94*	-	68.73*	-	-	-	-	34.78*	-
Premaxilla	KUVP 2287	KUVP 24090	KUVP 71012	-	-	UNSM 20030	SDSM 68430	-	-
Mediolateral width at nares	19.90	-	20.08	-	-	-	-	-	-
Anterior-posterior length	108.80*	-	171.67*	-	-	-	-	-	-
Length, opening of nares to rostral end	53.37	-	84.67	-	-	-	-	-	-
Coracoid	KUVP 2287	KUVP 24090	FHSM VP2069	-	KUVP 2290	UNSM 20030	SDSM 68430	RSM P2988.9†	-
Length	-	55.38	53.88	-	52.93	-	-	50.68	-
Width of sternal end	23.31*	33.51	36.7	-	26.64	-	-	20.59*	-
Humerus	KUVP 2287	KUVP 24090	FHSM VP2293	-	KUVP 2290	UNSM 20030	SDSM 68430	RSM P2077.4[1]	-
Distal mediolateral width	4.63	-	5.4*	-	6.68	-	-	11.04	-
Maximum preserved shaft width	5.01	-	7.44	-	5.48	-	-	4.90	-
Ilium	KUVP 2287	KUVP 24090	YPM 1476	YPM 1679	SDSM 5314	UNSM 20030	SDSM 68430	RSM P2626.27†	BGS 87936[4]
Length	237.63*	246.6	307.47*	-	-	198.04*	-	-	-
Ratio of pre- to post-acetabular length	0.45	0.31	0.39*	-	-	0.32*	-	-	-
Height at acetabulum	27.36	29.71	48.55	-	29.60	16.13	-	-	-
Acetabulum diameter	17.38	18.18	28.26	16.12*	11.85	13.58	16.64	18.15	13.51
Antitrochanter width	11.03	12.77	21.04	17.56	7.90	10.50	-	11.27	-

	Parahesperornis alexi	*Parahesperornis alexi*	*Hesperornis regalis*	*Hesperornis gracilis*	*Baptornis advenus*	*Fumicollis hoffmani*	*Brodavis varneri*	*Pasquiaornis tankei*	*Enaliornis barretti*
Femur	KUVP 2287	KUVP 24090	YPM 1200	YPM 1679	KUVP 2290	UNSM 20030	SDSM 68430	RSM P2077.108[2]	BMNH A483
Length	68.8	74.53	97.92	84.06	74.90	71.8	-	64.76	-
Proximal mediolateral width	33.59	24.02	52.98	45.06*	26.56	23.58	-	20.85	-
Midshaft mediolateral width	12.53	13.89	17.98	15.82	13.07	11.73	-	12.13	8.36*
Distal mediolateral width	32.26	33.64	52.81	47.75	24.57	25.24	31.08	21.73	16.02*
Width of fibular trochlea	9.27	9.78	16.5	15.23	9.81	9.19	11.39	8.95	6.80
Tibiotarsus	KUVP 2287	KUVP 24090	YPM 1200	YPM 1679	FMNH 395	UNSM 20030	SDSM 68430	RSM P2957.22†	BMNH A478
Length	212.97	230.94	321.11	-	194.76	193.82	226.64	-	-
Height of cnemial expansion	12.57	14.61	29.62	-	13.58	18.14	19.98	12.00	9.65
Proximal mediolateral width	23.69	21.26	34.83	34.00	16.07	18.70	24.92	16.81	10.97
Midshaft mediolateral width	14.86	15.08	24.64	20.75	10.23	12.49	17.20	11.80	9.21*
Distal mediolateral width	23.38	24.59	26.63	28.79	16.78	18.87	25.34	-	-
Fibula	KUVP 2287	KUVP 24090	YPM PU17193	YPM 1679	KUVP 2290	UNSM 20030	SDSM 68430	RSM P2997.80†	-
Length	130.71	-	200.2	-	-	-	-	-	-
Proximal craniocaudal width	13.07	-	19.28	17.41	13.48	10.68	12.74	8.87	-
Patella	KUVP 2287	KUVP 24090	YPM 1200	YPM 1679	KUVP 2290	UNSM 20030	SDSM 68430	-	-
Length	44.61	56.54	108.54	88.24*	20.51	21.11	-	-	-
Proximal width	22.24	26.72	42.25	30.11	15.26	17.31	-	-	-
Tarsometatarsus	KUVP 2287	FHSM VP17312	YPM 1200	YPM 1679	AMNH 5101	UNSM 20030	SDSM 68430	RSM P2077.63[3]	BMNH A477
Length	100.73	99.93	136.88	126.44	89.9	83.72	96.03	85.60	-
Proximal mediolateral width	21.35	17.35	35.65	29.28	19.11	18.79	25.59	15.29	-
Trochlea IV mediolateral width	10.10	8.66	14.72	13.17	6.77	5.13	6.67	6.05	5.51

Trochlea III mediolateral width	6.51	5.33	8.41	6.58	6.52	5.12	7.48	-	4.10
	Parahesperornis alexi	***Parahesperornis alexi***	***Hesperornis regalis***	***Hesperornis gracilis***	***Baptornis advenus***	***Fumicollis hoffmani***	***Brodavis varneri***	***Pasquiaornis tankei***	***Enaliornis barretti***
Digit IV: 1	KUVP 2287	-	YPM 1200	YPM 1679	FMNH 395	UNSM 20030	SDSM 68430	-	-
Length	29.17	-	44.22	40.32	36.55	-	-	-	-
Proximal dorsoplantar depth	13.56	-	22.81	18.38	6.39	-	-	-	-
Proximal mediolateral width	12.02	-	18.70	15.64	8.06	-	-	-	-
Digit III: 1	KUVP 2287	-	YPM 1200	YPM 1478	FMNH 395	UNSM 20030	SDSM 68430	-	-
Length	31.14	-	40.24	34.87	23.73	37.77	-	-	-
Proximal dorsoplantar depth	7.20	-	11.40	13.94*	7.16	11.33	-	-	-
Proximal mediolateral width	12.51	-	16.58	-	4.58	6.01	-	-	-

*approximate; †measurement taken from photograph using ImageJ; [1] measured from cast YPM 57198; [2] measured from cast YPM 5719; [3] measured from cast YPM 57194; [4] measured from cast BMNH A5310.

Table S2. Specimens used for this study. The majority of specimens were examined directly; however, references are listed for specimens not available for examination during this study.

Genus	Species	Specimen Number	Material	Citation (if specimen not studied directly)
Asiahesperornis	*bazhanovi*	IZASK 2/KM 97	roughly 14th or 15th cervical vertebra	1
Asiahesperornis	*bazhanovi*	IZASK 4/KM 97[1]	partial dentary	1
Baptornis	*advenus*	YPM 1465*	distal tarsometatarsus (lectotype)	
Baptornis	*advenus*	AMNH 5101	cervical and thoracic vertebrae, partial pelvis, distal tibiotarsus, tarsometatarsi, partial phalanx.	
Baptornis	*advenus*	FMNH 395	caudal fragment of left mandible, 23rd vertebra, partial synsacrum & pelvic fragments, pygostyle, femora, tibiotarsi, tarsometatarsus, metatarsal I, 14 phalanges, including 2 unguals	
Baptornis	*advenus*	KUVP 2290	coracoid, humerus, ulna, radius, partial scapula, 9 vertebrae, partial synsacrum, femur, patella, fibulae, partial tibiotarsi, partial tarsometatarsus	

Baptornis	*advenus*	KUVP 16112	cervical and thoracic vertebra, partial synsacrum and pelvis, partial femora, partial tibiotarsi, partial tarsometatarsus, partial phalanges; juvenile	
Brodavis	*americanus*	RSM P2315.6* (unnumbered cast at KUVP)	tarsometatarsus	
Brodavis	*baileyi*	UNSM 50665*	tarsometatarsus	
Brodavis	*varneri*	SDSM 68430*	cervical and thoracic vertebrae, ribs, partial synsacrum and pelvis, partial femur, tibiotarsus, fibula, tarsometatarsus	
Canadaga	*arctica*	NMC 41050*	three cervical vertebrae, probably 15-17	2
Chupkaornis	*keraorum*	MCM.A773*	cervical and thoracic vertebrae, partial femora, fibula	3
Enaliornis	*barretti*	BMNH A477*	partial tarsometatarsus	
Enaliornis	*barretti*	BGS 87933 (cast as BMNH A5306)	partial femur	
Enaliornis	*barretti*	BGS 87936 (cast as BMNH A5310)	acetabular region of pelvis	
Enaliornis	*barretti*	BMNH A163	partial femur	
Enaliornis	*barretti*	BMNH A483	partial femur - juvenile	
Enaliornis	*barretti*	SMC B 54404	braincase	
Enaliornis	*barretti*	SMC B 55277	23rd vertebra	
Enaliornis	*barretti*	SMC B 55306	partial femur	
Enaliornis	*barretti*	SMC B 55310	partial femur	
Enaliornis	*sedgwicki*	BMNH A479	partial femur	
Enaliornis	*sedgwicki*	SMC B 55279	thoracic vertebra	
Enaliornis	*sedgwicki*	SMC B 55280	thoracic vertebra	
Enaliornis	*sedgwicki*	SMC B 55287	partial femur - juvenile	
Enaliornis	*sedgwicki*	SMC B 55289	partial femur	
Enaliornis	*sedgwicki*	SMC B 55300	partial femur	
Enaliornis	*seeleyi*	BMNH A478	partial tibiotarsus	
Enaliornis	*seeleyi*	BMNH A5801	partial femur	
Enaliornis	*seeleyi*	SMC B 55321	partial tarsometatarsus - juvenile	
Fumicollis	*hoffmani*	UNSM 20030*	cervical and thoracic vertebrae, pygostyle, rib fragments, partial pelvis, femur, patella, fibula, tibiotarsi, tarsometatarsus, 6 pedal phalanges	
Hesperornis	*chowi*	YPM PU 17208*	tarsometatarsus	4
Hesperornis	*crassipes*	YPM 1474*	Nearly complete skeleton	5
Hesperornis	*gracilis*	YPM 1473*	partial tarsometatarsus, 2 pedal phalanges	

Hesperornis	*gracilis*	YPM 1478	thoracic vertebrae, partial femur, partial tibiotarsus, tarsometatarsus, 2 pedal phalanges	
Hesperornis	*gracilis*	YPM 1679	cervical and thoracic vertebrae, partial synsacrum and pelvis, rib fragments, femora, patella, fibula, tibiotarsi, tarsometatarsi, 3 pedal phalanges	
Hesperornis	*lumgairi*	CFDC B.78.02.07*	tarsometatarsus	
Hesperornis	*macdonaldi*	LACM 9728*	femur	
Hesperornis	*macdonaldi*	LACM 9727	femur	
Hesperornis	*macdonaldi*	CFDC B.81.03.16	femur	6
Hesperornis	*mengeli*	CFDC B.78.01.08[2]	tarsometatarsus	
Hesperornis	*regalis*	YPM 1200*	cervical, thoracic, and caudal vertebrae, femora, tibiotarsi, tarsometatarsi, patellae, fibulae, 2 pedal phalanges	
Hesperornis	*regalis*	BMNH A-720	partial pelvis	
Hesperornis	*regalis*	FHSMNH 2069	cervical and thoracic vertebrae, rib fragments, coracoids, femora, tibiotarsi, tarsometatarsi, patella, pedal phalanges	
Hesperornis	*regalis*	FR 2181	femur, tibiotarsus, patella, fibula	
Hesperornis	*regalis*	YPM 1206	partial skull and lower jaw, cervical and thoracic vertebrae	5
Hesperornis	*regalis*	YPM 1207	cervical and thoracic vertebrae, partial synsacrum and pelvis, partial coracoid, femora, partial tibiotarsi, partial fibulae, patellae, tarsometatarsi, 2 pedal phalanges	
Hesperornis	*regalis*	YPM 1476	cervical and thoracic vertebrae, synsacrum and pelvis, partial sternum, femur, patella, tarsometatarsi, tibiotarsi, pedal phalanges	
Hesperornis	*regalis*	YPM 1477	cervical and thoracic vertebrae, rib fragments, partial coracoid, femora, tibiotarsi, fibulae, patella	
Hesperornis	*sp.*	FHSMNH 2293	thoracic vertebrae, rib fragments, humerus, scapula, partial femur, patella, partial tibiotarsus,	
Hesperornis	*sp.*	KUVP 71012	partial skull and lower jaw, axis and cervical vertebrae, tarsometatarsus; 11 pedal phalanges	
Hesperornis	*sp.*	SDSM 5312	cervical and thoracic vertebrae, partial pelvis, femur, patella, tibiotarsus, tarsometatarsus, 4 pedal phalanges	
Hesperornis	*sp.*	YPM 1499	cervical and thoracic vertebrae, femora, fibulae, patellae, tibiotarsus, tarsometatarsus	
Hesperornis	*sp.*	YPM PU 17193	femur, fibula, patella, tibiotarsus	

Hesperornis	*sp.*	YPM PU 18589	cervical and thoracic vertebrae, sternal fragment, clavicle, femur, patellae, tibiotarsus	
Parahesperornis	*alexi*	KUVP 2287*	partial skull and lower jaw, cervical and thoracic vertebrae, synsacrum and pelvis, coracoid, humerus, sternal fragments, femora, patella, fibula, tibiotarsi, tarsometatarsi, 26 pedal phalanges, skin impressions	
Parahesperornis	*alexi*	KUVP 24090	cervical, thoracic, and free caudal vertebrae, synsacrum and pelvis, pygostyle, coracoid, scapula, sternal fragments, femora, patellae, fibulae, tibiotarsi	
Parahesperornis	*sp.*	FHSM VP-17312	tarsometatarsus	
Pasquiaornis	*hardiei*	RSM P2077.117 (cast as YPM 57191)	partial tarsometatarsus	7
Pasquiaornis	*hardiei*	RSM P2077.59 (cast as YPM 57199)	partial femur	
Pasquiaornis	*hardiei*	RSM P2077.60 (cast as YPM 57192)	partial femur	
Pasquiaornis	*hardiei*	RSM P2487.3 (cast as YPM 57198)	partial humerus	
Pasquiaornis	*hardiei*	RSM P2831.6	partial dentary	7
Pasquiaornis	*hardiei*	RSM P2831.8	cervical vertebra	7
Pasquiaornis	*hardiei*	RSM P2985.9	splenial	7
Pasquiaornis	*hardiei*	RSM P2989.19	partial mandible	7
Pasquiaornis	*hardiei*	RSM P2997.18	tarsometatarsus	7
Pasquiaornis	*hardiei*	RSM P2997.4	femur	7
Pasquiaornis	*hardiei*	RSM P2997.81	partial tarsometatarsus	7
Pasquiaornis	*tankei*	RSM P2077.10 (cast as YPM 57197)	partial femur	
Pasquiaornis	*tankei*	RSM P2077.4 (cast as YPM 57193)	partial humerus	
Pasquiaornis	*tankei*	RSM P2077.79	partial tarsometatarsus	
Pasquiaornis	*tankei*	RSM P2077.79	partial tarsometatarsus	7, 8
Pasquiaornis	*tankei*	RSM P2957.15	thoracic vertebra	7
Pasquiaornis	*tankei*	RSM P2957.21	partial tibiotarsus	7
Pasquiaornis	*tankei*	RSM P2957.22	partial tibiotarsus	7
Pasquiaornis	*tankei*	RSM P2985.1	thoracic vertebra	7
Pasquiaornis	*tankei*	RSM P2988.11	partial dentary	7
Pasquiaornis	*tankei*	RSM P2988.12	cervicothoracic vertebra	7
Pasquiaornis	*tankei*	RSM P2988.25	partial quadrate	7
Pasquiaornis	*tankei*	RSM P2988.9	coracoid	7

Pasquiaornis	*tankei*	RSM P2989.21	partial mandible	7
Pasquiaornis	*tankei*	RSM P2995.1	humerus	7
Pasquiaornis	*tankei*	RSM P2995.4	partial frontal	7
Pasquiaornis	*tankei*	RSM P2997.60	partial scapula	7
Potamornis	*skutchi*	UCMP 73103	partial quadrate	

*type specimen; [1] Incorrect identification; here revised to Aves *incertae sedis*; [2] incorrectly reported as BO 780106 in Martin and Lim, 2002.

References

1. Dyke, G. J.; Malakhov, D. V.; Chiappe, L. M. A Re-Analysis of the Marine Bird Asiahesperornis from Northern Kazakhstan. *Cretac. Res.* **2006**, *27*, 947–953.
2. Hou, L.-I. New hesperornithid (Aves) from the anadian arctic. *Vertebr. PalAsiat.* **1999**, *37*, 231–241.
3. Tanaka, T.; Kobayashi, Y.; Kurihara, K.; Fiorillo, A. R.; Kano, M. The Oldest Asian Hesperornithiform from the Upper Cretaceous of Japan, and the Phylogenetic Reassessment of Hesperornithiformes. *J. Syst. Palaeontol.* **2017**, *16*, 689–709.
4. Martin, L. D.; Lim, J.-D. New Information on the Hesperornithiform Radiation. In *Proceedings of the 5th Symposium of the Society of Avian Paleontology and Evolution, Beijing, 1-4 June 2000*; Science Press: Beijing, China, 2002.
5. Marsh, O. C. Odontornithes: A Monograph on the Extinct Toothed Birds of North America; US Government Printing Office: Washington, D.C., USA, 1880; Vol. 1.
6. Aotsuka, K.; Sato, T. Hesperornithiformes (Aves: Ornithurae) from the Upper Cretaceous Pierre Shale, Southern Manitoba, Canada. *Cretac. Res.* **2016**, *63*, 154–169.
7. Sanchez, J. Late Cretaceous (Cenomanian) Hesperornithiformes from the Pasquia Hills, Saskatchewan, Canada. Master of Science, Carleton University, Ottawa, Ontario, 2010.
8. Tokaryk, T. T.; Cumbaa, S. L.; Storer, J. E. Early Late Cretaceous Birds from Saskatchewan, Canada: The Oldest Diverse Avifauna Known from North America. *J. Vertebr. Paleontol.* **1997**, *17*, 172–176.
